# A Closed Cavity Strategy for Selective Dipeptide Binding by a Polyaromatic Receptor in Water

**DOI:** 10.1021/jacsau.3c00484

**Published:** 2023-10-02

**Authors:** Mayu Shuto, Ryuki Sumida, Mana Yuasa, Tomohisa Sawada, Michito Yoshizawa

**Affiliations:** Laboratory for Chemistry and Life Science, Institute of Innovative Research, Tokyo Institute of Technology, 4259 Nagatsuta, Yokohama 226-8503, Japan

**Keywords:** coordination capsule, dipeptide, recognition, host−guest interactions, water

## Abstract

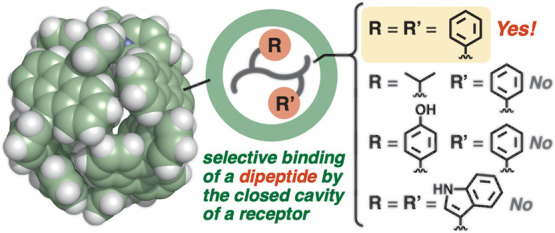

Precise recognition of peptides is a daunting task owing to the substantial number of available amino acids and their combination into various oligo/polymeric structures in addition to the high hydration of their flexible frameworks. Here, we report the selective recognition of a dipeptide through a *closed cavity strategy*, in contrast to previous synthetic receptors with open cavities. A polyaromatic receptor with a virtually isolated, hydrophobic cavity exclusively binds one molecule of phenylalanine dipeptide from a mixture with its amino acid and tripeptide in water via multiple CH−π and hydrogen-bonding interactions in the complementary cavity. The binding selectivity persists even in the presence of other dipeptides, such as leucine–leucine, leucine–phenylalanine, tyrosine–phenylalanine, tryptophan–tryptophan, and aspartame, revealed by NMR/MS-based competitive binding experiments. ITC studies reveal that the selective binding of the phenylalanine dipeptide is relatively strong (*K*_a_ = 1.1 × 10^5^ M^–1^) and an enthalpically and entropically favorable process (Δ*H* = −11.7 kJ mol^–1^ and *T*Δ*S* = 17.0 kJ mol^–1^). In addition, the present receptor can be used for the emission detection of the dipeptide through a combination with a fluorescent dye in water.

## Introduction

**Figure 1:**
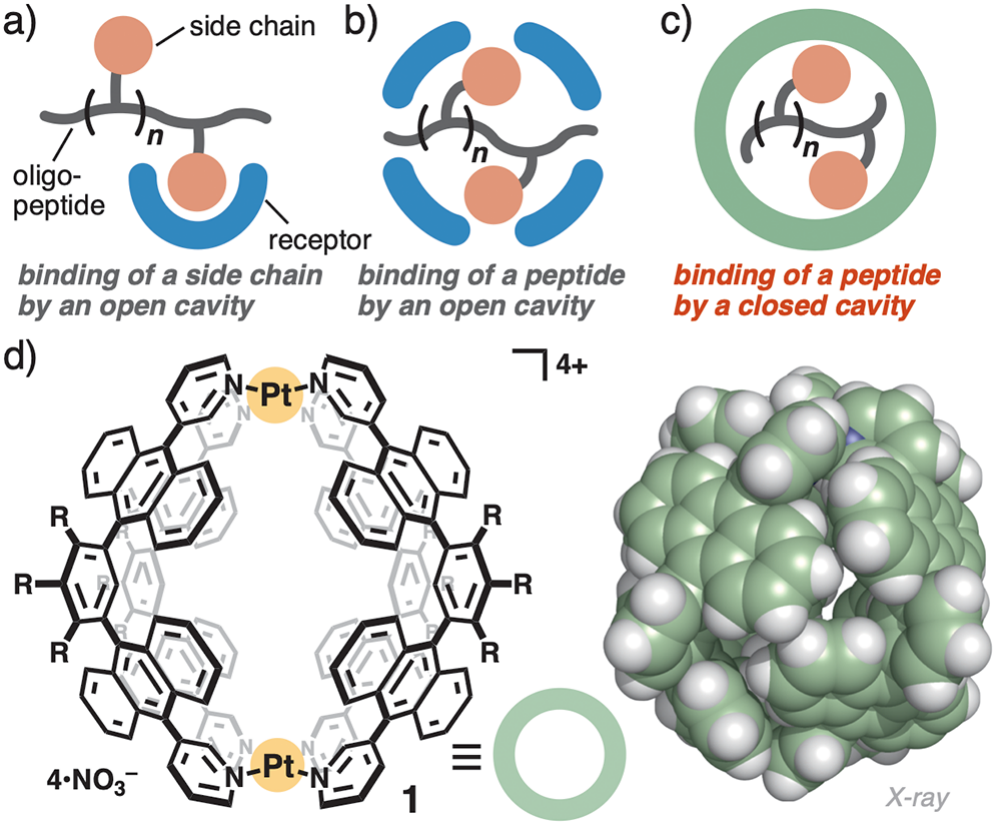
Recognition of an oligopeptide by synthetic receptors with (a, b) open cavities and (c) a closed cavity, studied herein. (d) Polyaromatic receptor **1** with a virtually closed cavity (R = OCH_2_CH_2_OCH_3_) and its crystal structure (R = H for clarity).

Peptides are the oligo/polymers of amino acids, with hydrophilic amide chains as well as amino and carboxy terminal groups.^1^ Through combination of available natural amino acids, the oligomers provide plenty of derivatives, depending on the number, kind, and order of residues, flexible frameworks, and high hydrophilicity.^1,2^ Owing to these properties, the precise recognition of oligopeptides with synthetic receptors remains extremely difficult, as compared with other biomolecules.^3^ The previously reported receptors can be divided into two types, depending on the recognition mode: the binding of a single or two side chain(s) (Figure 1a)^4−6^ or the partial or full inclusion of a peptide (Figure 1b).^7^ In all cases, synthetic receptors with open cavities, such as aliphatic/small aromatic tubular, clip-like, and cage-shaped compounds, have been used for the biomolecular recognition. There have been a few reports on innovative coordination receptors^8^ and macrocyclic receptors (i.e., cucurbiturils),^5b−5d^ capable of binding specific amino acid sequences in the open cavities. However, the strict distinction of number and kind of the residues of oligopeptides as well as the selective binding of target oligopeptides from mixtures with other derivatives are still daunting tasks for previous synthetic receptors.^4−8^

Here, we propose a new strategy for the selective recognition of oligopeptides using *a closed cavity* (Figure 1c). Synthetic polyaromatic receptor **1** employed herein possesses a well-defined spherical cavity, encircled by multiple polyaromatic panels (Figure 1d).^10,11^ Unlike previous open receptors, the virtually isolated cavity allows the present receptor to recognize oligopeptides based on the number and kind of amino acid residues with high selectivity. In this report, we demonstrate (i) the selective binding of one molecule of a phenylalanine dipeptide (**FF**) by receptor **1**, even from mixtures with amino acids and other oligopeptides in water. (ii) Detailed ITC, NMR, IR, and theoretical calculation analyses reveal that moderate enthalpic and entropic stabilization and multiple CH−π and hydrogen-bonding interactions in the complementary cavity are key driving forces for the high selectivity. Furthermore, (iii) selective emission detection of the dipeptide can be accomplished using **1** and a fluorescent coumarin dye in water.

Synthetic receptor **1** is a spherical supramolecular capsule, composed of two metal ions and four bent polyaromatic ligands, usable under ambient aqueous conditions.^10^ Besides typical hydrophobic guest molecules (e.g., adamantanes, pyrenes, and fullerene C_60_),^11^ its virtually closed cavity (∼1.3 nm in diameter and ∼580 Å^3^ in volume) displays efficient binding abilities toward hydrophilic oligomers,^12^ such as synthetic oligo(ethylene glycol)s and biorelated oligo(lactic acid)s, through the small yet flexible gaps (∼0.3 nm windows) in water at room temperature, without the ligand dissociation. However, the length-selective recognition of such oligomers by the receptor has been unachieved so far, owing to their flexibility and none/small substituents. We posited that oligopeptides, bearing similar flexible frameworks yet characteristic side chains (e.g., aromatic and aliphatic groups), would be promising new targets for receptor **1** with high binding selectivity. Importantly, this study is focused on unprotected natural oligopeptides rather than *N-* and *C*-termini-protected ones because the binding properties are largely affected and changed by the protecting groups.^9^

## Results and Discussion

### Selective Binding of Phenylalanine Dipeptide

Inspired by the selective binding ability of polyaromatic receptor **1** toward two molecules of benzene derivatives (e.g., mesitylene and xylene) in water at room temperature,^13^ phenylalanine (**F**) and its dipeptide **FF** and tripeptide **FFF** were selected as the first set of a mixture among a huge number of amino acid/oligopeptide combinations. Prior theoretical estimations by DFT calculations predicted that 1:1 host–guest structure **1**•**FF** should be the most stable product among the possible four products (i.e., **1**•**F**, **1**•(**F**)_2_, **1**•**FF**, and **1**•**FFF**; Table S1), due to the size and shape matching.^14,15^

**Figure 2:**
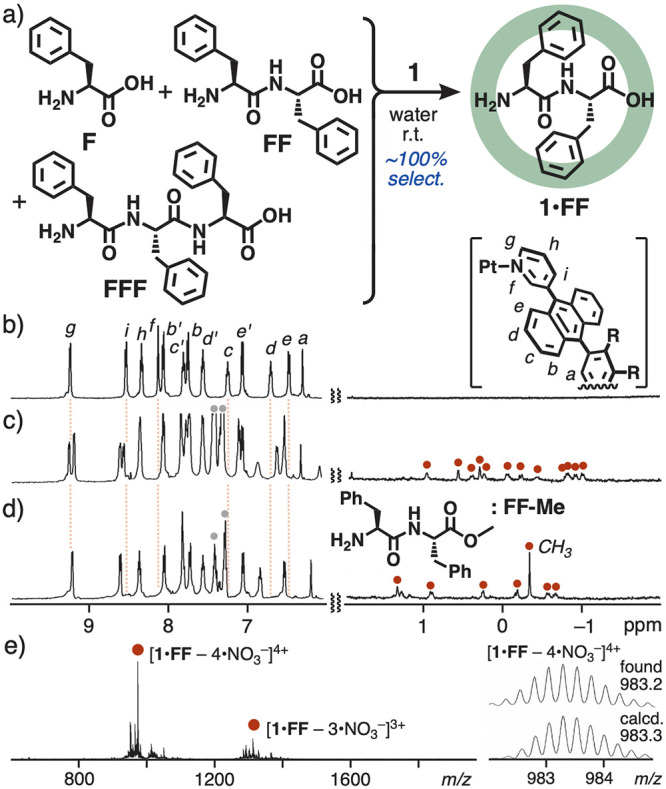
(a) Selective binding of phenylalanine dipeptide **FF** by **1** from a mixture of **F**, **FF**, and **FFF** in water at room temperature.^16^^1^H NMR spectra (500 MHz, D_2_O, r.t.) of (b) **1**, (c) product **1•FF** after the competitive binding experiment, and (d) **1•FF-Me** (gray circles: free guests). (e) ESI-TOF MS spectrum (H_2_O) of **1•FF** after the competitive binding experiment.

When a mixture of **1**, **F**, **FF**, and **FFF** (0.13 μmol each) was stirred in water (0.5 mL) at room temperature for 30 min, 1:1 host–guest structure **1**•**FF** was formed in a selective and quantitative fashion (Figure 2a).^16,17^ In the ^1^H NMR spectrum, the aromatic signals of empty **1** were fully converted to those of a new host–guest structure (Figure 2b,c). Whereas the phenyl signals of bound **FF** were fully overlapped with the methoxyethoxy side chain signals of **1**, complicated signals observed in the range of 1.0 to −1.0 ppm were assignable to the methylene and methine signals of bound **FF**, due to the consistence with those of **1**•**FF** prepared separately from **1** and **FF** under the same conditions (Figure S6a).^14^ The large upfield shifts of the aliphatic signals (Δδ_max_ = approximately −4 ppm) are derived from the effective aromatic shielding effect by **1**. The splitting of the aliphatic signals from 6 to 12 upon binding indicates tight host–guest interactions in the desymmetrized polyaromatic cavity,^18^ through efficient carbonyl-based hydrogen-bonding interactions, as discussed later. The ESI-TOF MS spectrum of the resultant solution displayed molecular ion peaks, corresponding to **1**•**FF** (i.e., *m*/*z* = 983.2 for [**1**•**FF** – 4•NO_3_^–^]^4+^ and 1331.7 for [**1**•**FF** – 3•NO_3_^–^]^3+^; Figure 2e). MS peaks for not only empty **1** but also **1**•(**F**)_*n*_ and **1**•**FFF** were hardly detected in the spectrum. To our surprise, neither **F** nor **FFF** was bound by **1** under the same conditions, separately and thus, even from a complex mixture of **F**, **FF**, and **FFF** in a 5:1:5 ratio, receptor **1** bound **FF** in an exclusive manner (Figure S7a,c).^14^ The quantitative binding of **FF** by the receptor was found even in an acetic acid buffer solution (Figure S6b). To support the weak NMR signals of bound **FF**, *C*-terminal methyl-protected phenylalanine dipeptide **FF-Me** was used as a reference guest (Figure 2d). Its host–guest structure **1**•**FF-Me**, formed quantitatively from **1** and **FF-Me** in water at room temperature, showed a sharp proton signal for the CH_3_ group at −0.33 ppm, with a large upfield shift (Δδ = −4.0 ppm; Figure 2d) as compared with free **FF-Me**, due to its complete encapsulation.

### Thermodynamic Studies and Host–Guest Interactions

**Figure 3:**
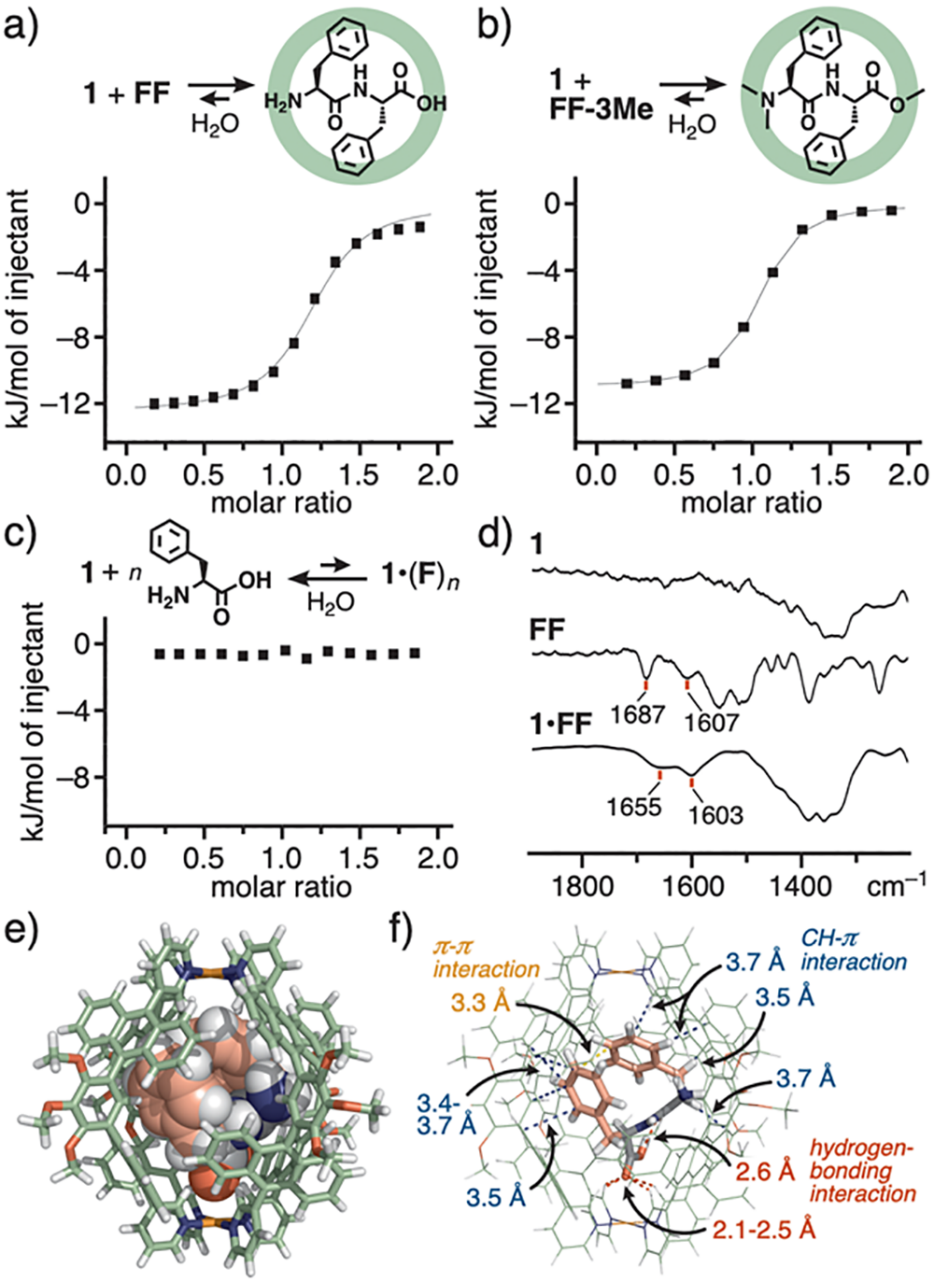
ITC thermograms (H_2_O, 298 K) for (a) **1** + **FF**, (b) **1** + **FF-3Me**, and (c) **1** + *n***F**.^14^ (d) FT-IR spectra (ATR, r.t.) of **1**, **FF**, and **1**•**FF**. (e) Optimized structure of **1**•**FF** (PM6 calculation, R = OCH_3_) and (f) the host–guest interactions (blue, red, and orange dotted lines are derived from CH−π, hydrogen-bonding, and π–π interactions, respectively).

As quantitative experimental data, the binding constant (*K*_a_) and thermodynamic parameters (Δ*H* and *T*Δ*S*) of **1** toward **FF** were estimated by ITC analysis.^14^ The titration study in water at 25 °C revealed that the host–guest interactions are relatively high (*K*_a_ = 1.07 × 10^5^ M^–1^; Figure 3a).^19^ The guest binding is both an enthalpically and an entropically favorable process (Δ*H* = −11.7 kJ mol^–1^ and *T*Δ*S* = 17.0 kJ mol^–1^; Table 1). The enthalpic energy gain most probably stems from host–guest CH−π and hydrogen-bonding interactions as well as the replacement of high-energy water molecules from the cavity.^20^ The FT-IR analysis of **1**•**FF** suggested the presence of strong chelate-type hydrogen-bonding interactions between one of the two carbonyl groups on **FF** and the pyridyl α-protons on **1** (Δν_C=O_ = −32 and −4 cm^–1^, Figure 3d),^12b^ whereas the interactions are relatively weak in **1**•**FF-Me** (Δν_C=O_ = −6 and −3 cm^–1^, Figure S8c). Weak host–guest π–π interactions were supported by the UV–visible study of **1**•**FF**, where the anthracene-based absorption bands (λ = 300–420 nm) remain virtually intact (Δλ_max_ = +1 nm, Figure S6d).^14^ The entropic energy gain indicates the dehydration of the host cavity and the guest surface upon inclusion. As we expected, ITC studies revealed that *C*-terminal and *C,N*-terminal protections on **FF** with methyl groups enhance the host–guest interactions (2.5-fold for **FF-Me** and 4.4-fold for **FF-3Me** based on *K*_a_) with **1** under the same conditions (Figure 3b and Table 1), likely due to additional hydrophobic CH−π interactions. No host–guest interactions between **1** and **F** were evidently confirmed by ITC analysis (Figure 3c).

**Table 1:** Binding Constants (*K*_a_) and Thermodynamic Parameters for the Formation of **1**•**FF**, **1**•**FF-Me**, **1**•**FF-3Me**, and **1**•**WF** Obtained by ITC Experiments (H_2_O, 298 K)^14^

entry	*K*_a_ [M^–1^]	Δ*H* [kJ mol^–1^]	*T*Δ*S* [kJ mol^–1^]	Δ*G* [kJ mol^–1^]
**1**•**FF**	(1.07 ± 0.15) × 10^5^	–11.7 ± 0.19	17.0	–28.7
**1**•**FF-Me**	(2.68 ± 0.84) × 10^5^	–6.61 ± 0.29	24.4	–31.0
**1**•**FF-3Me**	(4.63 ± 0.61) × 10^5^	–12.0 ± 0.17	20.2	–32.2
**1**•**WF**	(2.59 ± 0.41) × 10^4^	–10.0 ± 0.56	–15.1	–25.1

Detailed host–guest interactions were investigated using the optimized structure of **1**•**FF**, obtained in the initial theoretical studies, owing to its poor crystallinity. In the spherical cavity of **1**, bound **FF** adopts a spherical conformation through folding of the peptide backbone (Figure 3e), displaying high host–guest complementarity in size and shape. The folded structure of **FF** is fully surrounded by the polyaromatic framework of **1**. There is no space to encapsulate any tripeptides or oligopeptides with an **FF** sequence in the virtually closed cavity. On the basis of close distances, the presence of eight CH−π interactions (3.4–3.7 Å)^21^ and four hydrogen-bonding interactions (2.1–2.5 Å) was indicated between **1** and **FF** in the isolated cavity (Figure 3f), which were supported by the ^1^H NMR, FT-IR, and UV–visible studies, as described above.

### Recognition of Aromatic vs Aliphatic Side Chains on Dipeptides

**Figure 4:**
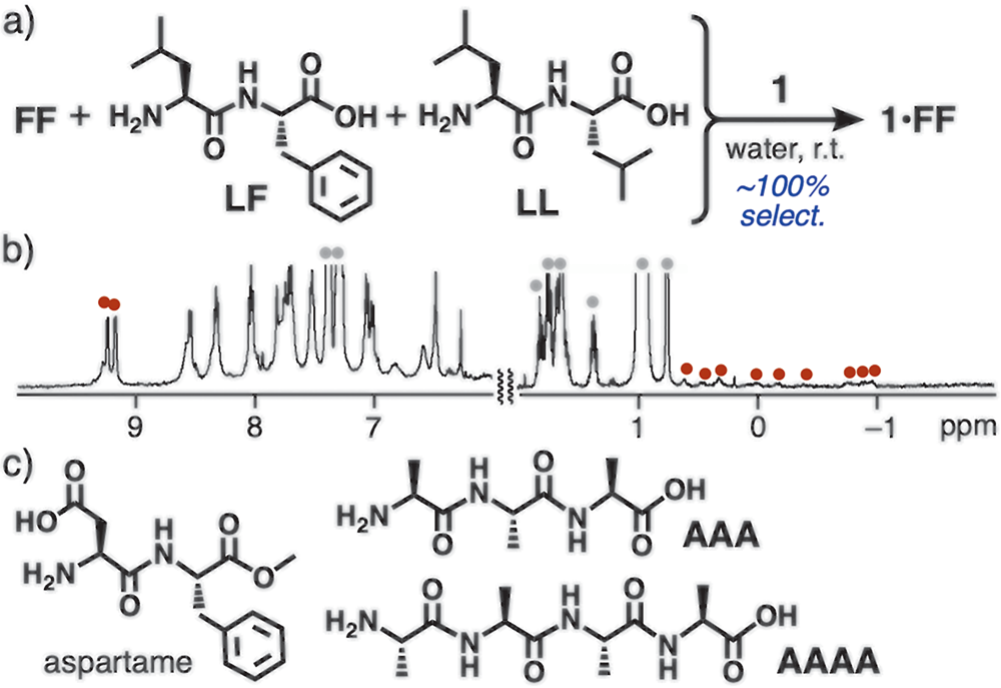
(a) Selective binding of **FF** by **1** from mixture of **FF**, **LF**, and **LL** in water at room temperature. (b) ^1^H NMR spectrum (500 MHz, D_2_O, r.t.) of the competitive binding experiment from mixture of **1** with **FF**, **LF**, and **LL** in water after 30 min (gray circles: free guests). (c) Oligopeptides aspartame, **AAA**, and **AAAA**, used in this study.

Next, the ability of **1** to distinguish between *aromatic* and *aliphatic* side chains on dipeptides was verified using leucine–phenylalanine (**LF**) and leucine–leucine (**LL**), bearing hydrophobic isopropyl groups, potentially interacting with the receptor cavity through multiple CH−π interactions. Stirring a mixture of dipeptides **FF**, **LF**, and **LL** (1.0 equiv. each) with receptor **1** in water (0.5 mL) for 30 min at room temperature led to the selective and quantitative formation of **1**•**FF** (Figure 4a). The product structure was confirmed by the ^1^H NMR and ESI-TOF MS spectra (Figure 4b and Figure S13b, respectively).^14^ Host–guest interactions between **1** and **LF** or **LL** were scarcely observed even when adding excess amount of **LF** or **LL** (5.0 equiv) to **1** in water (Figure S13a,c).

Notably, the exclusive binding of **FF** was demonstrated by **1**, even from a mixture of **FF** and aspartame, known as a representative dipeptide-based artificial sugar, under the same conditions (Figure 4c and Figure S10).^12a,14^ The binding selectivity persisted in the presence of long aliphatic oligopeptides, such as alanine trimer **AAA** and tetramer **AAAA**, with multiple methyl groups (Figure 4c and Figure S13d).

### Recognition of Aromatic vs Aromatic Side Chains on Dipeptides

**Figure 5:**
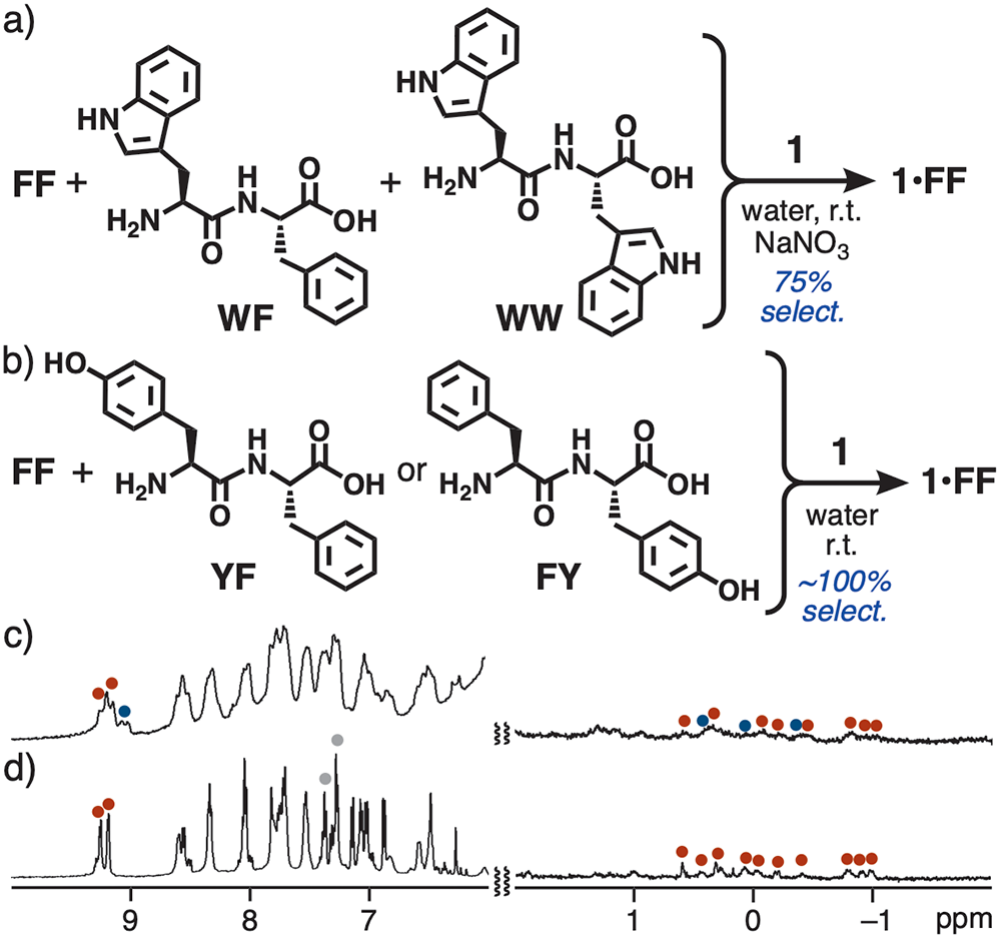
Selective binding of **FF** by **1** from mixtures of a) **FF**, **WF**, and **WW** and (b) **FF** and **YF** or **FY** in water at room temperature. ^1^H NMR spectra (500 MHz, D_2_O, r.t.) of the competitive binding experiments from mixtures of **1** with (c) **FF**, **WF**, and **WW** (in the presence of NaNO_3_) and (d) **FF** and **YF** in water after 30 min (gray circles: free guests).

The distinction between *aromatic* and *aromatic* side chains on the dipeptides was further examined. Tryptophan (**W**) provides a heterocyclic indole ring capable of efficiently interacting with aromatic and aliphatic surfaces so that **W** and its oligomers have been preferentially bound by the majority of previous synthetic receptors so far.^3−5,22^ In contrast, the closed polyaromatic cavity of **1** could capture **FF** with 75% selectivity from a mixture of **FF**, **WF**, and **WW** as well as with 100% selectivity from a mixture of **FF** and **WW**. When a mixture of **1**, **FF**, **WF**, and **WW** (1.0 equiv. each) was stirred in water in the presence of NaNO_3_ (0.2 equiv. based on **1**) at room temperature for 30 min, the quantitative formation of host–guest structures **1**•**FF** and **1**•**WF** was confirmed by ^1^H NMR and ESI-TOF MS analyses (Figure 5a and Figure S18, respectively). These spectra indicated the absence of both empty **1** and host–guest structure **1**•**WW**. Observed aliphatic proton signals in the range of 0.7 to −1.0 ppm were derived from bound **FF** and **WF** in the cavity of **1** (Figure 5c). The binding selectivity of **1** toward **FF** and **WF** was estimated to be 75:25 on the basis of the integration of proton *H*_g_ (at ∼9.2 ppm) on the receptor.^14^ It is worth noting that the simple addition of the nitrate salt improved the selectivity by 1.4-fold, most probably due to the slight shrinking of the polyaromatic shell at high ionic strength. From a 1:1 mixture of **FF** and sequential isomer **FW**, receptor **1** bound **FF** in 79% selectivity (Figure S18), even without NaNO_3_ addition.^14^ Neither **1**•**WW** nor **1**•**W** was generated even by the treatment of **1** with **WW** or **W** under various aqueous conditions. These results revealed the detailed recognition order of **1** to be **FF** > **WF** > **FW** ≫ **WW** ≈ **W**. On the basis of ITC-based binding constants, host–guest interactions of **1** toward **WF** (*K*_a_ = 2.6 × 10^4^ M^–1^) are 4.1-fold weaker than those toward **FF** even without NaNO_3_ (Table 1 and Figure S22).^14^

Furthermore, among phenyl-based dipeptides, the absence and presence of a single hydroxy group were recognized by **1** in an exclusive way. From a 1:1 mixture of **FF** and tyrosine–phenylalanine (**YF**) with **1** in water at room temperature (Figure 5b), the ^1^H NMR and ESI-TOF MS analyses revealed the selective and quantitative formation of **1**•**FF** (Figure 5d and Figure S21).^14^ The selectivity stems from the steric bulkiness of the *para*-substituted OH group on the phenyl ring, which is unfit to the closed and rigid, spherical cavity.^23^ The exclusive binding of **FF** by **1** was also observed from a 1:1 mixture of **FF** and phenylalanine–tyrosine (**FY**) under the same conditions (Figure 5b and Figure S21).

### Selective Fluorescence Detection of Phenylalanine Dipeptide

**Figure 6:**
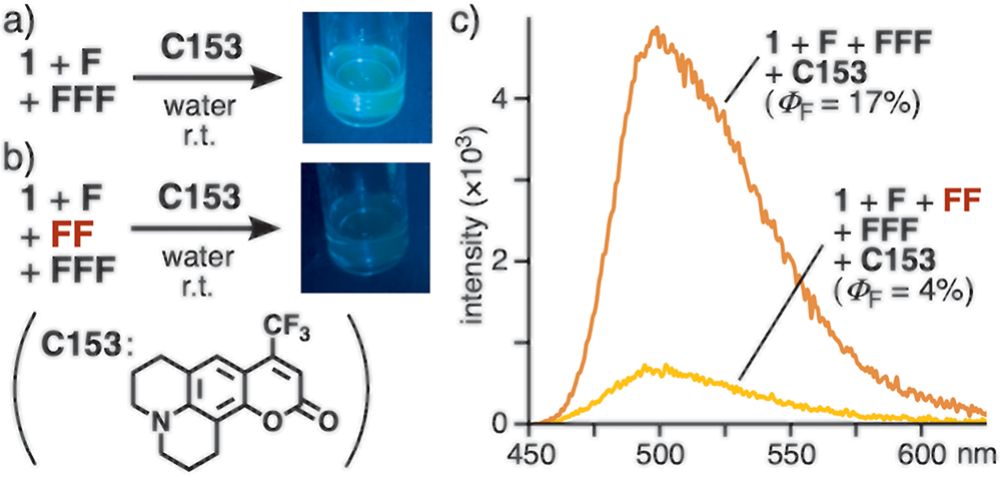
Fluorescence detection of **FF** for the mixture of **1**, **F**, and **FFF** in the (a) absence and (b) presence of **FF**, by the addition of **C153** in water (photographs, 0.2 mM based on **1**, λ_irrad_ = 455 nm), and (c) their emission spectra and quantum yields (r.t., λ_irrad_ = 423 nm).

With the aid of nonfluorescent receptor **1**, the facile yet sensitive detection of dipeptide **FF** was performed by the use of a fluorescent coumarin dye. Although hydrophobic coumarin **C153** employed here is water-insoluble under ambient conditions, aqueous host–guest structure **1**•**C153** emitted green fluorescence (Φ_F_ = 20%) in water upon light irradiation at 423 nm (Figure S23a). A similar strong emission was observed from an aqueous solution of **1**, **F**, and **FFF** (in a 1:1:1 ratio) after 5 min, upon addition of **C153** (2.0 equiv based on **1**), due to the formation of **1**•**C153** (Figure 6a). Under the same conditions, in sharp contrast, an aqueous solution of **1**, **F**, **FF**, and **FFF** showed no emission visible to the naked eye even after the addition of **C153** (Figure 6b). The spectroscopic analyses of the resultant solutions clarified large differences in the emission intensity (6.5-fold) and quantum yield (4.3-fold), depending on the absence and presence of **FF** in the mixture (Figure 6c).

## Conclusions

We have developed a new strategy for the precise recognition of unprotected oligopeptides using a synthetic receptor in water. The receptor provides a virtually closed cavity, encircled spherically by multiple polyaromatic panels, in contrast to previous tubular, clip-like, and cage-shaped receptors with aliphatic/small aromatic open cavities. NMR/MS-based, competitive binding experiments revealed that a phenylalanine dipeptide is exclusively bound by the cavity, even from mixtures with aliphatic/aromatic amino acids and oligopeptides (e.g., leucine–phenylalanine, tyrosine–phenylalanine, tryptophan–tryptophan, and phenylalanine trimer) in water, with the small exception of dipeptides with a tryptophan and phenylalanine residue. The quantitative experimental data such as the binding constant and thermodynamic parameters were obtained by ITC analysis. The observed unprecedented high selectivity is derived from size and shape host–guest complementarity as well as multiple host–guest interactions (i.e., hydrogen-bonding and CH−π interactions), enhanced by the closed polyaromatic cavity. In addition, the highly selective and sensitive detection of the dipeptide was achieved by the simple addition of the present receptor and a fluorescent dye.

For different selectivities, we hope that the coordination-based *closed cavity strategy* can be widely applied for not only similar capsules, bearing other polyaromatic panels (e.g., naphthalene and acridinium rings)^24^ and spacers (e.g., pyridine and naphthalene rings)^25^ but also various capsules with closed large cavities, reported by other groups.^26^ Further investigations of the present cavity-directed host–guest system will lead to the development of made-to-order recognition tools for complex biomolecules with high specificity in water.

## Methods

The following analytical instruments and software were used in this study. NMR: Bruker AVANCE III HD 500 (500 MHz; TMS (δ = 0.00 ppm) in CDCl_3_ was used as an external standard for host–guest studies in D_2_O), ESI-TOF MS: Bruker micrOTOF II, UV–visible: JASCO V-670DS, FT-IR: SHIMADZU IRSprit, ITC: MicroCal system, VP-ITC model. Peptide synthesizer: Gyros Protein Technologies, PurePep Chorus. PM6 and DFT calculations: Gaussian 16 program (Rev. C.01) package. Molecular mechanics calculation: Forcite module, BIOVIA Materials Studio 2020, version 20.1.0.5 (Dassault Systèmes Co.).

## Supporting Information Available

Experimental procedures and NMR, MS, IR, ITC, UV, fluorescence, and calculation data (PDF)
