# Supplementary material for: A Closed Cavity Strategy for Selective Dipeptide Binding by a Polyaromatic Receptor in Water
Source: JACS Au. 2023 Oct 2;3(10):2905–11. doi: 10.1021/jacsau.3c00484 (PMC10598568; doi:10.1021/jacsau.3c00484)
Supplement: Supplementary file 1 — au3c00484_si_001.pdf [file au3c00484_si_001.pdf]

Supporting Information

A Closed Cavity Strategy for Selective Dipeptide Binding by a Polyaromatic Receptor in Water

Mayu Shuto, Ryuki Sumida, Mana Yuasa, Tomohisa Sawada, and Michito Yoshizawa*

Laboratory for Chemistry and Life Science, Institute of Innovative Research, Tokyo Institute of Technology, 4259 Nagatsuta, Midori-ku, Yokohama 226-8503, Japan
E-mail: yoshizawa.m.ac@m.titech.ac.jp

Contents

- Materials and methods, and Reference
- Optimized structures and energies of **1•F**, **1•(F)**$_2$, **1•FF**, and **1•FFF** (DFT calculation)
- Synthesis of **FFBn**, **FFF**, **FF-Me**, and **FF-3Me** (^{1}H NMR and ESI-TOF MS spectra)
- Formation of **1•FF** (^{1}H NMR, FT-IR, UV-visible, and ESI-TOF MS spectra)
- Competitive binding of **F**, **FF**, and **FFF** by **1** (^{1}H NMR and ESI-TOF MS spectra)
- Formation of **1•FF-Me** and **1•FF-3Me** (^{1}H NMR, FT-IR, and ESI-TOF MS spectra)
- Competitive binding of **FF** and **CE** or **Asp** by **1** (^{1}H NMR and ESI-TOF MS spectra)
- Thermodynamic studies of **1•FF**, **1•FF-Me**, and **1•FF-3Me** (ITC data)
- Host-guest interactions **of 1•FF** (DFT calculation)
- Competitive binding of **FF**, **LF**, and **LL** by **1** (^{1}H NMR and ESI-TOF MS spectra)
- Competitive binding of **FF** and **AAA** or **AAAA** by **1** (^{1}H NMR spectra)
- Synthesis of **WW** and **WF** (^{1}H NMR and ESI-TOF MS spectra)
- Formation of **1•WF** and **1•FW** (^{1}H NMR and ESI-TOF MS spectra)
- Competitive binding of **FF**, **WF**, and **WW** by **1** (^{1}H NMR and ESI-TOF MS spectra)
- Competitive binding of **FF** and **FW** by **1** (^{1}H NMR and ESI-TOF MS spectra)
- Optimized structures and energies of **1•WF** and **1•WW** (DFT calculation)
- Formation of **1•YF** and **1•FY** (^{1}H NMR and ESI-TOF MS spectra)
- Competitive binding of **FF** and **YF** or **FY** by **1** (^{1}H NMR and ESI-TOF MS spectra)
- Thermodynamic studies of **1•WF** (ITC data)
- Fluorescent detection of **FF** using **1** and **C153** (Fluorescence and ^{1}H NMR spectra**)**
- Cartesian coordinates of **F**, **FF**, **FFF**, **1**, **1•F**, **1•(F)**$_2$, **1•FF**, **1•FFF**, **WF**, **WW**, **1•WF**, and **1•WW**.

Materials and methods

NMR: Bruker AVANCE III HD 500 (500 MHz; TMS (δ = 0.00 ppm) in $CDCl_3$ was used as an external standard for host-guest studies in D_2O), ESI-TOF MS: Bruker micrOTOF II, UV-visible: JASCO V-670DS, FT-IR: SHIMADZU IRSprit, ITC: MicroCal system, VP-ITC model, Peptide synthesizer: Gyros Protein Technologies AB, PurePep Chorus.

Solvents, reagents, and dipeptides (**FF**, **LF**, **LL**, and **YF**): TCI Co., Ltd., FUJIFILM Wako Chemical Co., Kanto Chemical Co., Inc., Sigma-Aldrich Co., BLD Pharm, and Cambridge Isotope Laboratories, Inc. Ultra-pure water (Milli-Q) was used for the ITC analysis. Receptor **1** was synthesized according to a previously reported procedure.[S1] **AAA**, **AAAA**, **FW**, and **FY** were synthesized by a peptide synthesizer.

Calculations

PM6 and DFT calculations: Gaussian 16 program (Rev. C.01) package, Molecular mechanics calculation: Forcite module, BIOVIA Materials Studio 2020, version 20.1.0.5 (Dassault Systèmes Co.).

References

[S1] a) N. Kishi, Z. Li, K. Yoza, M. Akita, M. Yoshizawa, *J. Am. Chem. Soc.* **2011**, *133*, 11438–11441; b) M. Yamashina, Y. Sei, M. Akita, M. Yoshizawa, *Nat. Commun.* **2014**, *5*, 4662.

[S2] H. Skaat, R. Chen, I. Grinberg, S. Margel, *Biomacromolecules* **2012**, *13*, 9, 2662–2670.

[S3] A. Louis, Carpino, X. Jusong, A. El-Faham, *J. Org. Chem.* **2004**, *69*, 54–61.

[S4] N. Bhandaru, G. Kaur, A. Panjlab, S. Verma, *Nanoscale* **2021**, *13*, 8884–8892.

[S5] A. Sahnawaz, S. Kandan, P. Bapan, M. Kallol, D. Debapratim, *Langmuir* **2018**, *34,* 8355–8364.

[S6] S. Ghosh, M. Reches, E. Gazit, S. Verma, *Angew. Chem. Int. Ed.* **2007**, *46*, 2002–2004.

[S7] Y. Liu, J. M. Eckenrode, Y. Zhang, J. Zhang, R. C. Hayden, A. Kyomuhangi, L. V. Ponomareva, Z. Cui, J. Rohr, O. V. Tsodikov, G. Steven, V. Lanen, K. A. Shaaban, M. Leggas, J. S. Thorson, *J. Med. Chem.* **2020**, *63*, 14067–14086.

Optimized structures and energies of 1•F, 1•(F)2, 1•FF, and 1•FFF MS175

The geometry optimizations were performed with PM6 calculation. On the basis of the crystal structure of receptor **1**,[S1] randomly oriented guest **F**, 2•**F**, **FF**, or **FFF** within receptor **1** (R = OCH_3) in several initial structures converged to a single host-guest structure. Energy changes ($\Delta E_{H•G} = E_{H•G} - (E_H + E_G)$ kJ/mol) of receptor **1** before and after the binding of **G** (= **F**, 2•**F**, **FF**, and **FFF**) were estimated by DFT calculation (CAM-B3LYP-GD3BJ /LanL2DZ (Pt), 6-31G(d,p) (others), PCM (H_2O) level of theory). The geometry optimizations of **F**, **FF**, and **FFF** were performed in the same way.

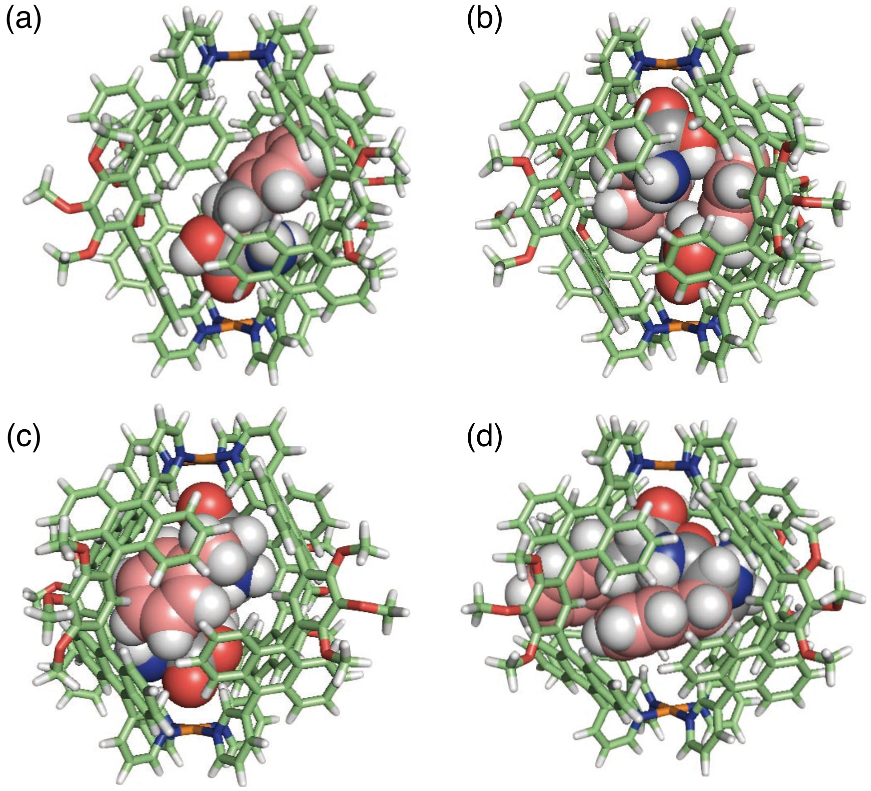

Figure S1a. Optimized structures (PM6 calculations) of (a) **1•F**, (b) **1•(F)**$_2$, (c) **1•FF**, and (d) **1•FFF**.

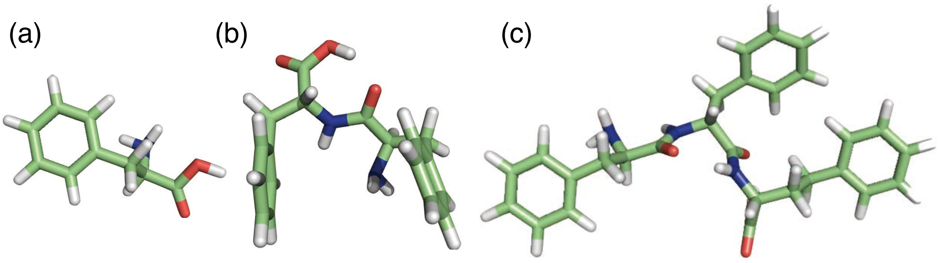

Figure S1b. Optimized structures (PM6 calculations) of (a) **F**, (b) **FF**, and (c) **FFF**.

Table S1. Energy changes (kJ/mol) of receptor **1** before/after the binding of **1•F**, 2•**F**, **FF**, or **FFF** (DFT calculations).

G	E_H	E_G	$E_{H·G}$	$\Delta E_{H·G}$
**F**	–23157673.7	–1455992.5	–24613745.3	–79.2
2•**F**	–23157673.7	–2911985.0	–26069808.0	–149.3
**FF**	–23157673.7	–2711447.9	–25869310.1	–188.5
**FFF**	–23157673.7	–4070036.4	–27227766.8	–56.8

Synthesis of FFBn MS078, 079

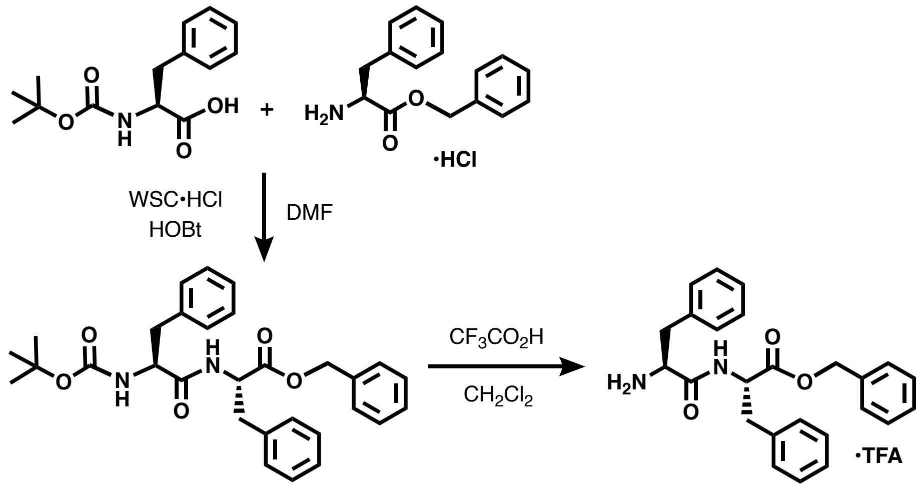

L-Phenylalanine benzyl ester hydrochloride (1.00 g, 3.43 mmol), *N*-(*tert*-butoxycarbonyl)-L-phenylalanine (0.95 g, 3.60 mmol), 1-hydroxy-1*H*-benzotriazole, monohydrate (HOBt, 0.58 g, 3.77 mmol), 1-ethyl-3-(3-dimethylaminopropyl)-carbodiimide, hydrochloride (WSC•HCl, 0.79 g, 4.11 mmol), and DMF (40 mL) were added to a 25 mL glass flask. The reaction mixture was stirred at r.t. overnight. The resulting mixture was diluted with EtOAc (80 mL), washed with 1 M HCl aq. (20 mL), 10% $NaHCO_3$ aq. (20 mL), and brine (20 mL), and dried over $MgSO_4$. After the filteration and removal of the solvent, **BocFFBn** was obtained as a white solid (1.10 g, 2.20 mmol; 64%).[S3] **BocFFBn** (0.20 g, 0.40 mmol) and CH_2Cl_2 (3.0 mL) were added to a 25 mL glass flask. TFA (0.3 mL, 4.0 mmol) was added dropwise over a period of 10 min to the solution at 0 °C. The mixture was stirred at r.t. for 2 h. After the removal of the solvent at 40 °C and washing with diethyl ether, **FFBn** was obtained as a white solid (0.19 g, 0.38 mmol; 94%).[S4]

FFBn: ^{1}H NMR (400 MHz, D_2O, r.t.): δ 7.42-7.18 (m, 15H), 4.18 (t, J = 7.1 Hz, 1H), 3.34 (br, 2H), 3.08 (m, 4H). ESI-TOF MS (CH_3OH): *m/z* Calcd. for $C_{25}H_{26}N_2O_3$ 403.2 [**M** – TFA + H^+]$^+$, Found 403.1.

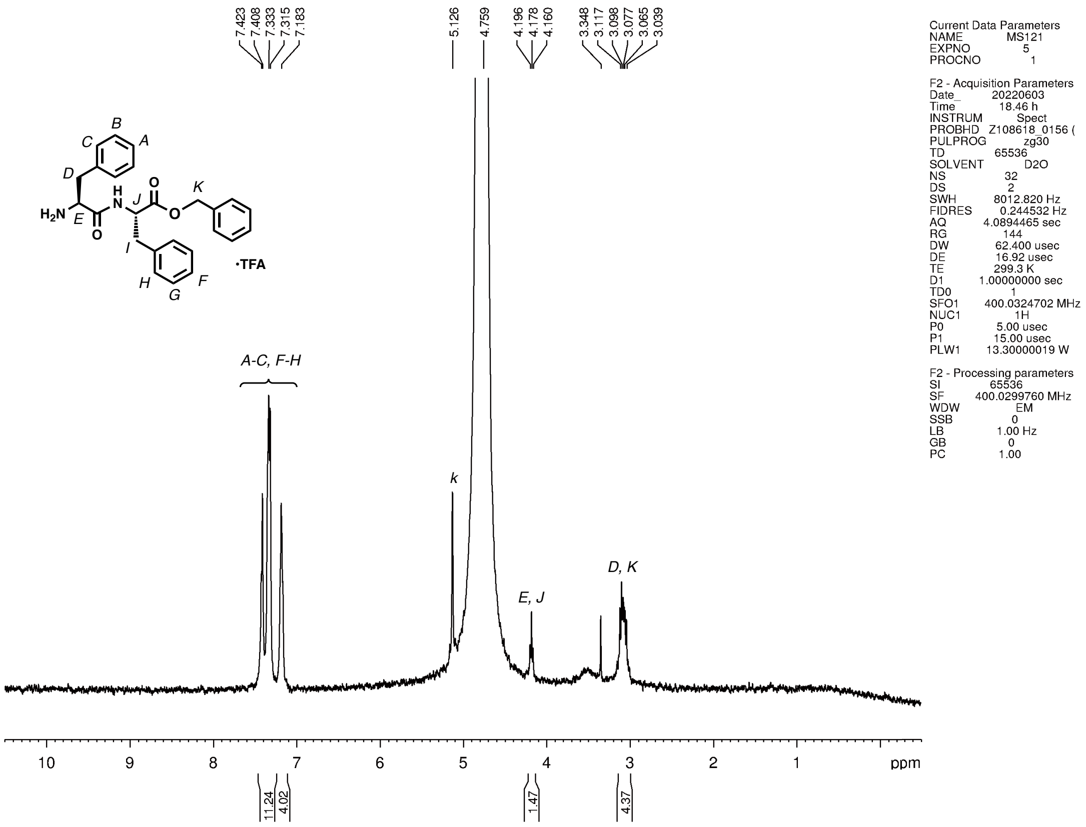

Figure S2a. ^{1}H NMR spectrum (400 MHz, D_2O, r.t.) of **FFBn**.

Analysis Info

Analysis Name	D:\Data\akita\21shuto\MS079\Acq000001.d	Acquisition Date	2021/12/16 12:56:06
Method	Pd Kusaba01.m	Operator	BDAL@DE
Sample Name	ms079_pHEpHEomE	Instrument	micrOTOF 213750.10321
Comment			

Acquisition Parameter

Source Type	ESI	Ion Polarity	Positive	Set Nebulizer	3.0 Bar
Focus	Not active			Set Dry Heater	30 • ‹C
Scan Begin	50 m/z	Set Capillary	4500 V	Set Dry Gas	6.0 l/min
Scan End	3000 m/z	Set End Plate Offset	-500 V	Set Divert Valve	Waste

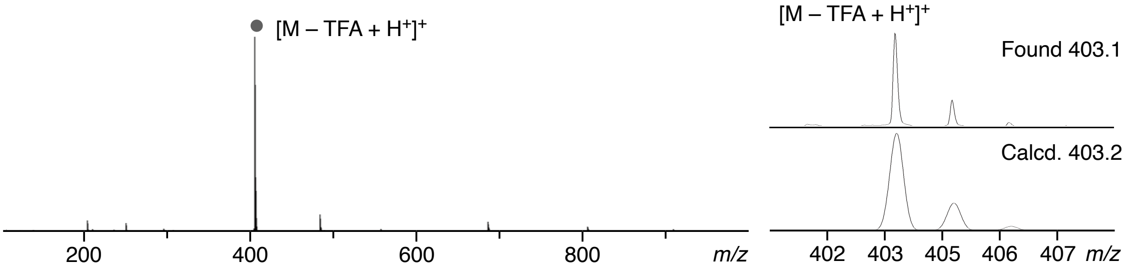

Figure S2b. ESI-TOF MS spectrum (CH_3OH) of **FFBn**.

Synthesis of FFF MS080, 083, 084

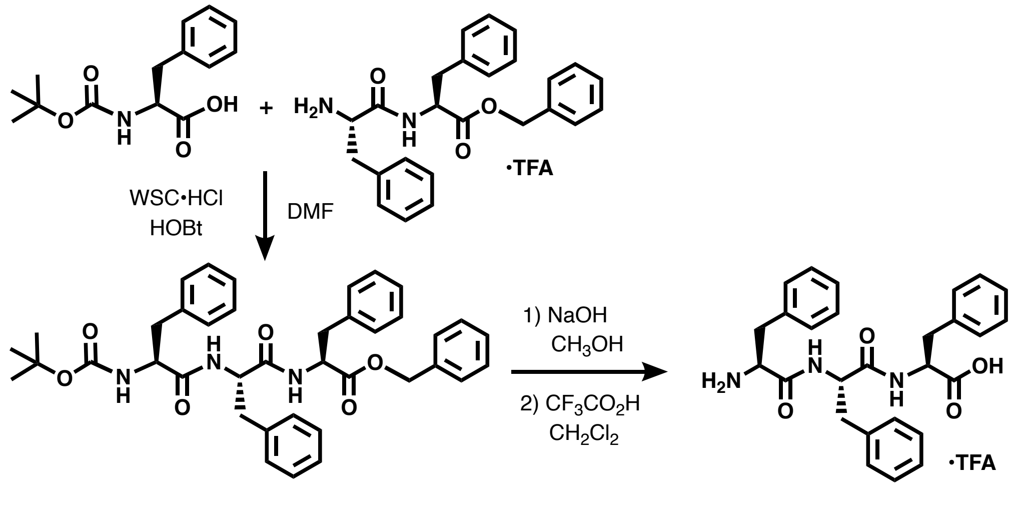

FFBn (0.10 g, 0.26 mmol), *N*-(*tert*-butoxycarbonyl)-L-phenylalanine (0.06 g, 0.21 mmol), HOBt (0.03 g, 0.24 mmol), WSC•HCl (0.04 g, 0.22 mmol), and DMF (2 mL) were added to a 25 mL glass flask. The reaction mixture was stirred at r.t. overnight. The resulting mixture was diluted with EtOAc (10 mL), washed with 1 M HCl (5 mL), 10%$NaHCO_3$ (5 mL), brine (5 mL), and dried over $MgSO_4$. After the filteration and removal of the solvent, **BocFFFBn** was obtained as a white solid (0.13 g, 0.21 mmol; quant.). **BocFFFBn** (20.9 mg, 0.03 mmol), methanol (1.5 mL), and NaOH (3.1 mg, 0.07 mmol) was added to a 25 mL glass flask. The reaction mixture was stirred at r.t. overnight. After the evaporation of the solvent, 5% HCl was added to the solution (until pH 2) to yield a white solid. The solid was extracted with CH_2Cl_2. The resultant solution was dried over Na_2SO_4, filtrated, and concentrated under vacuum to give **BocFFF** as a white solid (19.1 mg, 0.03 mmol; quant.). **BocFFF** (19.1 mg, 0.03 mmol) and CH_2Cl_2 (1.0 mL) were added to a 25 mL glass flask. TFA (0.02 mL, 0.32 mmol) was added dropwise over a period of 10 min to the solution at 0 ºC. The mixture was stirred at r.t. for 2 h. After the removal of the solvent at 40 ºC and washing with diethyl ether, **FFF** was obtained as a white solid (5.9 mg, 12.8 μmol; 40%).[S5]

FFF: ^1H NMR (400 MHz, D_2O, r.t.): δ 2.88-3.16 (m, 6H), 4.12 (m, 1H), 4.37 (m, 1H), 4.62 (m, 1H), 7.34-7.16 (m, 15H). ESI-TOF MS (CH_3OH): *m*/*z* Calcd. For $C_{27}H_{29}N_3O_4$ 460.2 $[M + H^+]^+$, Found 460.1.

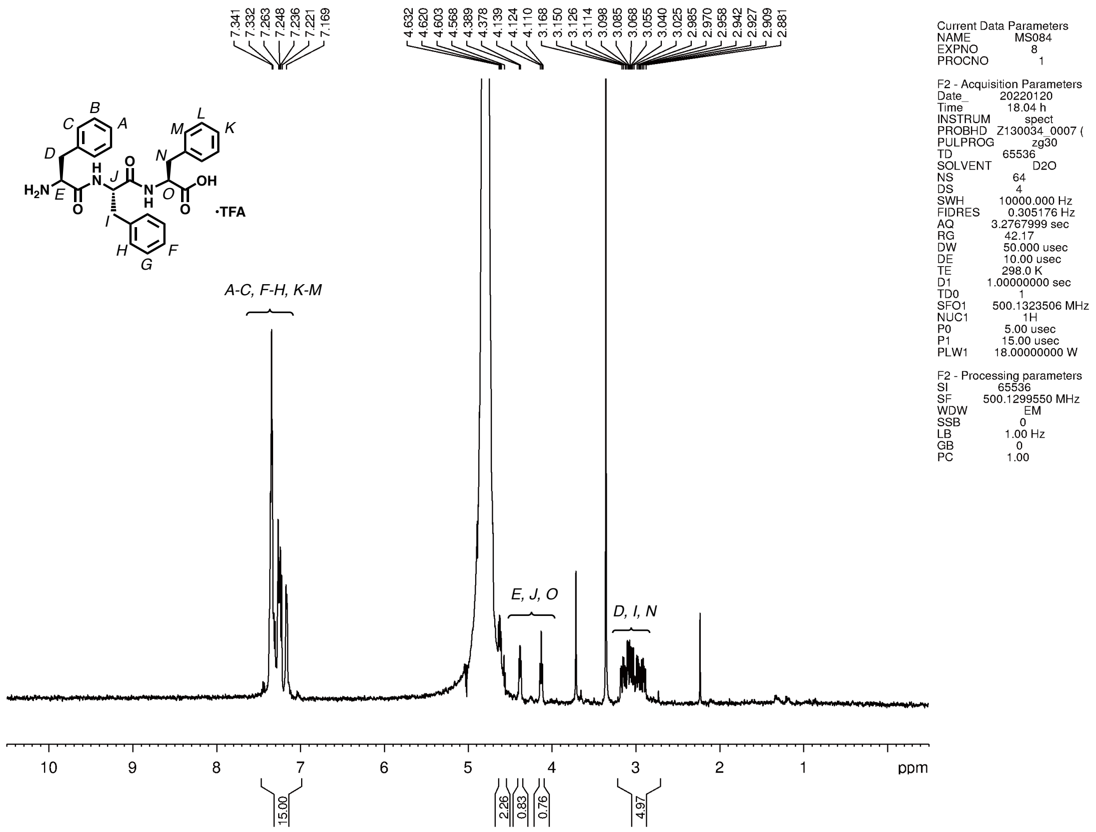

Figure S3a. ^{1}H NMR spectrum (400 MHz, D_2O, r.t.) of **FFF**.

Analysis Info

Analysis Name	D:\Data\akita\21shuto\MS084\Acq000001.d	Acquisition Date	2022/01/18 17:15:07
Method	Pd Kusaba01.m	Operator	BDAL@DE
Sample Name	MS084_PhePhePhe_MeOH	Instrument	micrOTOF 213750.10321
Comment			

Acquisition Parameter

Source Type	ESI	Ion Polarity	Positive	Set Nebulizer	3.0 Bar
Focus	Not active			Set Dry Heater	30 • ‹C
Scan Begin	50 m/z	Set Capillary	4500 V	Set Dry Gas	6.0 l/min
Scan End	3000 m/z	Set End Plate Offset	-500 V	Set Divert Valve	Waste

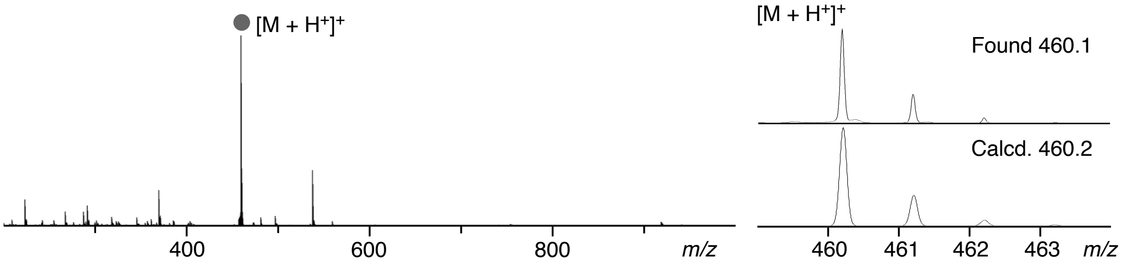

Figure S3b. ESI-TOF MS spectrum (CH_3OH) of **FFF**.

Synthesis of FF-Me   MS077

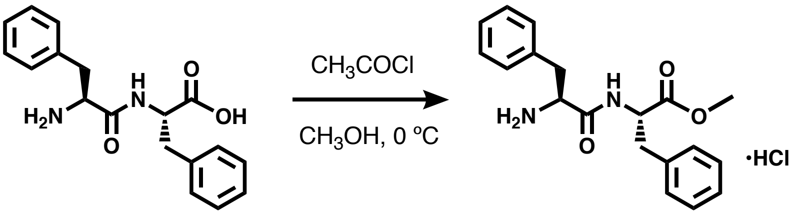

L-Phenylalanyl-L-phenylalanine (5.0 mg, 0.16 μmol) and anhydrous methanol (0.5 mL) were added to a 25 mL glass flask. Acetyl chloride (28 μL, 0.38 mmol) was then added dropwise to the flask at 0 °C. The reaction mixture was stirred at 0 °C for 2 h and then at r.t. overnight. After the removal of the solvent and unreacted substrates under reduced pressure at 40 °C, **FF-Me** was obtained as a white solid (3.7 mg, 0.11 μmol; 71%).[S2]

^{1}H NMR (400 MHz, CD_3OD, r.t.): δ 2.95-3.04 (m, 3H), 3.17-3.24 (m, 2H), 3.69 (s, 3H), 4.05 (dd, J = 8.3, 5.9 Hz, 1H), 4.73 (dd, J = 8.6, 5.1 Hz, 1H), 7.21-7.36 (m, 11H). ESI-TOF MS (CH_3OH): Calcd. for $C_{19}H_{23}N_2O_3$ 327.1 [M – Cl^-]$^+$, Found 327.1.

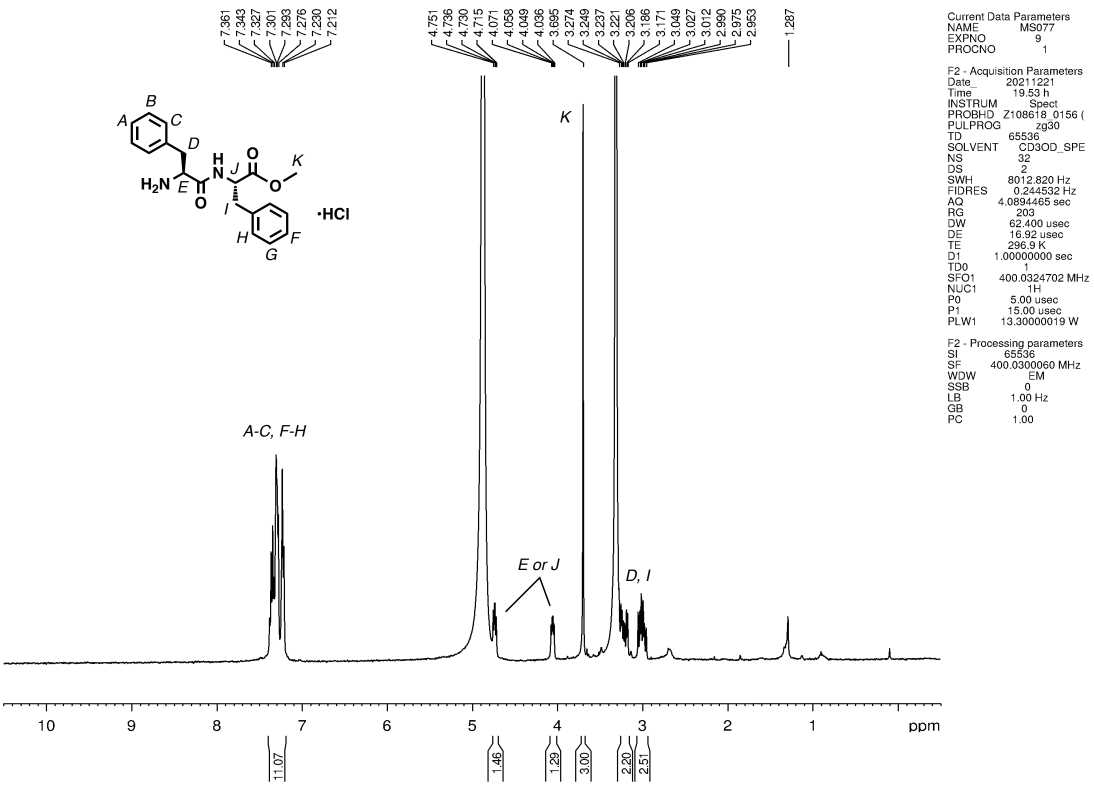

Figure S4a. ^{1}H NMR spectrum (400 MHz, CD_3OD, r.t.) of **FF-Me**.

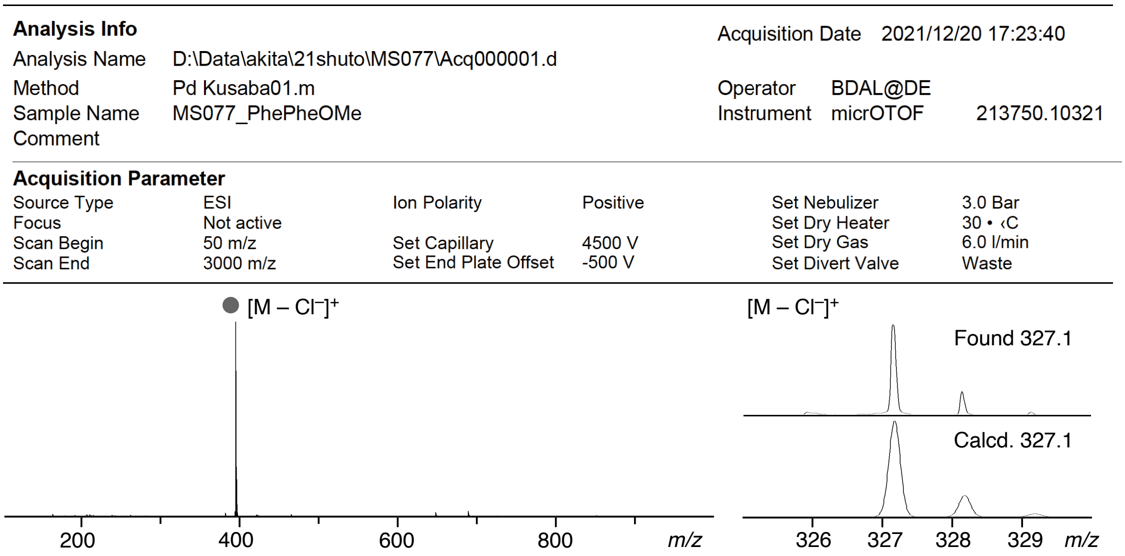

Figure S4b. ESI-TOF MS spectrum (CH_3OH) of **FF-Me**.

Synthesis of FF-3Me MS138

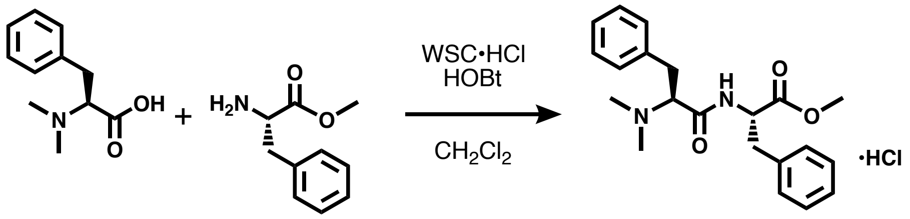

L-Phenylalanine methyl ester hydrochloride (30.4 mg, 0.14 mmol), *N,N*-dimethyl-L-phenylalanine (30.1 mg, 0.16 mmol), HOBt (26.1 mg, 0.17 mmol), WSC•HCl (32.9 mg, 0.17 mmol), triethylamine (39.3 μL, 0.28 mmol), and CH_2Cl_2 (4 mL) were added to a 25 mL glass flask. The reaction mixture was stirred at r.t. overnight. The resulting mixture was diluted with CH_2Cl_2 (10 mL), washed with 1 M HCl aq. (5 mL) and dried over $MgSO_4$. After the filteration and removal of the solvent, **FF-3Me** was obtained as a white solid (38.7 mg, 0.11 mmol; 73%). ^{1}H NMR (400 MHz, D_2O, r.t.): 2.55 (s, 6H), 2.72-2.79 (dd, J = 12.3, 13.4 Hz, 1H), 2.90-2.96 (dd, J = 12.0, 11.8 Hz, 1H), 3.18-3.21 (dd, J = 13.8, 4.24 Hz, 1H), 3.57 (m, 1H), 3.58 (s, 3H), 3.81 (m, 2H), 7.19-7.36 (m, 10H). ESI-TOF MS (CH_3OH): Calcd. for $C_{19}H_{23}N_2O_3$ 355.2 $[M + H^+]^+$, Found 355.1.

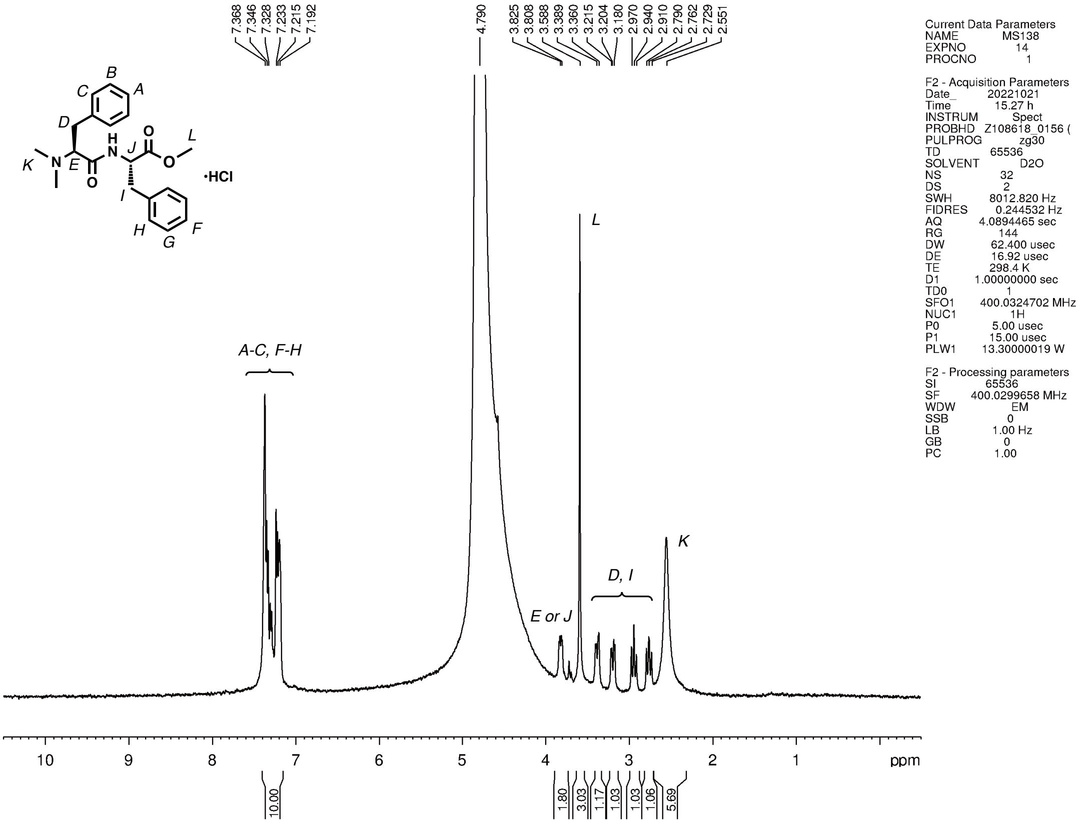

Figure S5a. ^{1}H NMR spectrum (400 MHz, D_2O, r.t.) of **FF-3Me**.

Analysis Info

Analysis Name	D:\Data\akita\21shuto\MS138\acq00000000001.d	Acquisition Date	2022/06/29 13:22:54
Method	Pd Kusaba01.m	Operator	BDAL@DE
Sample Name	MS138_CH3OH	Instrument	micrOTOF 213750.10321
Comment			

Acquisition Parameter

Source Type	ESI	Ion Polarity	Positive	Set Nebulizer	3.0 Bar
Focus	Not active			Set Dry Heater	30 • ‹C
Scan Begin	50 m/z	Set Capillary	4500 V	Set Dry Gas	6.0 l/min
Scan End	3000 m/z	Set End Plate Offset	-500 V	Set Divert Valve	Waste

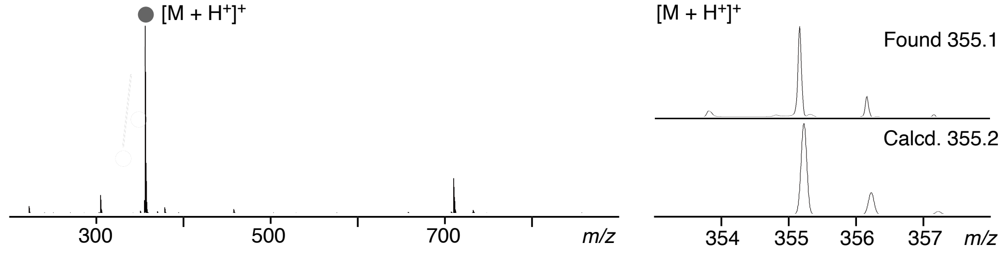

Figure S5b. ESI-TOF MS spectrum (CH_3OH) of **FF-3Me**.

Formation of 1•FF MS058, 294

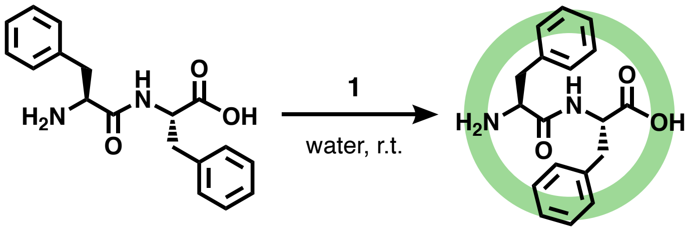

Receptor **1** (1.0 mg, 0.27 μmol) and L-phenylalanyl-L-phenylalanine (**FF**; 0.4 mg, 1.2 μmol) were added to a 2 mL test tube containing D_2O (0.5 mL). The mixture was stirred at r.t. for 30 min. The quantitative formation of 1:1 host-guest complex **1•FF** was confirmed by ^1H NMR, IR, ESI-TOF MS, and ITC analyses.

^1H NMR (500 MHz, D_2O, r.t.): δ –0.98 (br, 0.5H, **FF**), –0.90 (br, 0.5H, **FF**), –0.80 (br, 0.5H, **FF**), –0.76 (br, 0.5H, **FF**), –0.41 (br, 0.5H, **FF**), –0.20 (m, 0.5H, **FF**), –0.03 (br, 0.5H, **FF**), 0.26 (br, 0.5H, **FF**), 0.31 (br, 0.5H, **FF**), 0.41 (br, 0.5H, **FF**), 0.59 (br, 0.5H, **FF**), 0.99 (br, 0.5H, **FF**), 2.43-2.47 (br, 24H, $\mathbf{1}_{H_l}$), 3.12 (br, 16H, $\mathbf{1}_{H_k}$), 3.48 (s, 12H, $\mathbf{1}_{H_{l'}}$), 3.94 (m, 16H, $\mathbf{1}_{H_j}$), 4.07 (m, 8H, $\mathbf{1}_{H_{k'}}$), 4.47 (m, 4H, $\mathbf{1}_{H_{j'}}$), 4.62 (m, 4H, $\mathbf{1}_{H_{j''}}$), 6.02 (br, 2H, $\mathbf{1}_{H_a}$), 6.26 (s, 2H, $\mathbf{1}_{H_{a'}}$), 6.47 (s, 4H, $\mathbf{1}_{H_e}$), 6.58 (br, 4H, $\mathbf{1}_{H_d}$), 6.81 (br, 4H, $\mathbf{1}_{H_c}$), 7.03 (dd, *J* = 21, 9.0 Hz, 8H, $\mathbf{1}_{H_{e'}}$), 7.53 (m, 8H, $\mathbf{1}_{H_b}$), 7.69 (m, 8H, $\mathbf{1}_{H_{c'}}$), 7.80 (br, 8H, $\mathbf{1}_{H_{b'}}$), 8.03 (t, *J* = 7.6 Hz, 8H, $\mathbf{1}_{H_h}$), 8.32 (m, 8H, $\mathbf{1}_{H_f}$), 8.56 (m, 8H, $\mathbf{1}_{H_i}$), 9.17 (d, *J* = 5.3 Hz, 4H, $\mathbf{1}_{H_g}$), 9.24 (d, *J* = 5.5 Hz, 4H, $\mathbf{1}_{H_{g'}}$). FT-IR (KBr, cm^{-1}): 1655($\nu_{C=O}$), 1603($\nu_{C=O}$). ESI-TOF MS (H_2O): *m/z* 983.0 [**1•FF** – 4•NO_3^-]$^{4+}$, 1331.7 [**1•FF** – 3•NO_3^-]$^{3+}$.

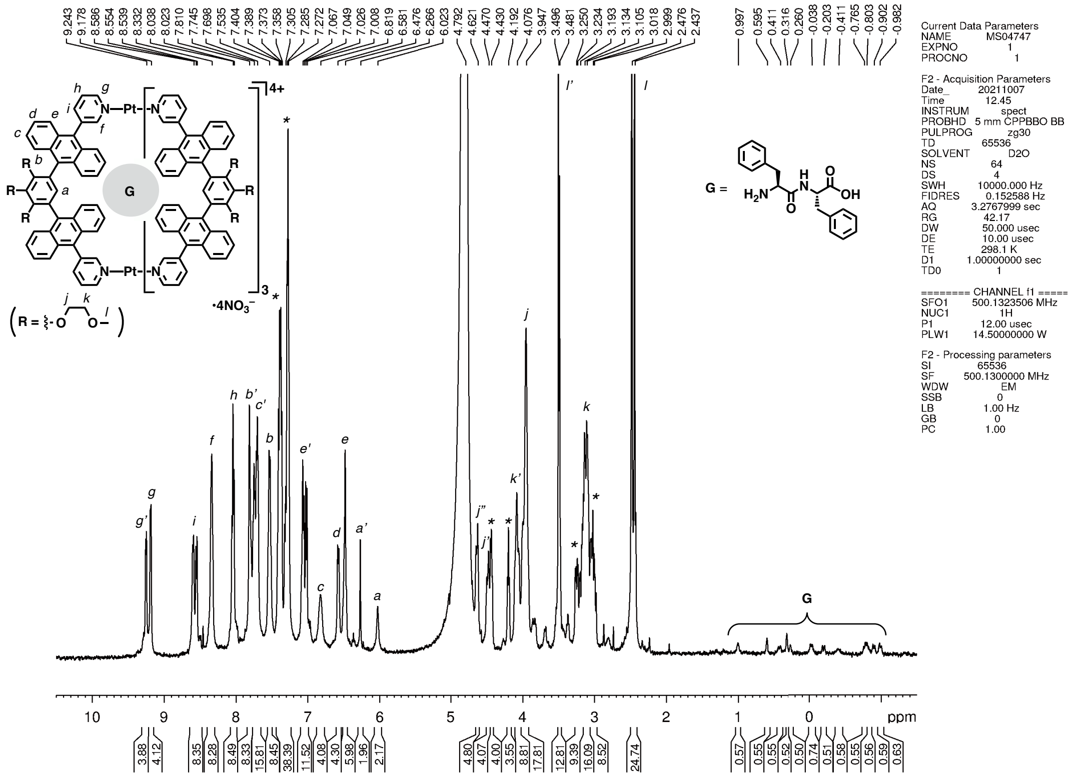

Figure S6a. ^{1}H NMR spectrum (500 MHz, D_2O, r.t.) of **1•FF** (*: free **FF**).

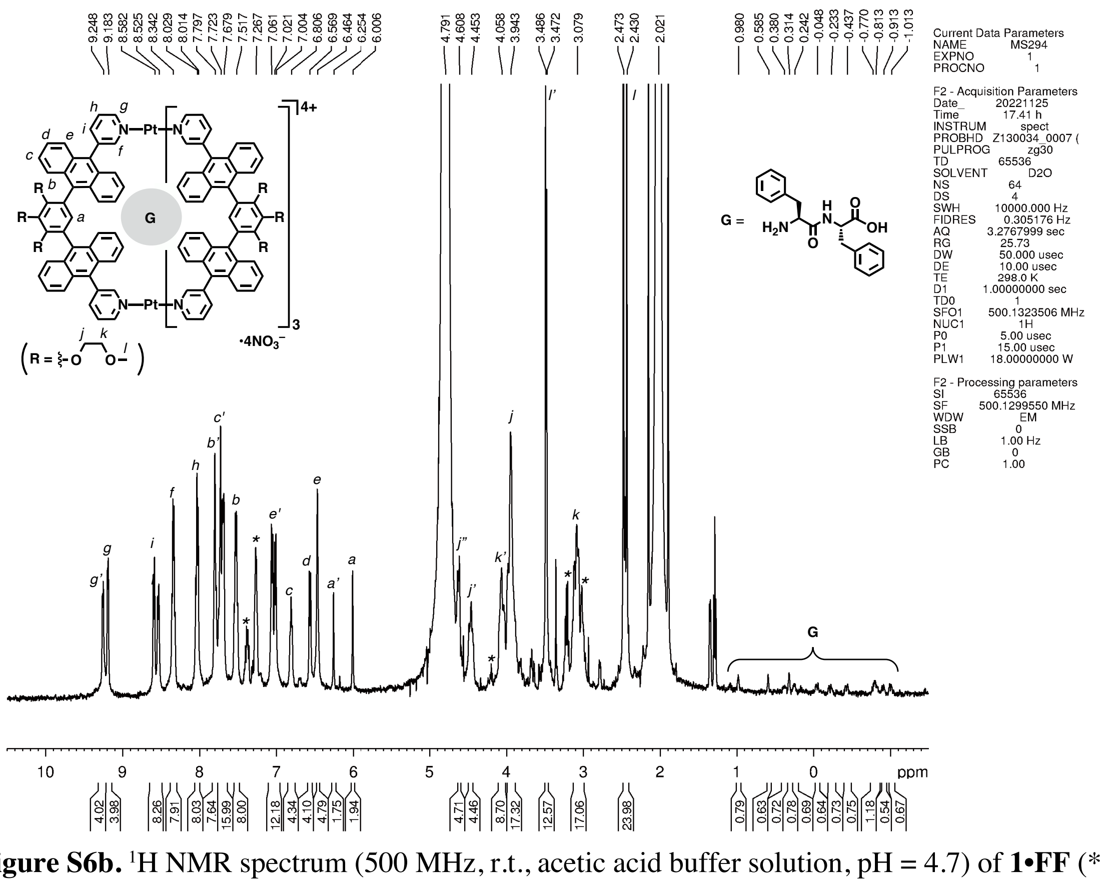

Figure S6b. ^{1}H NMR spectrum (500 MHz, r.t., acetic acid buffer solution, pH = 4.7) of **1•FF** (*: free **FF**).

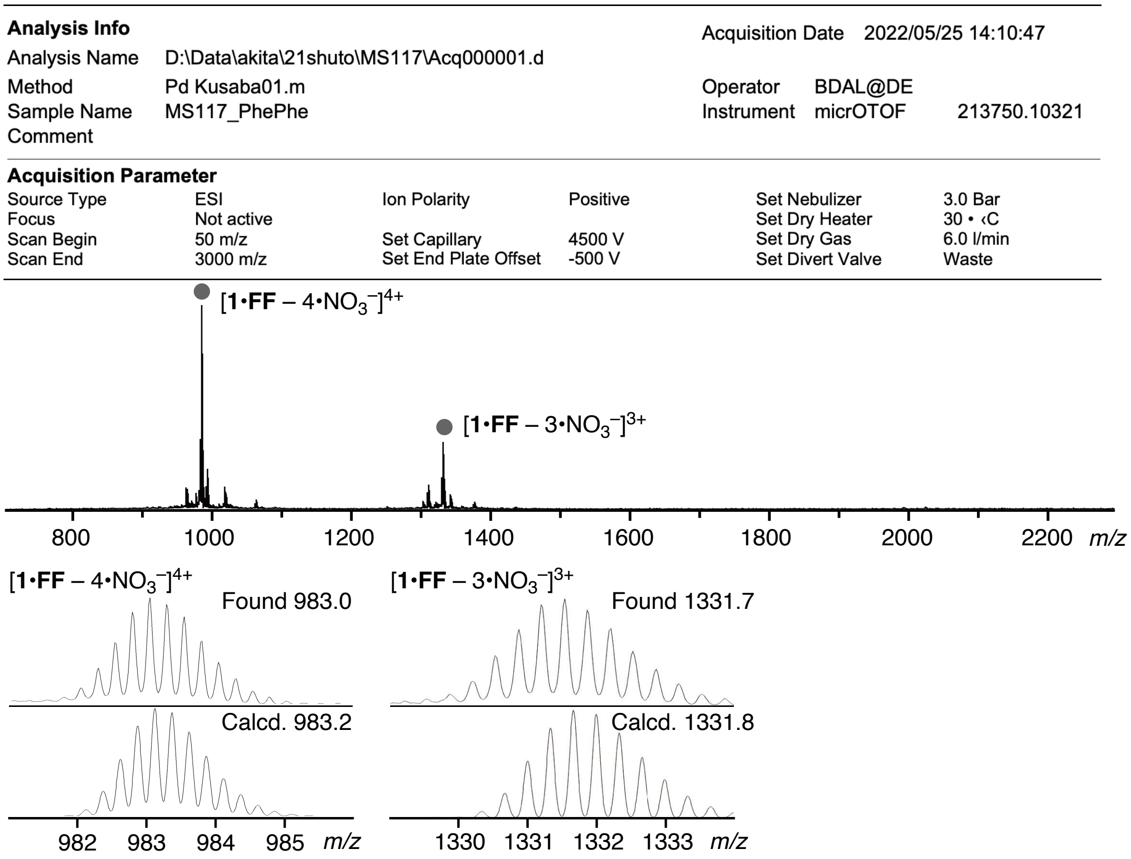

Figure S6c. ESI-TOF MS spectrum (H_2O) of **1•FF**.

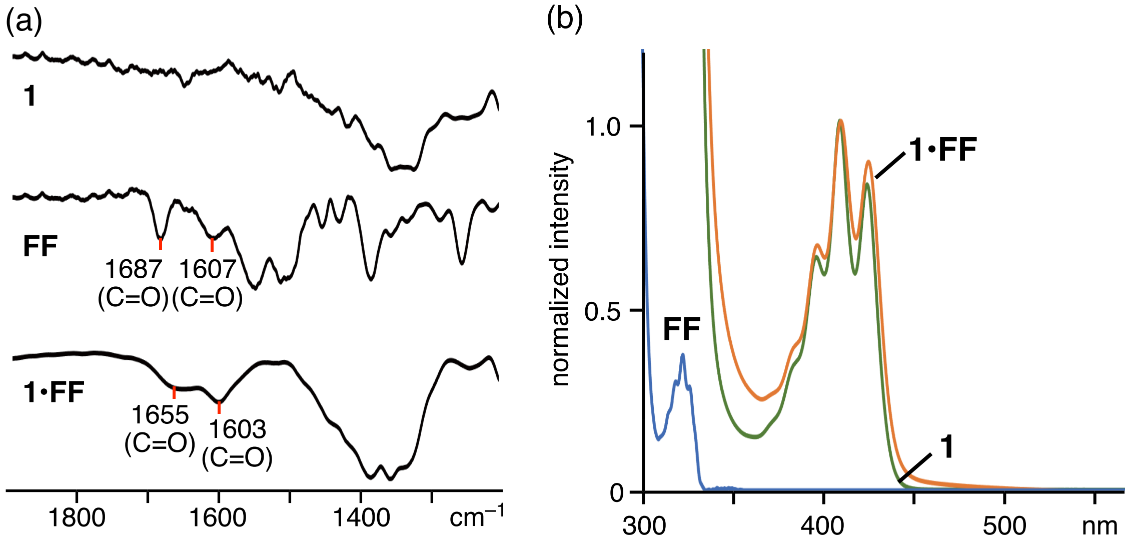

Figure S6d. (a) FT-IR spectra (ATR, r.t.) of **1**, **FF**, and **1•FF**. (b) UV-visible spectra (H_2O, r.t., 0.4 mM based on **1**) of **1**, **FF**, and **1•FF**.

Competitive binding of F, FF, and FFF by 1 MS064, 110, 164, 194

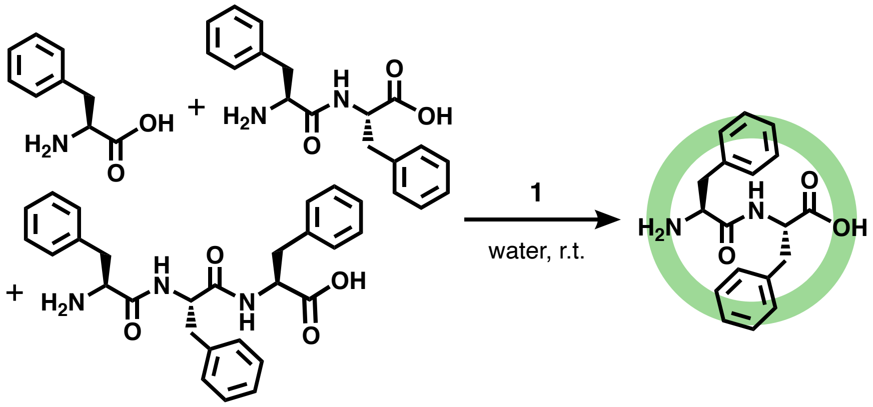

Receptor **1** (0.5 mg, 0.13 μmol), L-phenylalanine (**F**; 0.02 mg, 0.13 μmol), **FF** (0.04 mg, 0.13 μmol), and **FFF** (0.06 mg, 0.13 μmol) were added to a 2 mL test tube containing D_2O (0.5 mL). The mixture was stirred at r.t. for 30 min. The selective formation of 1:1 host-guest complex **1•FF** was confirmed by ^{1}H NMR analyses. After the isolation of the products via salting out by addition of KNO_3 (1.5 mg), the selective formation of **1•FF** was further confirmed by ESI-TOF MS analysis. In the same way, receptor **1** (0.5 mg, 0.13 μmol), **FF** (0.04 mg, 0.13 μmol), **FFF** (0.37 mg, 0.80 μmol), and **F** (0.13 mg, 0.80 μmol) were added to a 2 mL test tube containing D_2O (0.5 mL). The mixture was stirred at r.t. for 30 min. The selective formation of 1:1 host-guest complex **1•FF** was confirmed by ^{1}H NMR and ESI-TOF MS analyses. In the same way, receptor **1** (0.5 mg, 0.13 μmol), **F** (0.03 mg, 0.16 μmol), and **FF** (0.05 mg, 0.16 μmol) were added to a 2 mL test tube containing D_2O (0.5 mL). The mixture was stirred at r.t. for 30 min. The selective formation of 1:1 host-guest complex **1•FF** was confirmed by ^{1}H NMR and ESI-TOF MS analyses.

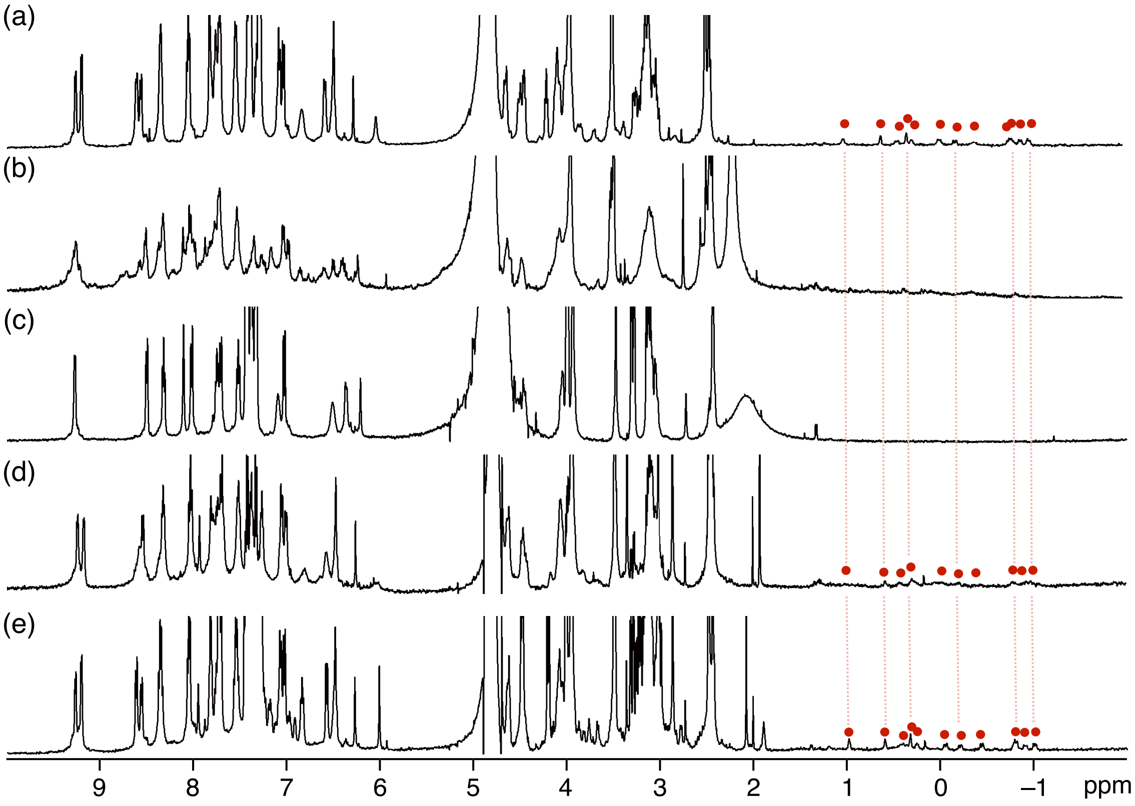

Figure S7a. ^{1}H NMR spectra (500 MHz, D_2O, r.t.) of (a) **1•FF**, (b) **1** + **FFF**, (c) **1** + **F**, (d) products after mixing **F**, **FF**, and **FFF**, and (e) products after mixing **FF**, **F** (5 eq.), and **FFF** (5 eq.) with **1** at r.t. for 30 min.

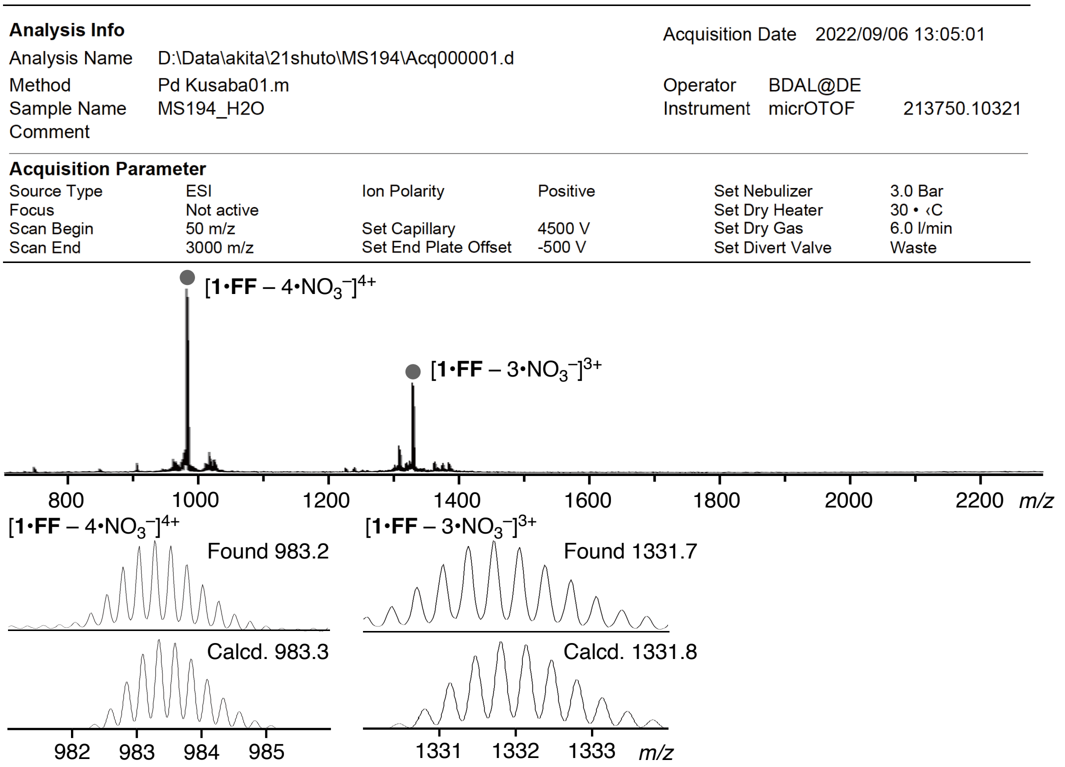

Figure S7b. ESI-TOF MS spectrum (H_2O) of products after mixing **F**, **FF**, and **FFF** with **1** at r.t. for 30 min.

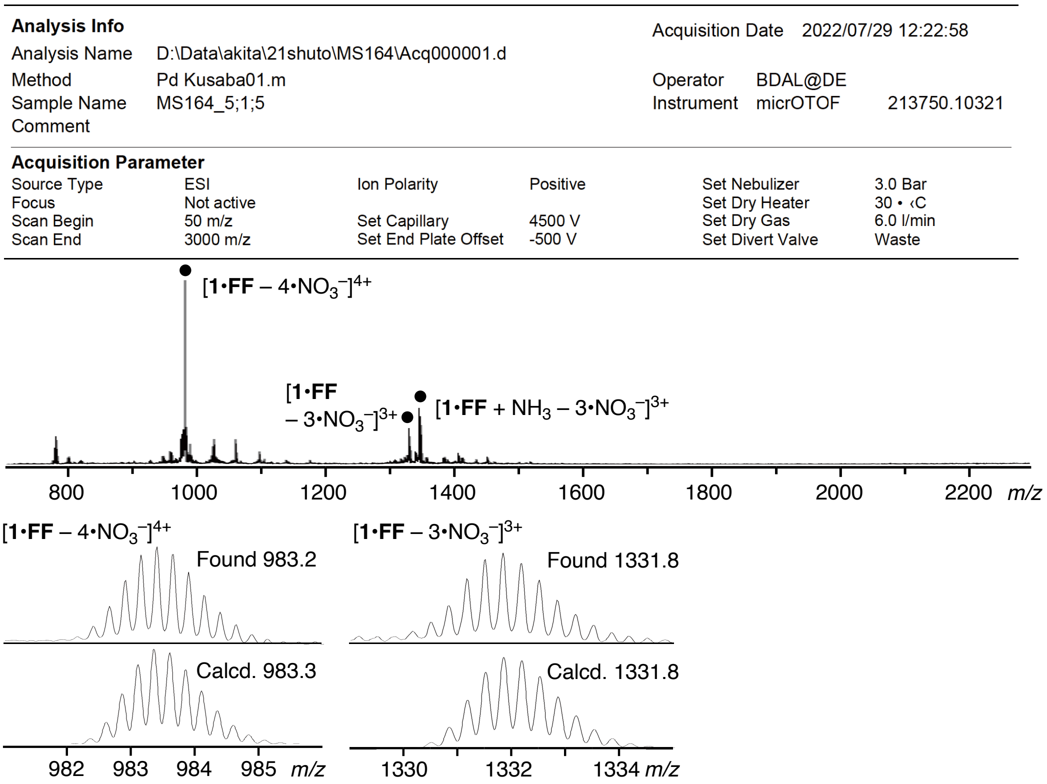

Figure S7c. ESI-TOF MS spectrum (H_2O) of products after mixing **FF**, **F** (5 eq), and **FFF** (5 eq) with **1** at r.t. for 30 min.

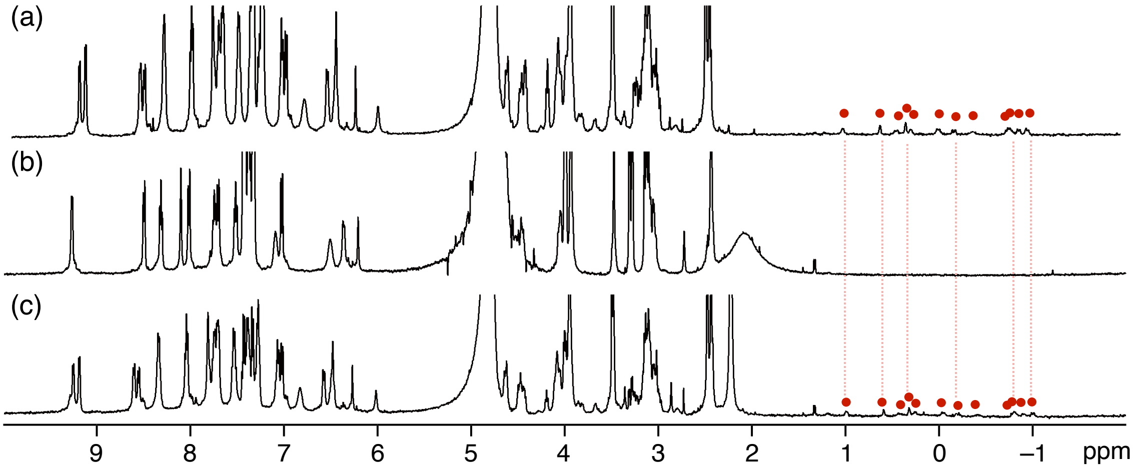

Figure S7d. ^{1}H NMR spectra (500 MHz, D_2O, r.t.) of (a) **1•FF**, (b) **1** + **F**, and (c) products after mixing **FF** and **F** with **1** at r.t. for 30 min.

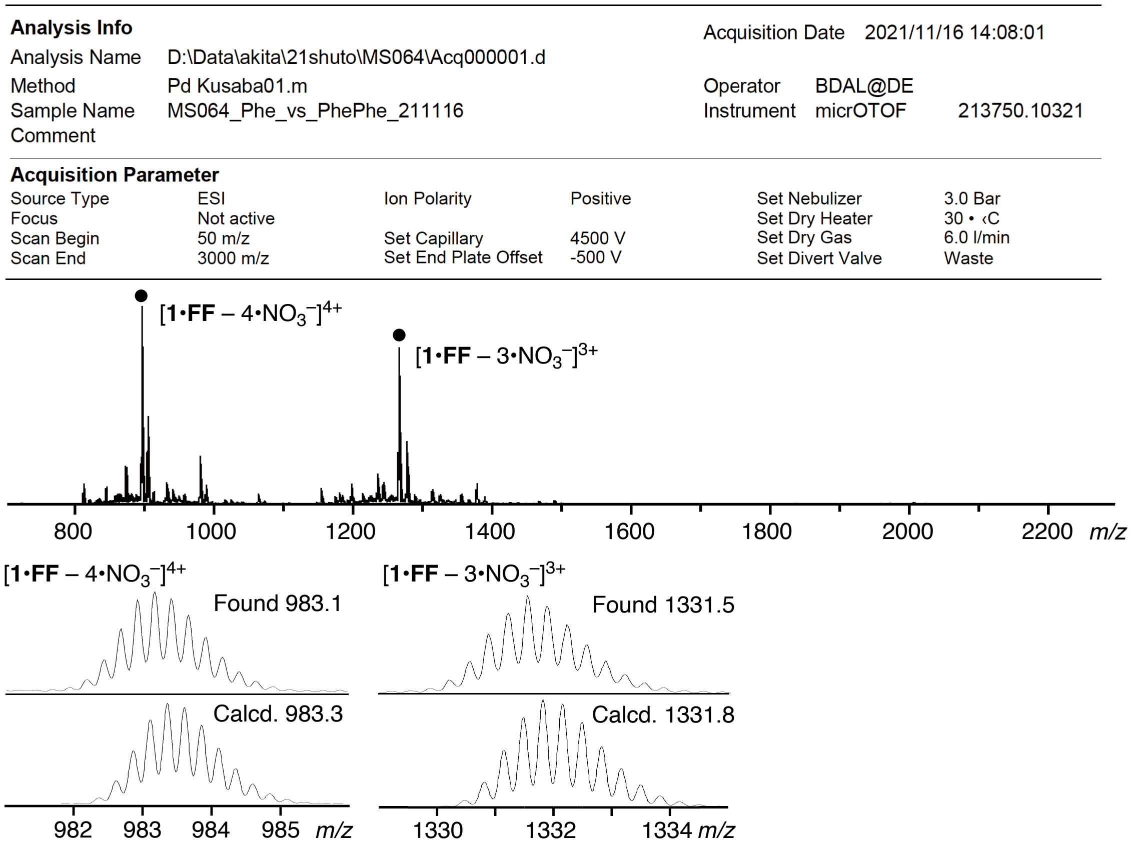

Figure S7e. ESI-TOF MS spectrum (H_2O) of products after mixing **F** and **FF** with **1** at r.t. for 30 min.

Formation of 1•FF-Me MS085

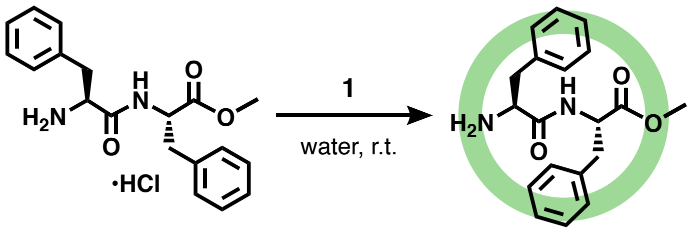

Receptor **1** (0.5 mg, 0.14 µmol) and **FF-Me** (0.05 mg, 0.56 µmol) were added to a 2 mL test tube containing D_2O (0.5 mL). The mixture was stirred at r.t. for 30 min. The quantitative formation of 1:1 host-guest complex **1•FF-Me** was confirmed by ^{1}H NMR, ESI-TOF MS, IR, and ITC analyses.

^{1}H NMR (500 MHz, D_2O, r.t.): δ –0.66 (br, 1H, **FF-Me**), –0.55 (br, 1H, **FF-Me**), –0.33 (s, 3H, **FF-Me**), –0.17 (br, 1H, **FF-Me**), 0.24 (br, 1H, **FF-Me**), 0.92 (br, 1H, **FF-Me**), 1.34 (m, 1H, **FF-Me**), 2.50 (s, 24H, $\mathbf{1}_{H_l}$), 3.07-3.10 (m, 16H, $\mathbf{1}_{H_k}$), 3.49 (s, 12H, $\mathbf{1}_{H_{l'}}$), 3.94 (br, 16H, $\mathbf{1}_{H_j}$), 4.08 (br, 8H, $\mathbf{1}_{H_k}$), 4.47 (m, 4H, $\mathbf{1}_{H_{j'}}$), 4.62 (m, 4H, $\mathbf{1}_{H_{j''}}$), 6.16 (s, 4H, $\mathbf{1}_{H_a}$), 6.49 (d, J = 8.4 Hz, 8H, $\mathbf{1}_{H_e}$), 6.81 (dd, J = 7.2, 7.3 Hz, 8H, $\mathbf{1}_{H_d}$), 7.03 (m, 8H, $\mathbf{1}_{H_{e'}}$), 7.54

(dd, J = 7.3, 7.9 Hz, 8H, $\mathbf{1}_{H_c}$), 7.71 (d, J = 8.6 Hz, 8H, $\mathbf{1}_{H_b}$), 7.80 (br, 8H, $\mathbf{1}_{H_{c'}}$), 8.04 (d, J = 8.6 Hz, 8H, $\mathbf{1}_{H_{b'}}$), 8.36 (dd, J = 6.7, 7.0 Hz, 8H, $\mathbf{1}_{H_h}$), 8.60 (d, J = 7.8 Hz, 8H, $\mathbf{1}_{H_i}$), 9.22 (d, J = 5.4 Hz, 8H, $\mathbf{1}_{H_g}$). FT-IR (ATR, cm^{-1}): 1662 ($\nu_{C=O}$), 1605 ($\nu_{C=O}$). ESI-TOF MS (H_2O): m/z 986.4 [**1•FF-Me** – 4•NO_3^-]$^{4+}$, 1335.8 [**1•FF-Me** – 3•NO_3^-]$^{3+}$.

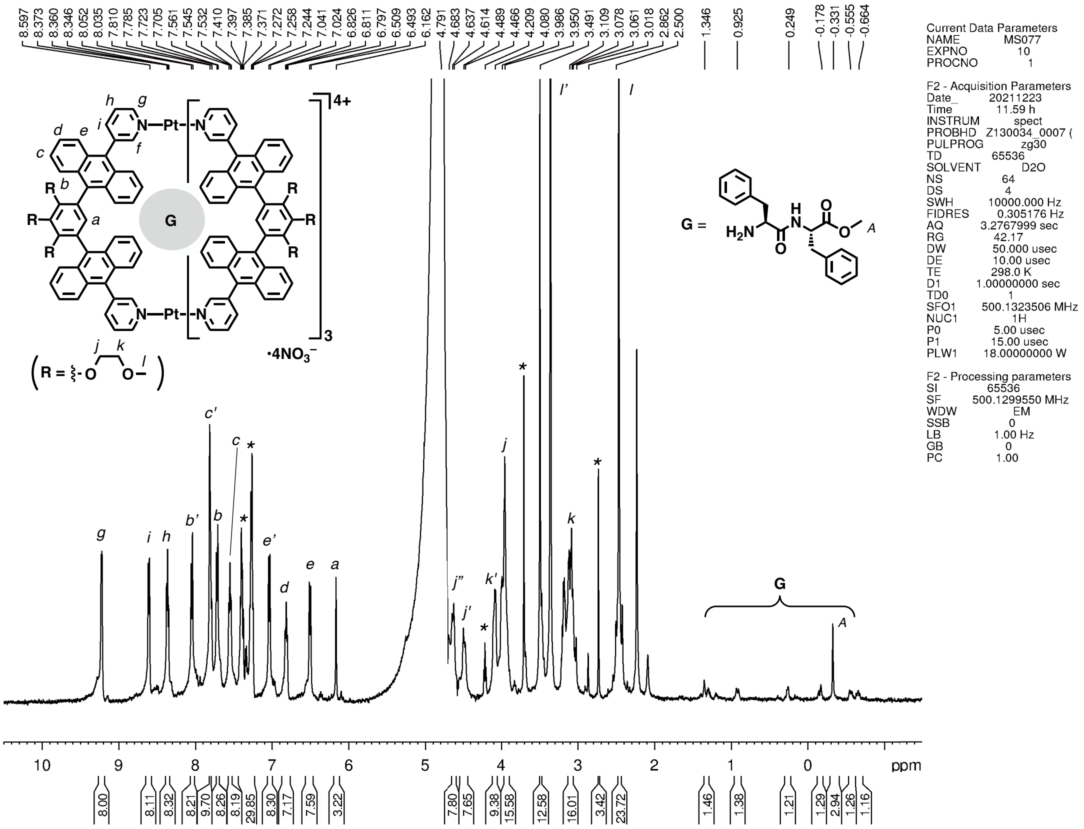

Figure S8a. ^{1}H NMR spectrum (500 MHz, D_2O, r.t.) of **1•FF-Me** (*: free **FF-Me**).

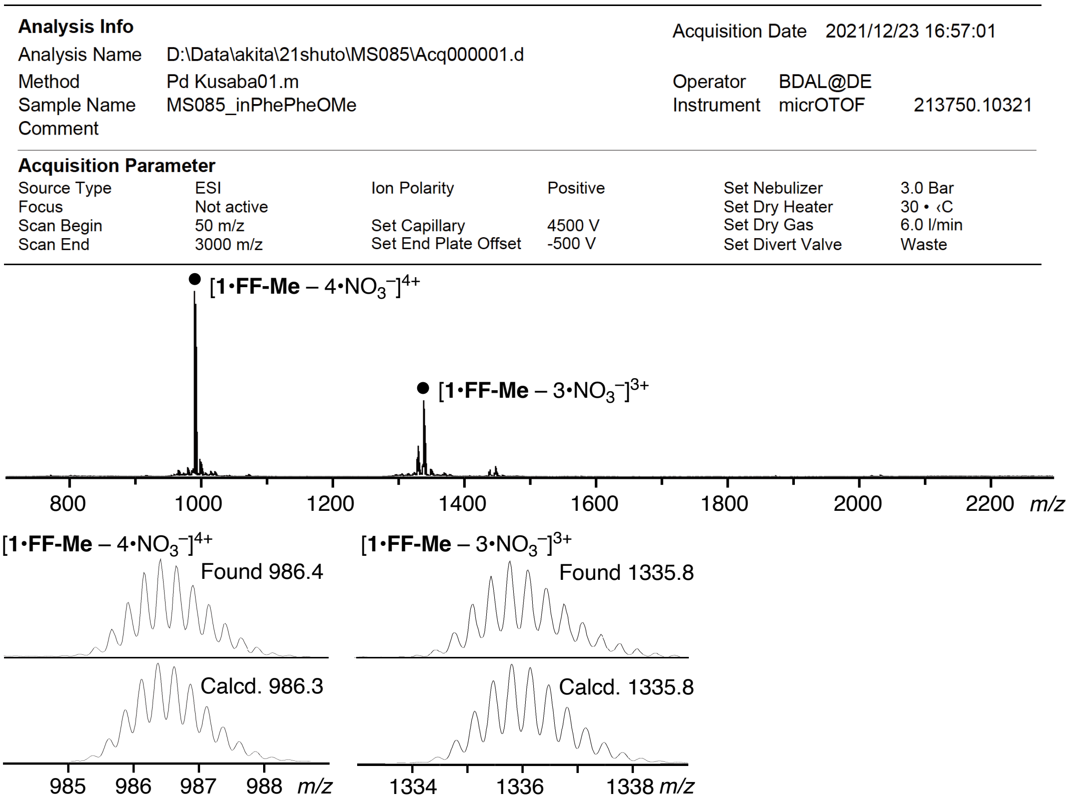

Figure S8b. ESI-TOF MS spectrum (H_2O) of **1•FF-Me**.

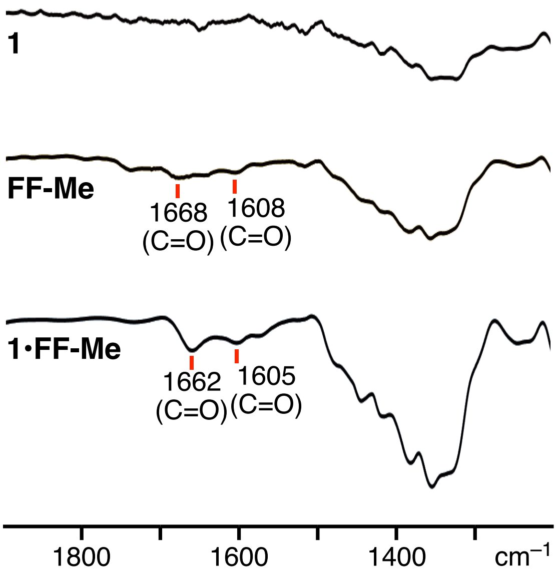

Figure S8c. FT-IR spectra (ATR, r.t.) of **1**, **FF-Me**, and **1•FF-Me**.

Formation of 1•FF-3Me   MS143

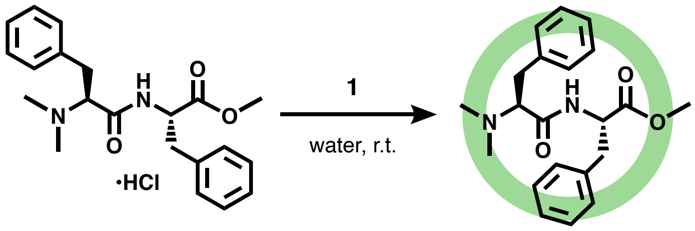

Receptor **1** (0.5 mg, 0.14 μmol) and **FF-3Me** (0.05 mg, 0.14 μmol) were added to a 2 mL test tube containing D_2O (0.5 mL). The mixture was stirred at r.t. for 30 min. The quantitative formation of 1:1 host-guest complex **1•FF-3Me** was confirmed by ^{1}H NMR, ESI-TOF MS, and ITC analyses.

^{1}H NMR (500 MHz, D_2O, r.t.): δ –1.51 (s, 6H, **FF-3Me**), –1.28 (s, 1H, **FF-3Me**), –1.20 (br, 1H, **FF-3Me**), –0.78 (br, 1H, **FF-3Me**), –0.22 (br, 1H, **FF-3Me**), –0.03 (br, 1H, **FF-3Me**), 0.47-0.52 (br, 3H, **FF-3Me**), 1.06 (s, 1H, **FF-3Me**), 2.50 (s, 24H, $\mathbf{1}_{H_l}$), 3.14-3.21 (m, 16H, $\mathbf{1}_{H_k}$), 3.50 (s, 12H, $\mathbf{1}_{H_{l'}}$), 3.95-4.02 (br, 16H, $\mathbf{1}_{H_j}$), 4.08 (br, 8H, $\mathbf{1}_{H_k}$), 4.50 (m, 4H, $\mathbf{1}_{H_{j'}}$), 4.62 (m, 4H, $\mathbf{1}_{H_{j''}}$), 5.59 (s, 3H, $\mathbf{1}_{H_a}$), 6.71 (d, J = 8.5 Hz, 8H, $\mathbf{1}_{H_d}$), 6.85 (m, 1H, $\mathbf{1}_{H_{a'}}$), 6.99 (d, 16H, J = 8.8 Hz, $\mathbf{1}_{H_e}$), 7.19 (br, 4H, $\mathbf{1}_{H_{d'}}$), 7.39 (s, 8H, $\mathbf{1}_{H_f}$), 7.52 (dd, J = 7.3, 7.9 Hz, 8H, $\mathbf{1}_{H_c}$), 7.67 (d, 8H, J = 8.4 Hz, $\mathbf{1}_{H_b}$), 7.78 (br, 8H, $\mathbf{1}_{H_{c'}}$), 7.98 (br, 8H, $\mathbf{1}_{H_{b'}}$), 8.36 (br, 8H, $\mathbf{1}_{H_h}$), 8.66 (d, J = 7.2 Hz, 8H, $\mathbf{1}_{H_i}$), 9.19 (d, J = 6.6 Hz, 8H, $\mathbf{1}_{H_g}$). ESI-TOF MS (H_2O): m/z 993.0 [**1•FF-3Me** – 4•NO_3^-]$^{4+}$, 1344.7 [**1•FF-3Me** – 3•NO_3^-]$^{3+}$.

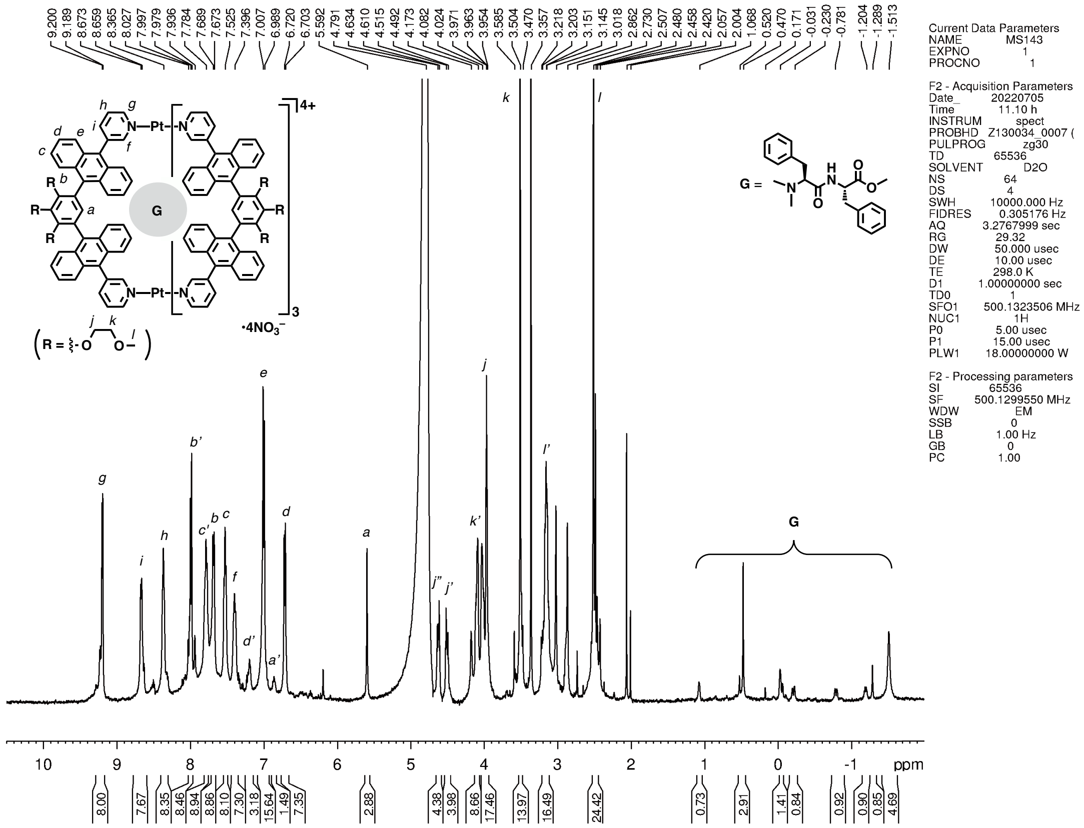

j

Figure S9a. ^{1}H NMR spectrum (500 MHz, D_2O, r.t.) of **1•FF-3Me**.

Analysis Info

Analysis Name	D:\Data\akita\21shuto\MS143\Acq000001.d	Acquisition Date	2022/07/06 13:49:49
Method	Pd Kusaba01.m	Operator	BDAL@DE
Sample Name	MS143	Instrument	micrOTOF 213750.10321
Comment			

Acquisition Parameter

Source Type	ESI	Ion Polarity	Positive	Set Nebulizer	3.0 Bar
Focus	Not active			Set Dry Heater	30 • ‹C
Scan Begin	50 m/z	Set Capillary	4500 V	Set Dry Gas	6.0 l/min
Scan End	3000 m/z	Set End Plate Offset	-500 V	Set Divert Valve	Waste

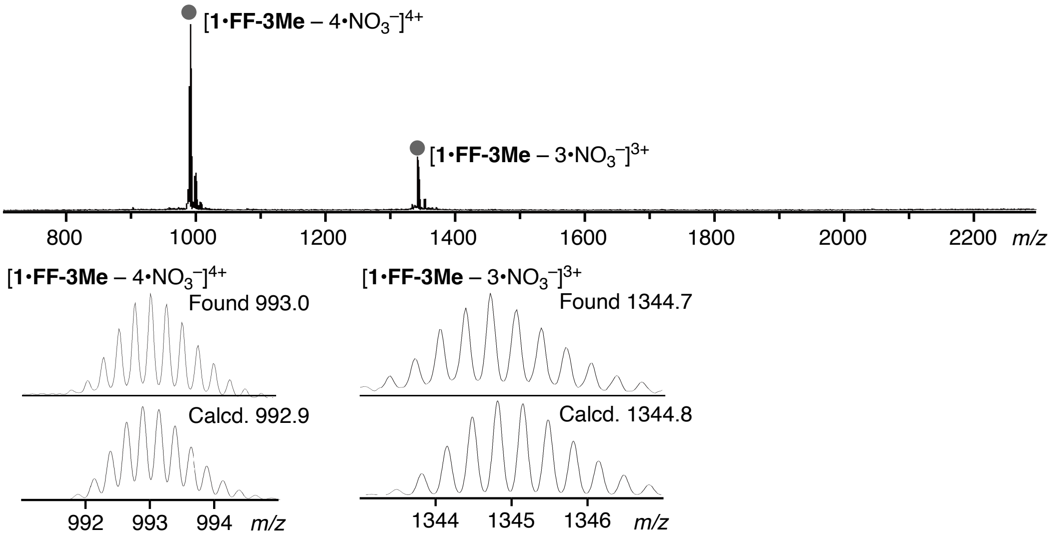

Figure S9b. ESI-TOF MS spectrum (H_2O) of **1•FF-3Me**.

Competitive binding of FF and CE or Asp by 1 MS072, 074, 286

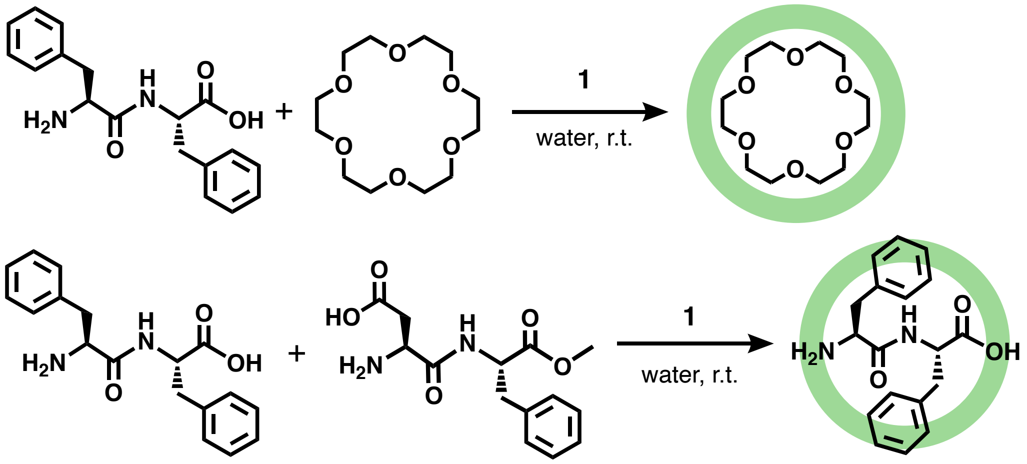

Receptor **1** (0.5 mg, 0.14 μmol), **FF** (0.04 mg, 0.14 μmol), and 18-crown-6 (**CE**: 0.04 mg, 0.14 μmol) were added to a 2 mL test tube containing D_2O (0.5 mL). The mixture was stirred at r.t. for 30 min. The selective formation of 1:1 host-guest complex **1•CE** was confirmed by ^{1}H NMR and ESI-TOF MS analyses. In the same way, receptor **1** (0.5 mg, 0.14 μmol), **FF** (0.04 mg, 0.14 μmol), and aspartame (**Asp**: 0.04 mg, 0.14 μmol) were added to a 2 mL test tube containing D_2O (0.5 mL). The mixture was stirred at r.t. for 30 min. The selective formation of 1:1 host-guest complex **1•FF** was confirmed by ^{1}H NMR and ESI-TOF MS analyses.

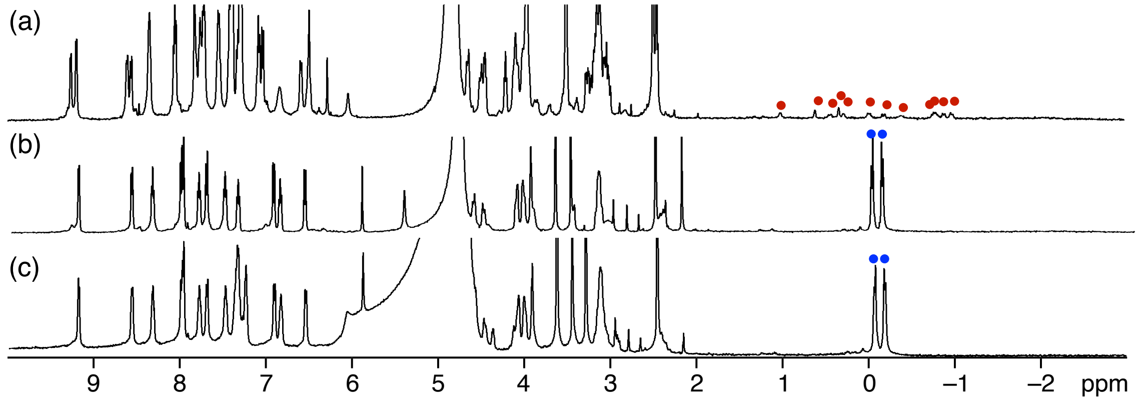

Figure S10a. ^{1}H NMR spectra (500 MHz, D_2O, r.t.) of (a) **1•FF**, (b) **1•CE**, and (c) products after mixing **FF** and **CE** with **1** at r.t. for 30 min.

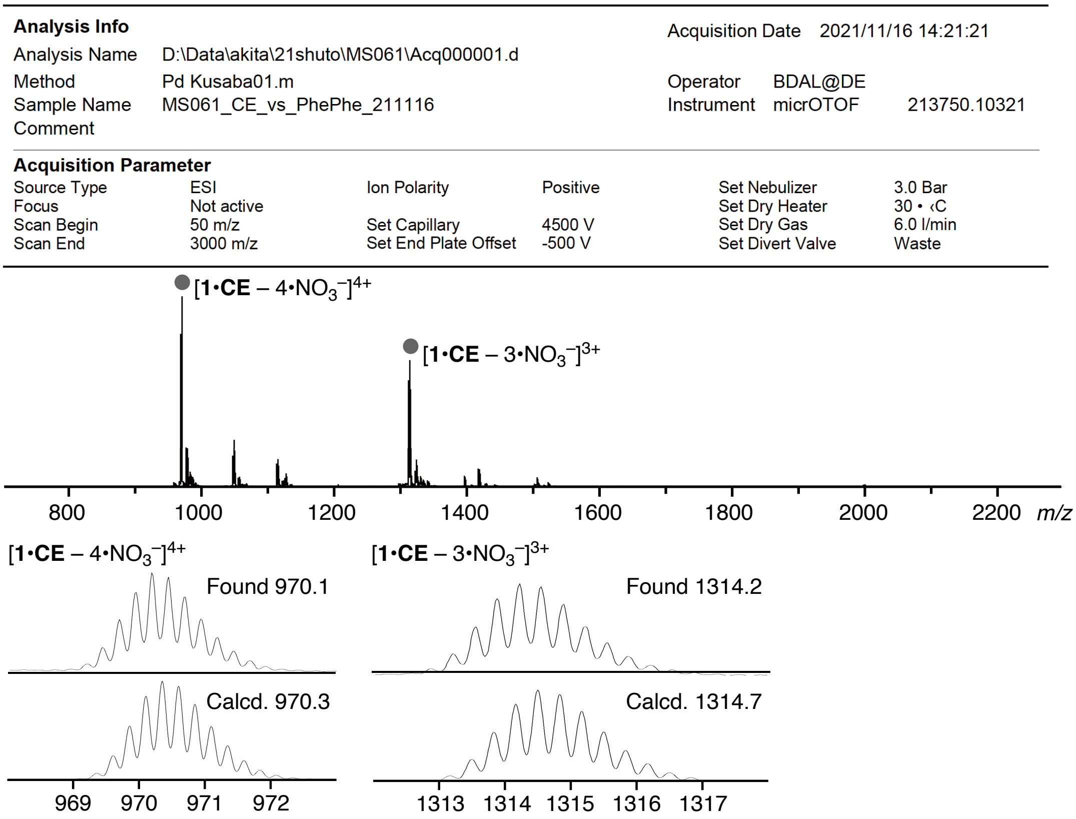

Figure S10b. ESI-TOF MS spectrum (H_2O) of products after mixing **FF** and **CE** with **1** at r.t. for 30 min.

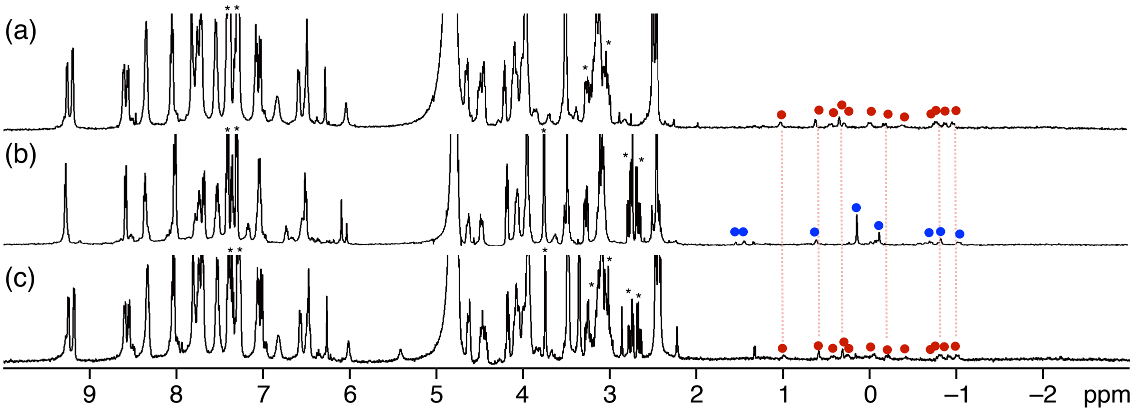

Figure S10c. ^{1}H NMR spectra (500 MHz, D_2O, r.t.) of (a) **1•FF**, (b) **1•Asp**, and (c) products after mixing **FF** and **Asp** with **1** at r.t. for 30 min (*: free **FF** and **Asp**).

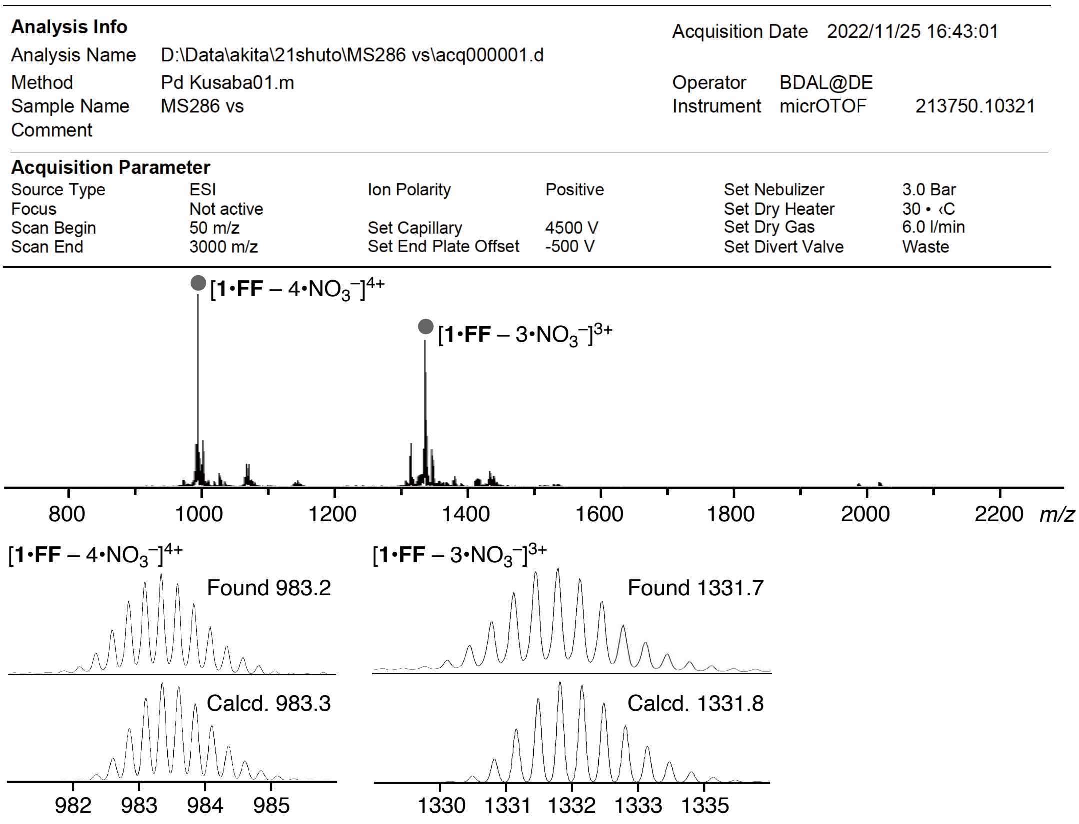

Figure S10d. ESI-TOF MS spectrum (H_2O) of products after mixing **FF** and **Asp** with **1** at r.t. for 30 min.

Thermodynamic studies of 1•FF, 1•FF-Me, and 1•FF-3Me MS146, 148, 161, 168, 230

Isothermal titration calorimetry (ITC) measurements were performed by dropping H_2O solutions (6.0 μL each) of **FF** (5.4 mM) to a H_2O solution of **1** (0.41 mM, 1.46 mL) at 25 ºC and H_2O solutions (9.0 μL each) of **FFF** (1.5 mM) to a H_2O solution of **1** (0.14 mM, 1.46 mL) at 25 ºC. In the same way, H_2O solutions of **F** (6.0 mM, 6.0 μL each), **FF-Me** (5.1 mM, 6.0 μL each), or **FF-3Me** (5.3 mM, 6.0 μL each) were dropped into a H_2O solution of **1** (0.12-0.18 mM, 1.46 mL) at 25 ºC.

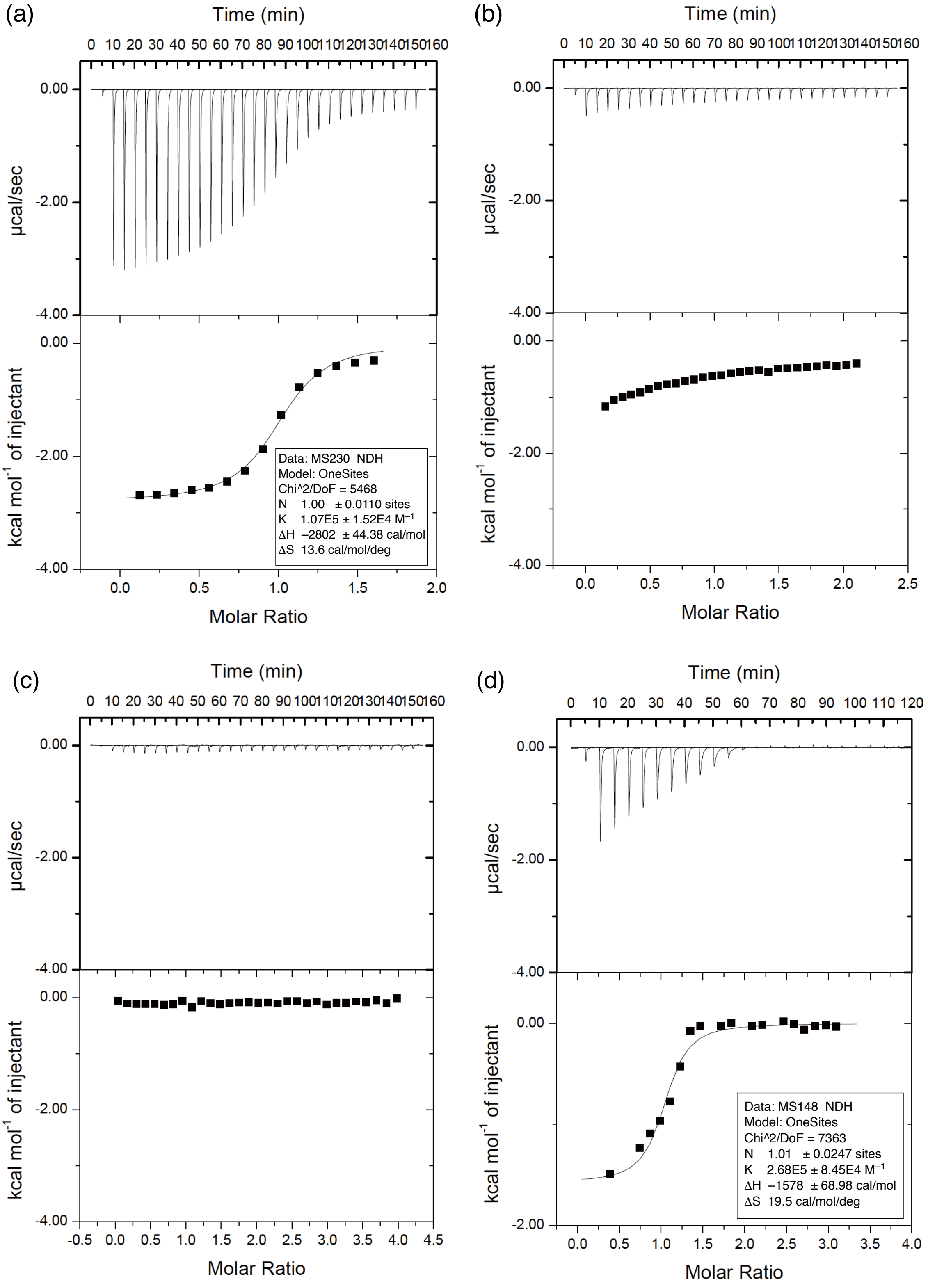
(a)
Time (min)
µcal/sec
kcal mol-1 of injectant
Molar Ratio
Data: MS230_NDH
Model: OneSites
Chi^2/DoF = 5468
N 1.00 ± 0.0110 sites
K 1.07E5 ± 1.52E4 M−1
ΔH −2802 ± 44.38 cal/mol
ΔS 13.6 cal/mol/deg
(b)
Time (min)
µcal/sec
kcal mol-1 of injectant
Molar Ratio
(c)
Time (min)
µcal/sec
kcal mol-1 of injectant
Molar Ratio
(d)
Time (min)
µcal/sec
kcal mol-1 of injectant
Molar Ratio
Data: MS148_NDH
Model: OneSites
Chi^2/DoF = 7363
N 1.01 ± 0.0247 sites
K 2.68E5 ± 8.45E4 M−1
ΔH −1578 ± 68.98 cal/mol
ΔS 19.5 cal/mol/deg

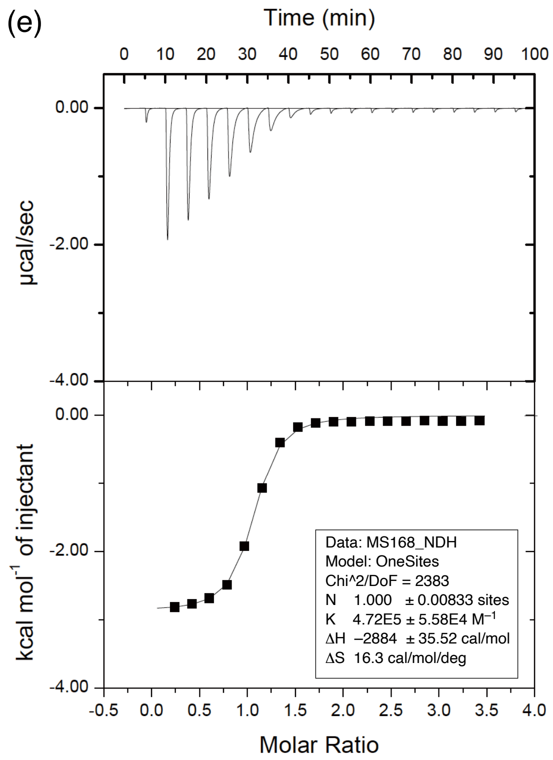

Figure S11. ITC thermographs (H_2O, 25 ºC) and their titration curves of a) **FF** to **1**, b) **FFF** to **1**, c) **F** to **1**, d) **FF-Me** to **1**, and e) **FF-3Me** to **1**. The solid line represents the best-fitting curve obtained from the "one sites" model.

Table S2. Thermodynamic parameters and binding constants (K_a) for the formation of complexes **1•FF**, **1•FF-Me** and **1•FF-3Me**, as obtained by ITC experiments (H_2O, 298 K).

complex	ΔH [kJ mol^{-1}]	$T\Delta S$ [kJ mol^{-1}]	ΔG [kJ mol^{-1}]	K_a / 10^5 [M^{-1}]
**1•FF**	–11.7	17.0	–28.7	1.07
**1•FF-Me**	–6.61	24.4	–31.0	2.68
**1•FF-3Me**	–12.1	20.2	–32.3	4.72

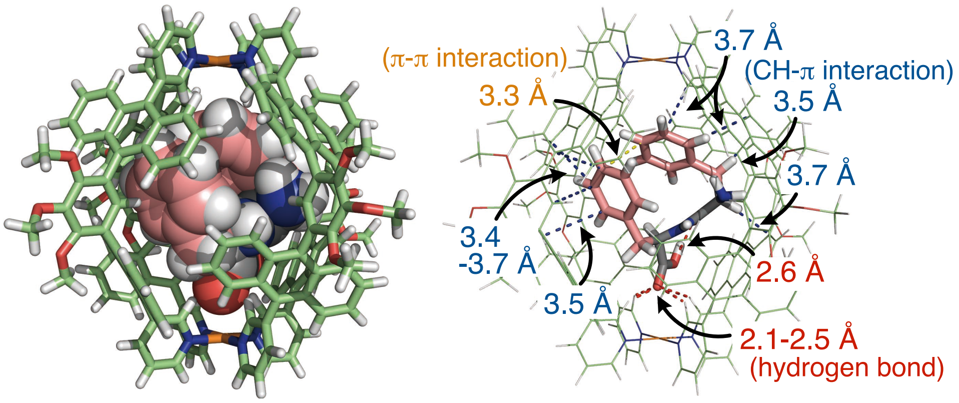

Figure S12. Host-guest interactions of **1•FF** (PM6 calculation).

Competitive binding of FF, LF, and LL by 1   MS059, 290

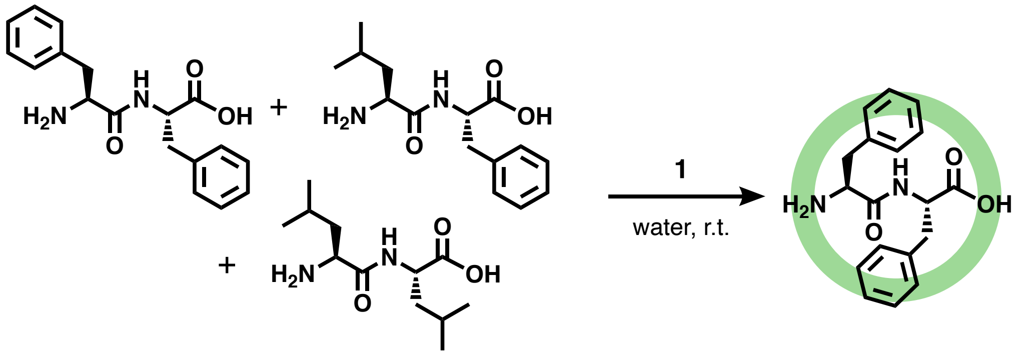

Receptor **1** (1.0 mg, 0.27 μmol), **FF** (0.08 mg, 0.27 μmol), L-leucine-L-phenylalanine (**LF**; 0.08 mg, 0.27 μmol), and L-leucine-L-leucine (**LL**; 0.07 mg, 0.27 μmol) were added to a 2 mL test tube containing D_2O (0.5 mL). The mixture was stirred at r.t. for 30 min. The selective formation of 1:1 host-guest complex **1•FF** was confirmed by ^{1}H NMR analyses. After the isolation of the products via salting out by addition of KNO_3 (1.5 mg), the selective formation of **1•FF** was further confirmed by ESI-TOF MS analysis. In the same way, receptor **1** (0.5 mg, 0.13 μmol), **FF** (0.04 mg, 0.13 μmol), **LF** (0.38 mg, 1.30 μmol), and **LL** (0.34 mg, 1.30 μmol) were added to a 2 mL test tube containing D_2O (0.5 mL). The mixture was stirred at r.t. for 30 min. The selective formation of **1•FF** was confirmed by ^{1}H NMR and ESI-TOF MS analyses.

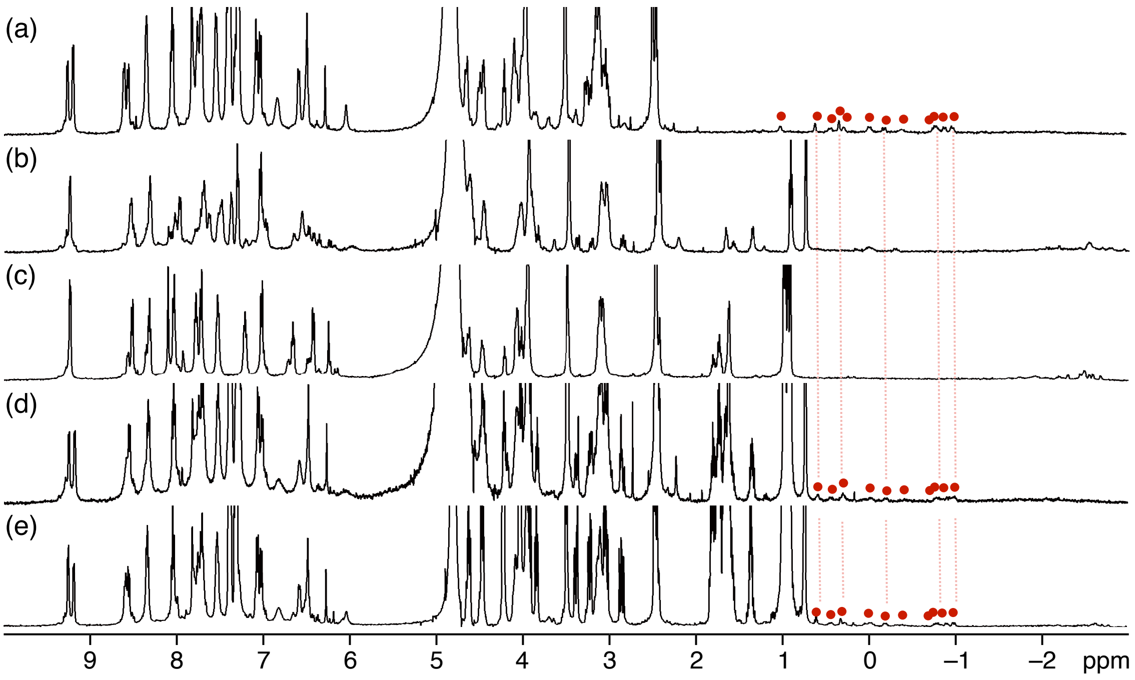

Figure S13a. ^{1}H NMR spectra (500 MHz, D_2O, r.t.) of (a) **1•FF**, (b) **1** + **LF**, (c) **1** + **LL**, (d) products after mixing **FF**, **LF**, and **LL**, and (e) products after mixing **FF**, **LF** (5 eq.), and **LL** (5 eq.) with **1** at r.t. for 30 min.

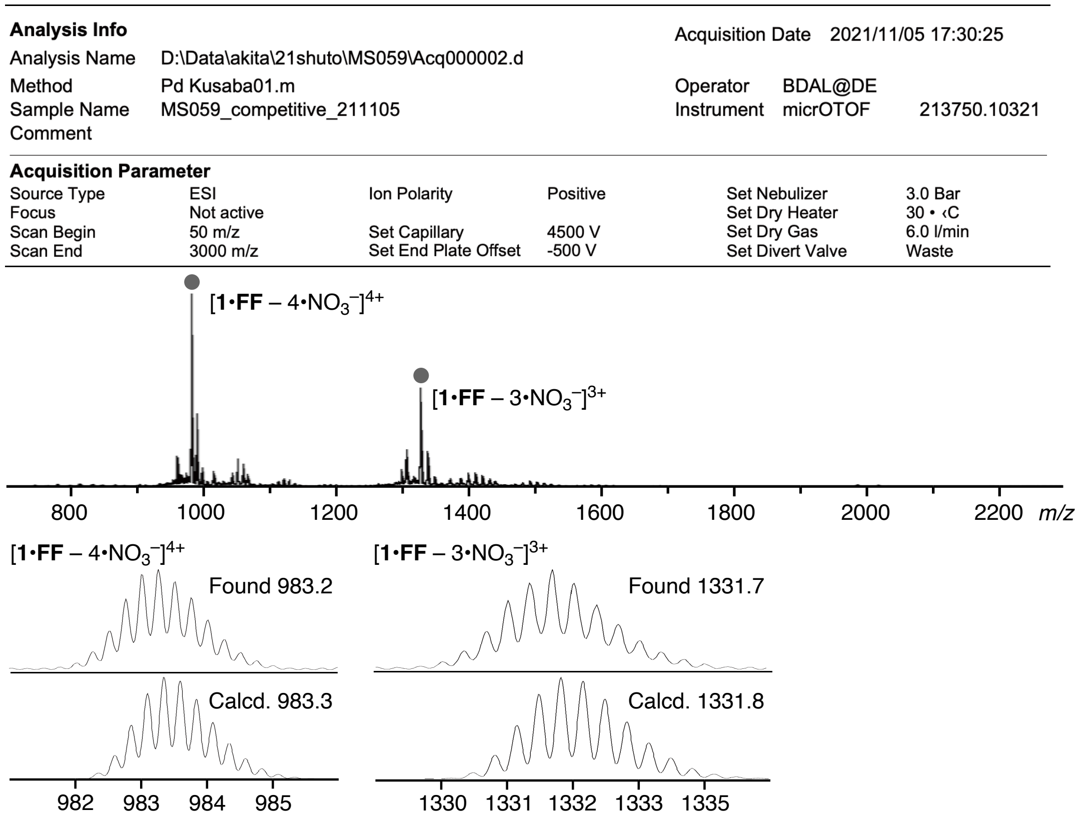

Figure S13b. ESI-TOF MS spectrum (H_2O) of products after mixing **FF**, **LF**, and **LL** with **1** at r.t. for 30 min.

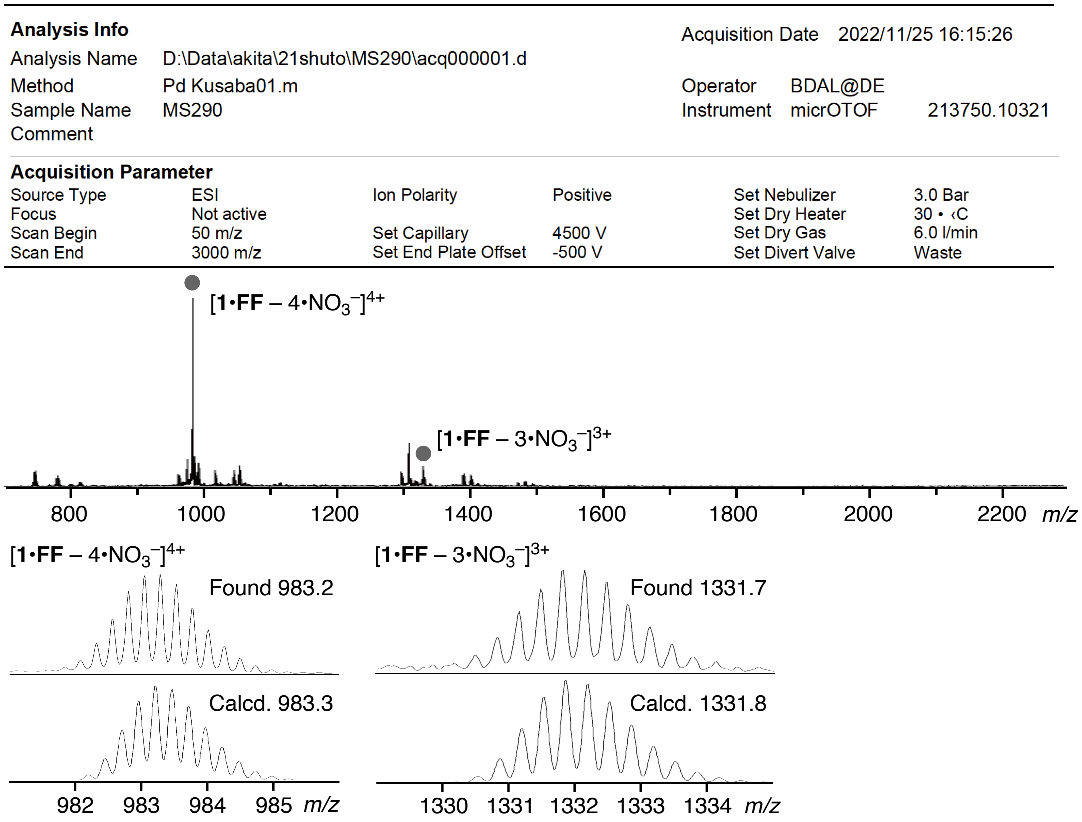

Figure S13c. ESI-TOF MS spectrum (H_2O) of products after mixing **FF**, **LF** (5 eq.), and **LL** (5

eq.) with **1** at r.t. for 30 min.

Competitive binding of FF and AAA or AAAA by 1   MS370, 371

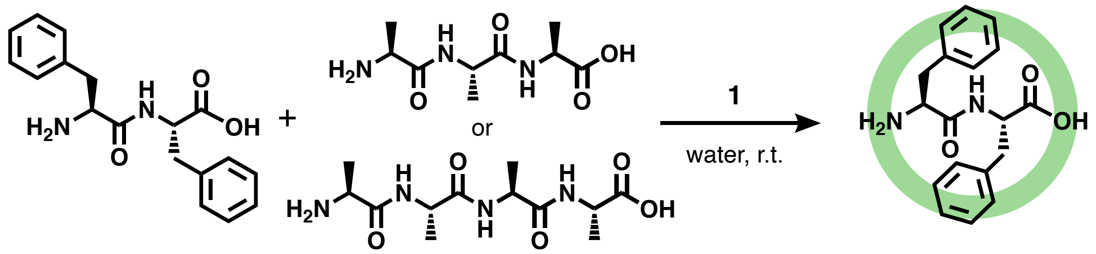

Receptor **1** (1.0 mg, 0.27 μmol), **FF** (0.08 mg, 0.27 μmol), and L-alanine-L-alanine-L-alanine (**AAA**: 0.06 mg, 0.27 μmol) were added to a 2 mL test tube containing D_2O (0.5 mL). The mixture was stirred at r.t. for 30 min. The selective formation of 1:1 host-guest complex **1•FF** was confirmed by ^{1}H NMR analysis. In the same way, receptor **1** (1.0 mg, 0.27 μmol), **FF** (0.08 mg, 0.27 μmol), and L-alanine-L-alanine-L-alanine-L-alanine (**AAAA**: 0.08 mg, 0.27 μmol) were added to a 2 mL test tube containing D_2O (0.5 mL). The mixture was stirred at r.t. for 30 min. The selective formation of **1•FF** was confirmed by ^{1}H NMR analysis.

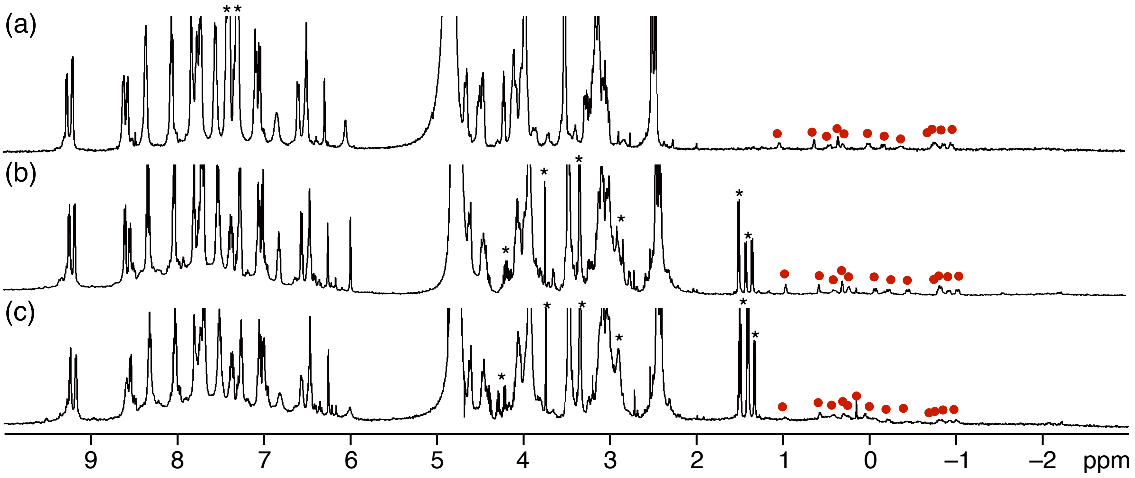

Figure S13d. ^{1}H NMR spectra (500 MHz, D_2O, r.t.) of (a) **1•FF**, products after mixing (b) **FF** and **AAA**, and (c) **FF** and **AAAA** with **1** at r.t. for 30 min. (*: free guest).

Synthesis of WW MS107-109

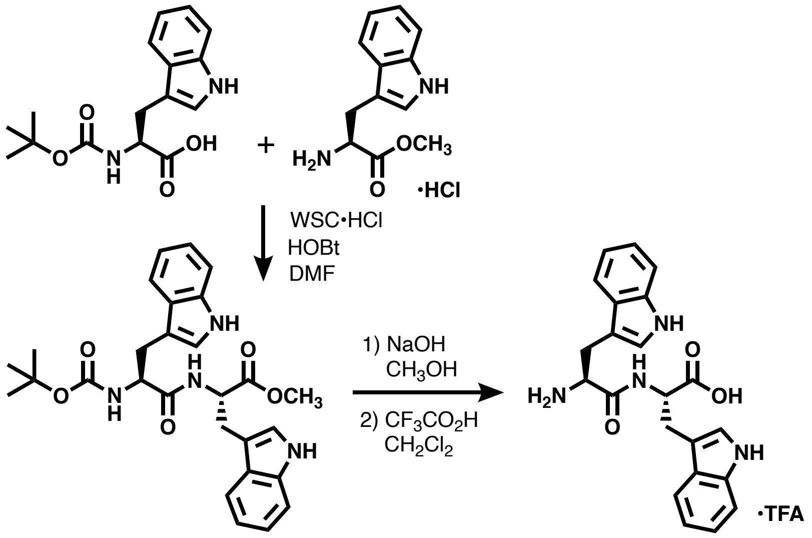

L-Tryptophan methyl ester hydrochloride (1.00 g, 3.93 mmol), *N*-(*tert*-butoxycarbonyl)-L-tryptophan (1.19 g, 4.12 mmol), HOBt (0.66 g, 4.32 mmol), WSC•HCl (0.90 g, 4.71 mmol), and DMF (40 mL) were added to a 100 mL glass flask. The reaction mixture was stirred at r.t. overnight. The resulting mixture was diluted with EtOAc (80 mL), washed with 1 M HCl aq. (20 mL), 10% $NaHCO_3$ aq. (20 mL), and brine (20 mL), and dried over $MgSO_4$. After the filteration and removal of the solvent, **BocWWCH$_3$** was obtained as a white solid (0.44 g, 0.87 mmol; 22%). **BocWWCH$_3$** (0.44 g, 0.89 mmol), methanol (5.0 mL), and NaOH (0.10 g, 2.17 mmol) was added to a 25 mL glass flask. The reaction mixture was stirred at r.t. overnight. After the evaporation of the solvent, 5% HCl was added to the solution (until pH 2) to yield a white solid. The solid was extracted with CH_2Cl_2. The resultant solution was dried over Na_2SO_4, filtrated, and concentrated under vacuum to give **BocWW** as a white solid (0.29 g, 0.59 mmol; 68%). **BocWW** (0.29 g, 0.59 mmol) and CH_2Cl_2 (3.0 mL) were added to a 25 mL glass flask. TFA (0.5 mL, 5.9 mmol) was added dropwise over a period of 10 min to the solution at 0 ℃. The mixture was stirred at r.t. for 30 min. After the removal of the solvent at 40 ℃ and washing with diethyl ether, **WW** was obtained as a white solid (0.19 g, 0.50 mmol; 84%).[S6]

WW: ^{1}H NMR (400 MHz, D_2O, r.t.): δ 3.24-3.35 (m, 3H), 3.63 (m, 1H), 4.26 (m, 1H), 4.68 (m, 1H), 7.17-7.32 (m, 6H), 7.55-7.69 (m, 4H). ESI-TOF MS (CH_3OH): *m*/*z* 392.1 $[M + H^+]^+$, 432.1 $[M + H^+ + CH_3CN]^+$.

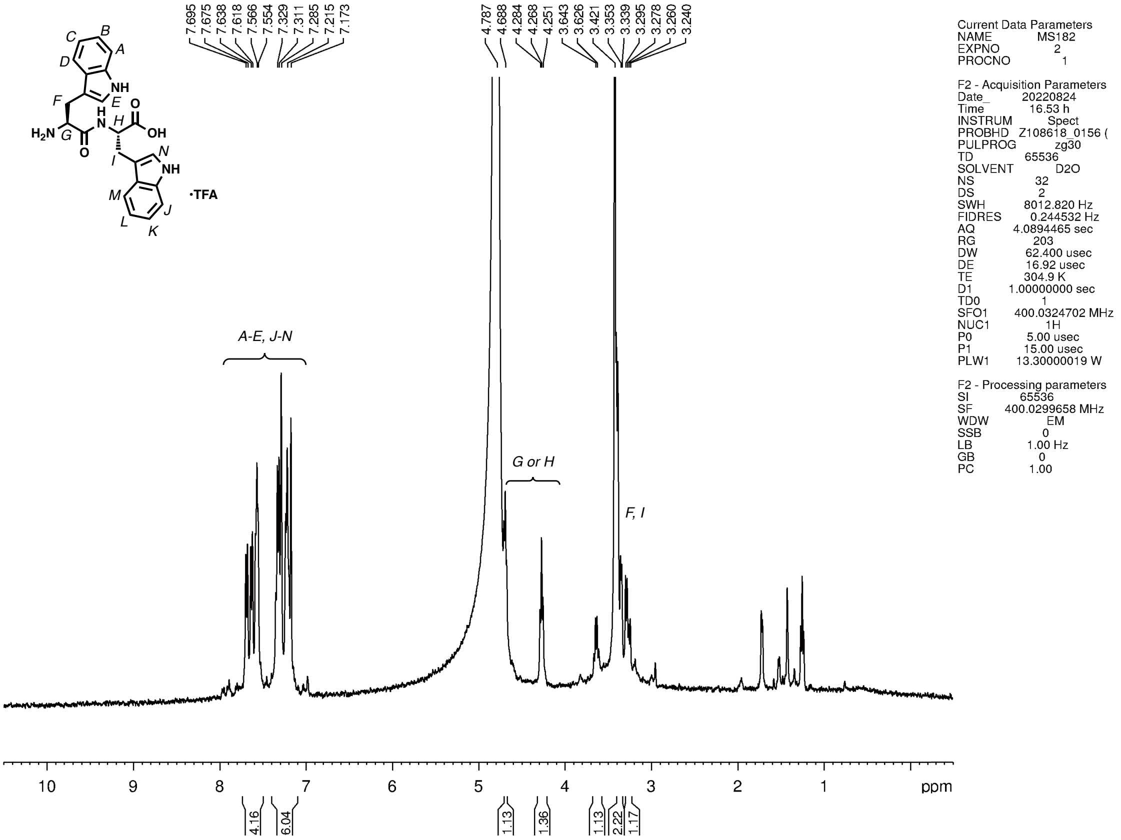

Figure S14a. ^{1}H NMR spectrum (400 MHz, D_2O, r.t.) of **WW**.

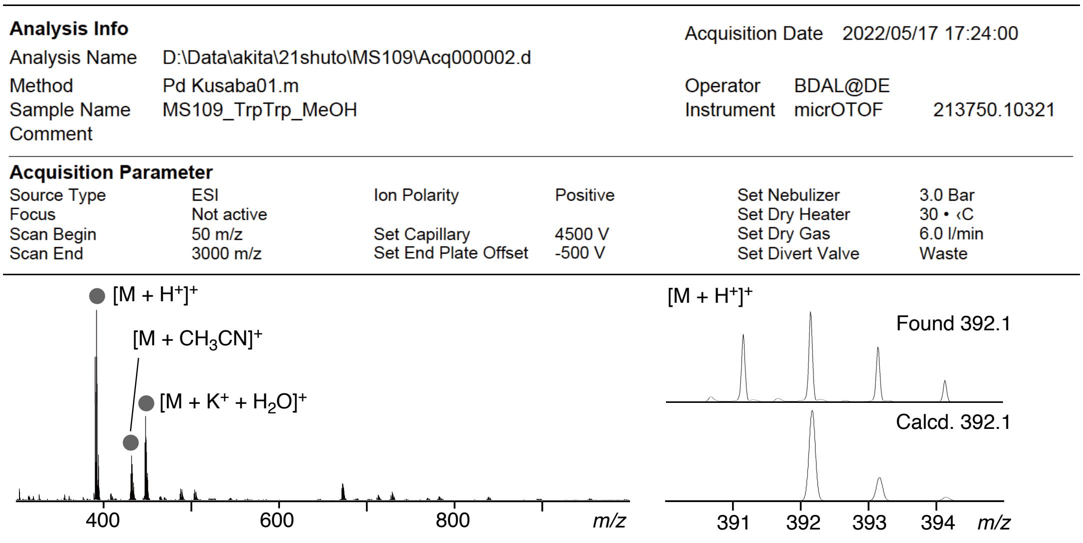

Figure S14b. ESI-TOF MS spectrum (CH_3OH) of **WW**.

Synthesis of WF MS159, 162, 171

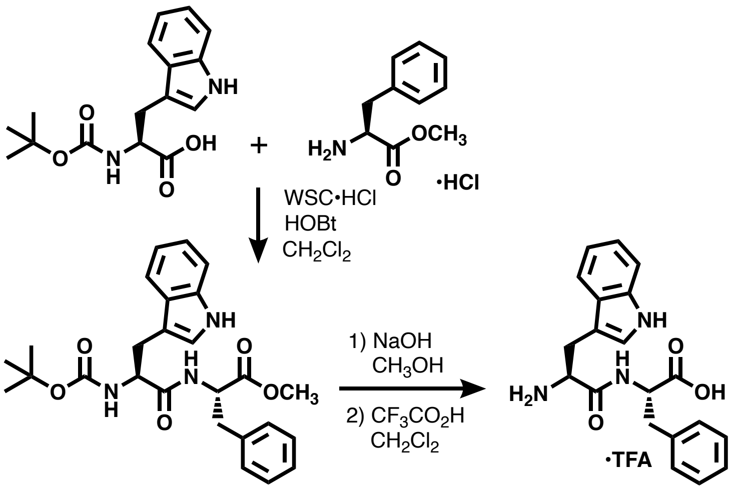

L-Phenylalanine methyl ester hydrochloride (0.38g, 1.77 mmol), *N*-(*tert*-butoxycarbonyl)-L-tryptophan (0.57 g, 1.87 mmol), HOBt (0.30 g, 1.95 mmol), WSC•HCl (0.41 g, 2.13 mmol), triethylamine (0.49 mL, 3.52 mmol), and CH_2Cl_2 (20 mL) were added to a 100 mL glass flask. The reaction mixture was stirred at r.t. overnight. The resulting mixture was diluted with CH_2Cl_2 (40 mL), washed with 1 M HCl aq. (15 mL) and dried over $MgSO_4$. After the filteration and removal of the solvent, **BocWFCH$_3$** was obtained as a white solid (0.91 g, 1.68 mmol; 89%). **BocWFCH$_3$** (0.91 g, 1.68 mmol), CH_3OH (7.0 mL), and NaOH (0.17 g, 4.20 mmol) was added to a 25 mL glass flask. The reaction mixture was stirred at r.t. overnight. After the evaporation of the solvent, 5% HCl was added to the solution (until pH 2) to yield a white solid. The solid was extracted with CH_2Cl_2. The resultant solution was dried over Na_2SO_4, filtrated, and concentrated under vacuum to give **BocWF** as a white solid (0.87 g, 1.93 mmol; 46%). **BocWF** (0.87 g, 1.93 mmol) and CH_2Cl_2 (10.0 mL) were added to a 25 mL glass flask. TFA (1.4 mL, 19.3 mmol) was added dropwise over a period of 10 min to the solution at 0 ºC. The mixture was stirred at r.t. for 30 min. After the removal of the solvent at 40 ºC and washing with diethyl ether, **WF** was obtained as a white solid (0.38 g, 1.08 mmol; 56%).[S7]

WF: ^{1}H NMR (400 MHz, D_2O, r.t.): δ 3.03 (m, 1H), 3.18 (m, 1H), 3.40 (m, 1H), 4.27 (m, 1H), 4.59 (m, 1H), 7.21-7.38 (m, 8H), 7.55-7.65 (m, 2H). ESI-TOF MS (CH_3OH): *m*/*z* 352.1 $[M + H^+]^+$.

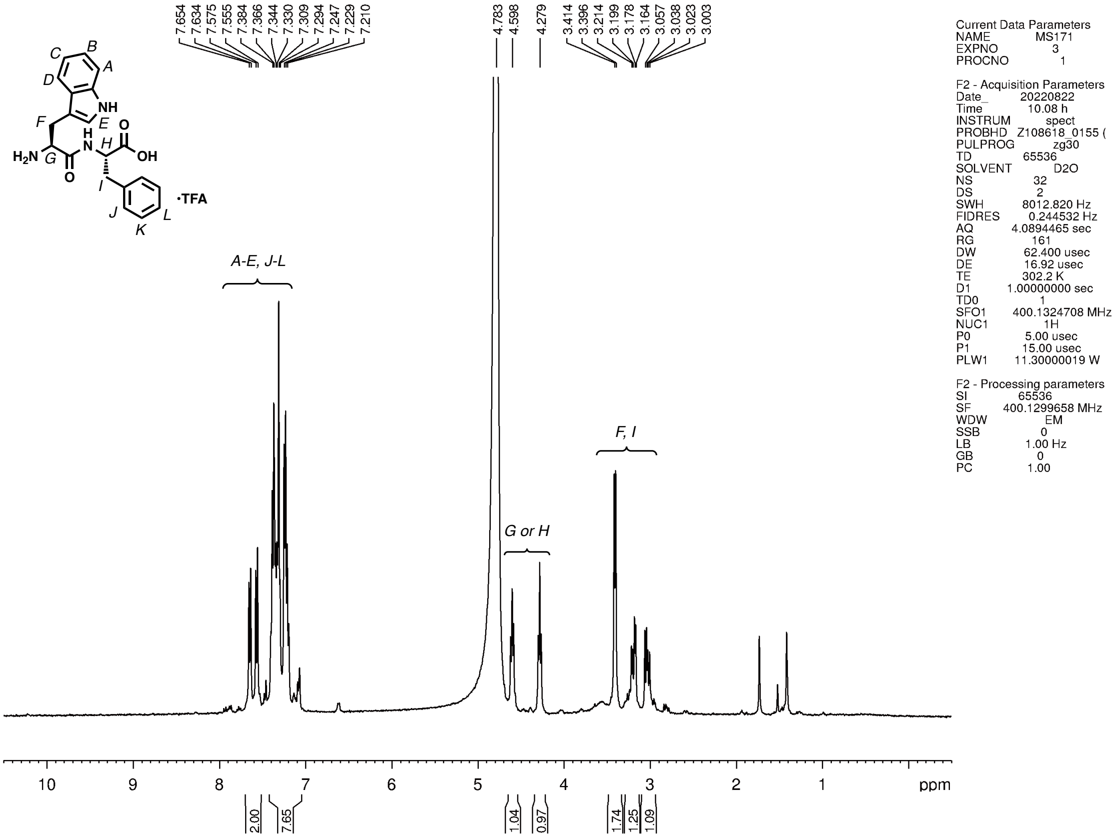

Figure S15a. ^{1}H NMR spectrum (400 MHz, D_2O, r.t.) of **WF**.

Analysis Info

Analysis Name	D:\Data\akita\21shuto\MS171\Acq000001.d	Acquisition Date	2022/08/02 15:36:21
Method	Pd Kusaba01.m	Operator	BDAL@DE
Sample Name	MS171_MeOH	Instrument	micrOTOF 213750.10321
Comment			

Acquisition Parameter

Source Type	ESI	Ion Polarity	Positive	Set Nebulizer	3.0 Bar
Focus	Not active			Set Dry Heater	30 • ‹C
Scan Begin	50 m/z	Set Capillary	4500 V	Set Dry Gas	6.0 l/min
Scan End	3000 m/z	Set End Plate Offset	-500 V	Set Divert Valve	Waste

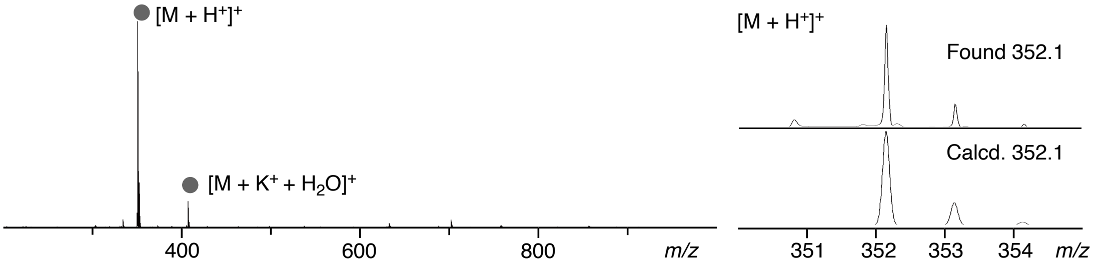

Figure S15b. ESI-TOF MS spectrum (CH_3OH) of **WF**.

Formation of 1•WF and 1•FW MS128, 263, RS1027

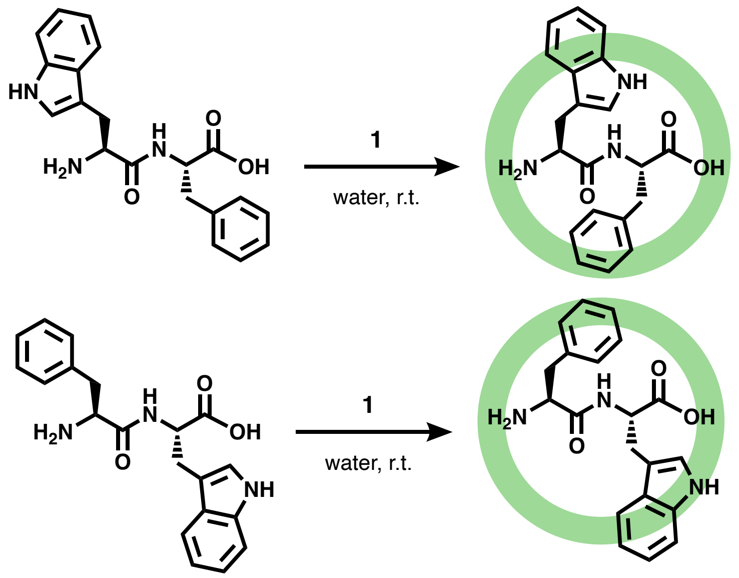

Receptor **1** (0.5 mg, 0.14 μmol) and **WF** (0.03 mg, 0.14 μmol) were added to a 2 mL test tube containing D_2O (0.5 mL). The mixture was stirred at r.t. for 30 min. The quantitative formation of 1:1 host-guest complex **1•WF** was confirmed by ^{1}H NMR, IR, ESI-TOF MS, and ITC analyses. In the same way, the selective formation (~95%) of 1:1 host-guest complex **1•FW** from **1** and **FW** in water was confirmed by NMR and ESI-TOF MS analyses.

^{1}H NMR (500 MHz, D_2O, r.t.): δ –0.34 (br, 1H, **WF**), –0.19 (br, 1H, **WF**), –0.06 (br, 1H, **WF**), 0.06 (br, 1H, **WF**), 0.16 (br, 1H, **WF**), 0.39 (br, 1H, **WF**), 0.64 (br, 1H, **WF**), 2.45-2.49 (br, 24H, $\mathbf{1}_{H_l}$), 3.13 (br, 16H, $\mathbf{1}_{H_k}$), 3.49 (s, 12H, $\mathbf{1}_{H_{l'}}$), 3.97-4.02 (br, 16H, $\mathbf{1}_{H_j}$), 4.12 (m, 8H, $\mathbf{1}_{H_{k'}}$), 4.50 (m, 4H, $\mathbf{1}_{H_{j'}}$), 4.65 (m, 4H, $\mathbf{1}_{H_{j''}}$), 5.98 (s, 0.5H, $\mathbf{1}_{H_a}$), 6.26 (s, 1.5H, $\mathbf{1}_{H_{a'}}$), 6.42-6.52 (d, J = 8.3 Hz, 1.6H, $\mathbf{1}_{H_e}$), 6.51 (d, J = 8.4 Hz, 3.6H, $\mathbf{1}_{H_{e'}}$), 6.62 (br, 4H, $\mathbf{1}_{H_d}$), 6.88 (br, 4H, $\mathbf{1}_{H_{d'}}$), 6.93-7.00 (br, 10.8H, $\mathbf{1}_{H_{e''}}$), 7.38-7.45 (br, 8H, $\mathbf{1}_{H_{d''}}$), 7.51 (m, 8H, $\mathbf{1}_{H_c}$), 7.72 (m, 8H, $\mathbf{1}_{H_b}$), 7.81 (m, 8H, $\mathbf{1}_{H_{c'}}$), 7.91 (s, 3H, $\mathbf{1}_{H_f}$), 8.05 (m, 8H, $\mathbf{1}_{H_{b'}}$), 8.12 (m, 5H, $\mathbf{1}_{H_{f'}}$), 8.32 (m, 8H, $\mathbf{1}_{H_f}$), 8.27-8.35 (br, 8H, $\mathbf{1}_{H_h}$), 8.50-8.62 (br, 8H, $\mathbf{1}_{H_i}$), 9.04-9.23 (br, 8H, $\mathbf{1}_{H_g}$). FT-IR (KBr, cm^{-1}): 1600 ($\nu_{C=O}$). ESI-TOF MS (H_2O): m/z 993.1 [**1•WF** – 4•NO_3^-]$^{4+}$, 1344.7 [**1•WF** – 3•NO_3^-]$^{3+}$, 2048.6 [**1•WF** – 2•NO_3^-]$^{2+}$.

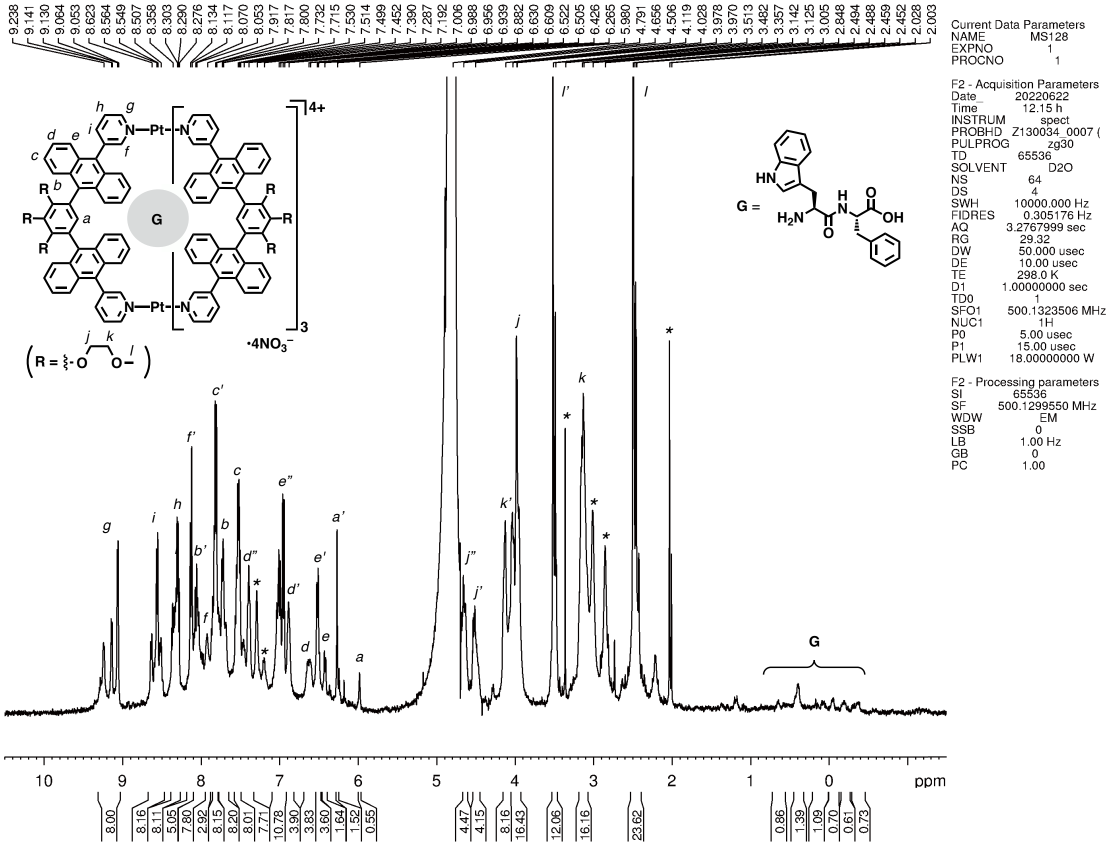

Figure S16a. ^{1}H NMR spectrum (500 MHz, D_2O, r.t.) of **1•WF** (*: free **WF**).

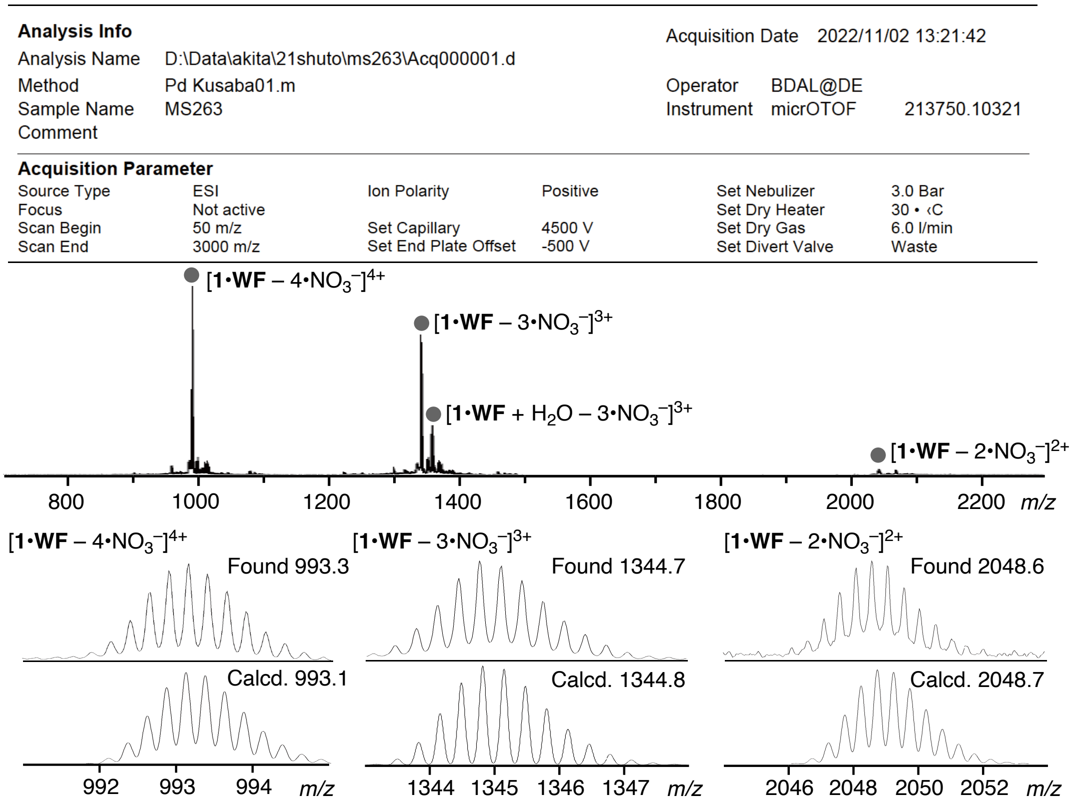

Figure S16b. ESI-TOF MS spectrum (H_2O) of **1•WF**.

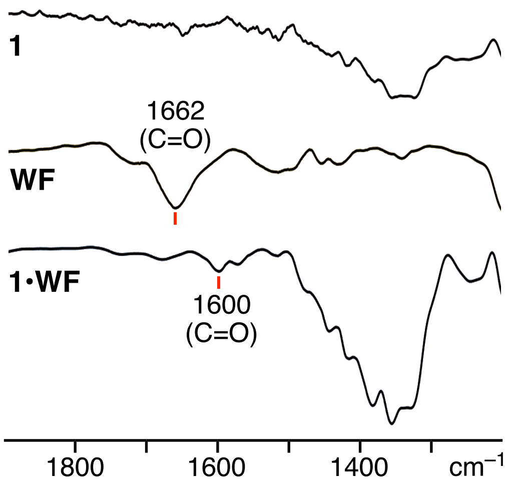

Figure S16c. FT-IR spectra (ATR, r.t.) of **1**, **WF**, and **1•WF**.

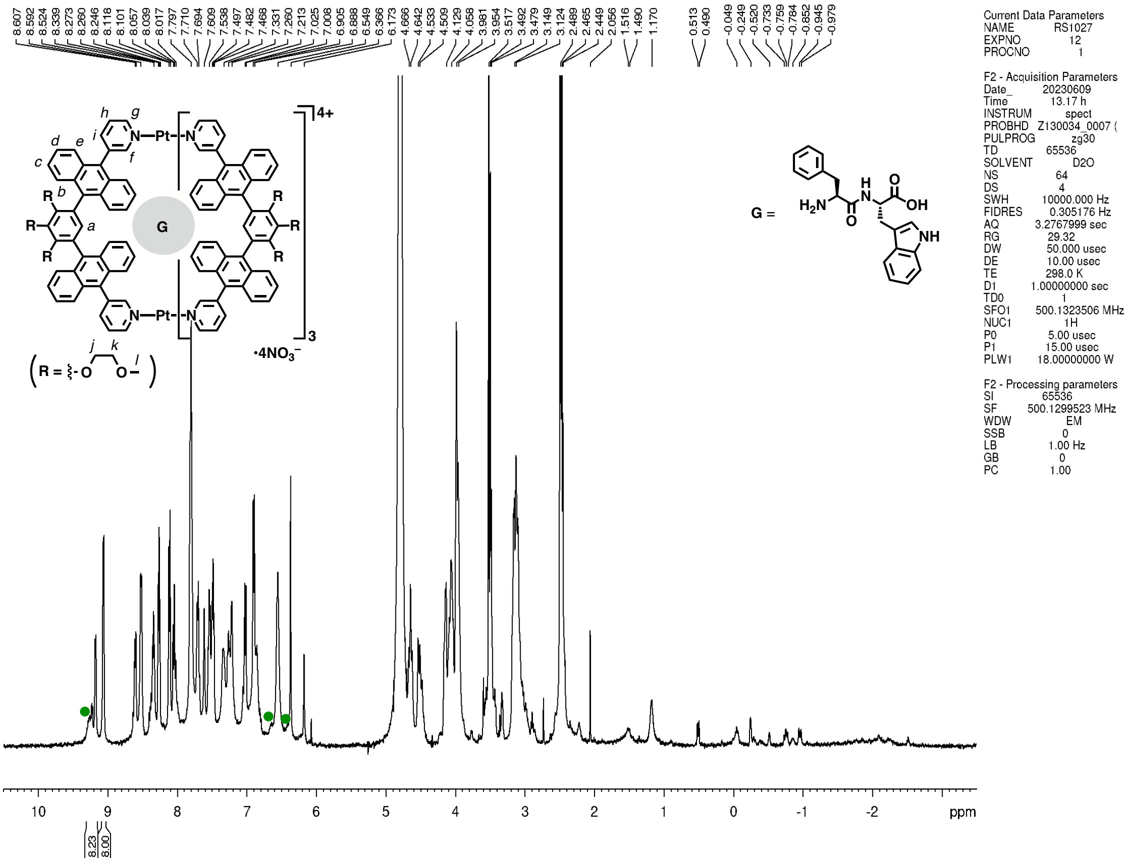

Figure S16d. ^{1}H NMR spectrum (500 MHz, D_2O, r.t.) of **1•FW** (green circle : free **1**).

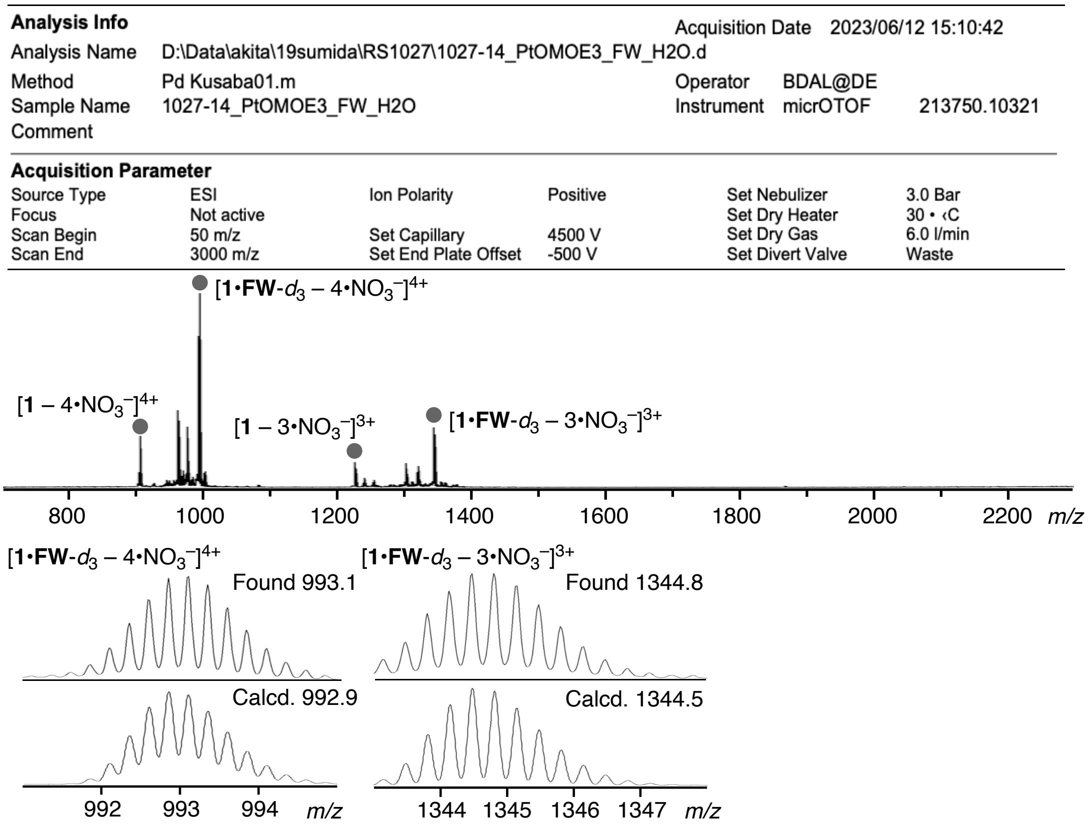

Figure S16e. ESI-TOF MS spectrum (H_2O) of **1•FW**.

Competitive binding of FF and WW by 1 MS113

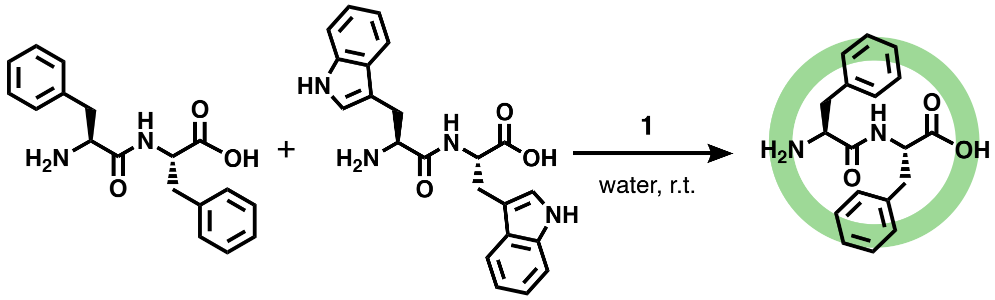

Receptor **1** (0.5 mg, 0.14 μmol), **FF** (0.03 mg, 0.14 μmol), and **WW** (0.05 mg, 0.14 μmol) were added to a 2 mL test tube containing D_2O (0.5 mL). The mixture was stirred at r.t. for 30 min. The selective formation of 1:1 host-guest complex **1•FF** was confirmed by ^{1}H NMR analysis. After the isolation of the products via salting out by addition of KNO_3 (1.5 mg), the selective formation of **1•FF** was further confirmed by ESI-TOF MS analysis.

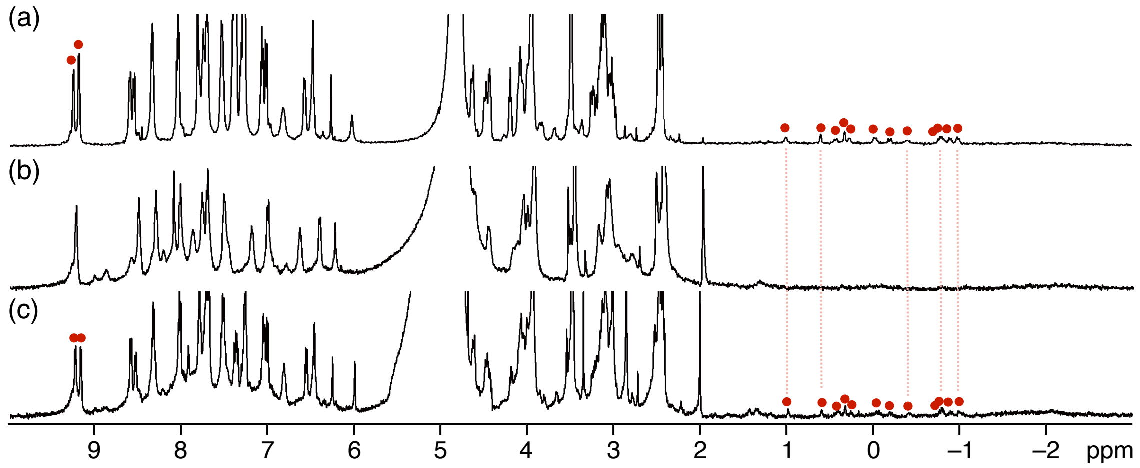

Figure S17a. ^{1}H NMR spectra (500 MHz, D_2O, r.t.) of (a) **1•FF**, (b) **1** + **WW**, and (c) products after mixing **FF** and **WW** with **1** at r.t. for 30 min.

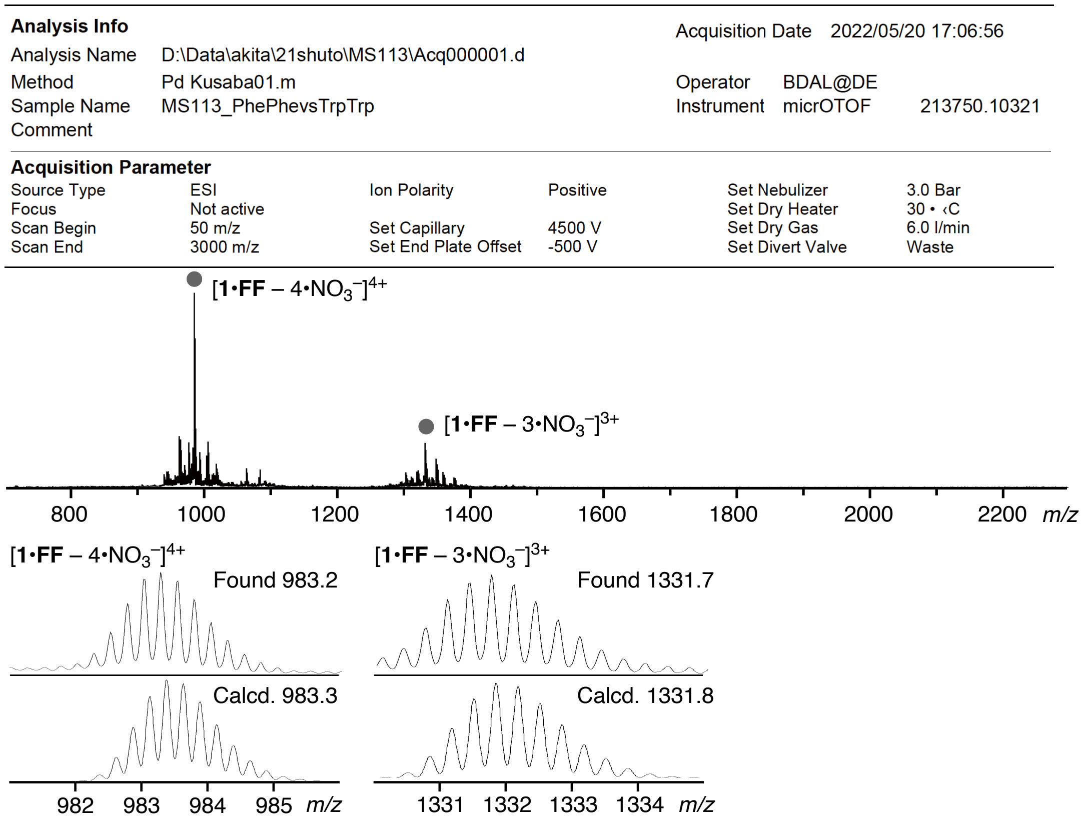

Figure S17b. ESI-TOF MS spectrum (H_2O) of products after mixing **FF** and **WW** with **1** at r.t. for 30 min

Competitive binding of FF, WF, and WW by 1 MS208, 261

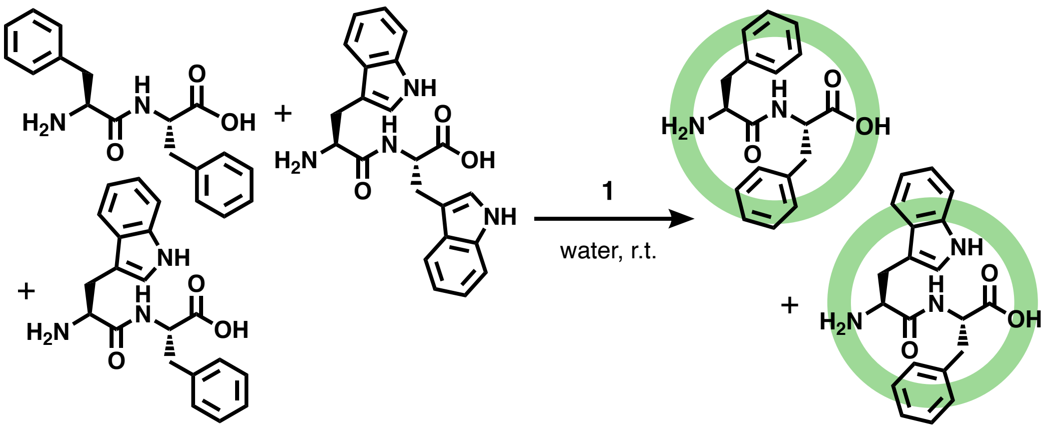

Receptor **1** (1.0 mg, 0.27 µmol), **FF** (0.08 mg, 0.27 µmol), **WF** (0.09 mg, 0.27 µmol), and **WW** (0.11 mg, 0.27 µmol) were added to a 2 mL test tube containing D_2O (0.5 mL). The mixture was stirred at r.t. for 30 min. The formation of 1:1 host-guest complex **1•FF** (55%) and **1•WF** (45%) were confirmed by ^{1}H NMR analysis. After the isolation of the products via salting out by addition of KNO_3 (1.5 mg), the formation of 1:1 host-guest complexes **1•FF** and **1•WF** were further confirmed by ESI-TOF MS analysis. On the other hand, when a mixture of receptor **1** (1.0 mg, 0.27 µmol), **FF** (0.08 mg, 0.27 µmol), **WF** (0.09 mg, 0.27 µmol), **WW** (0.11 mg, 0.27 µmol), and $NaNO_3$ (4.59 µg, 0.05 µmol) in D_2O (0.5 mL) was stirred at r.t. for 30 min, the formation of 1:1 host-guest complexes **1•FF** (75%) and **1•WF** (25%) were confirmed by ^{1}H NMR analysis.

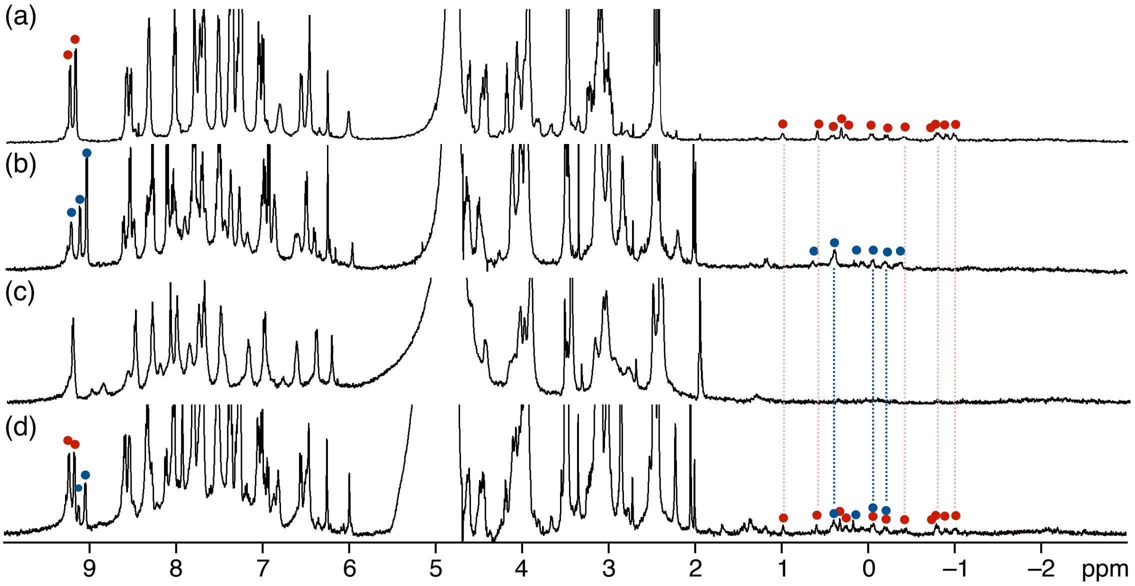

Figure S18a. ^{1}H NMR spectra (500 MHz, D_2O, r.t.) of (a) **1•FF**, (b) **1•WF**, (c) **1** + **WW**, and (d) products after mixing **FF**, **WF**, and **WW** with **1** at r.t. for 30 min.

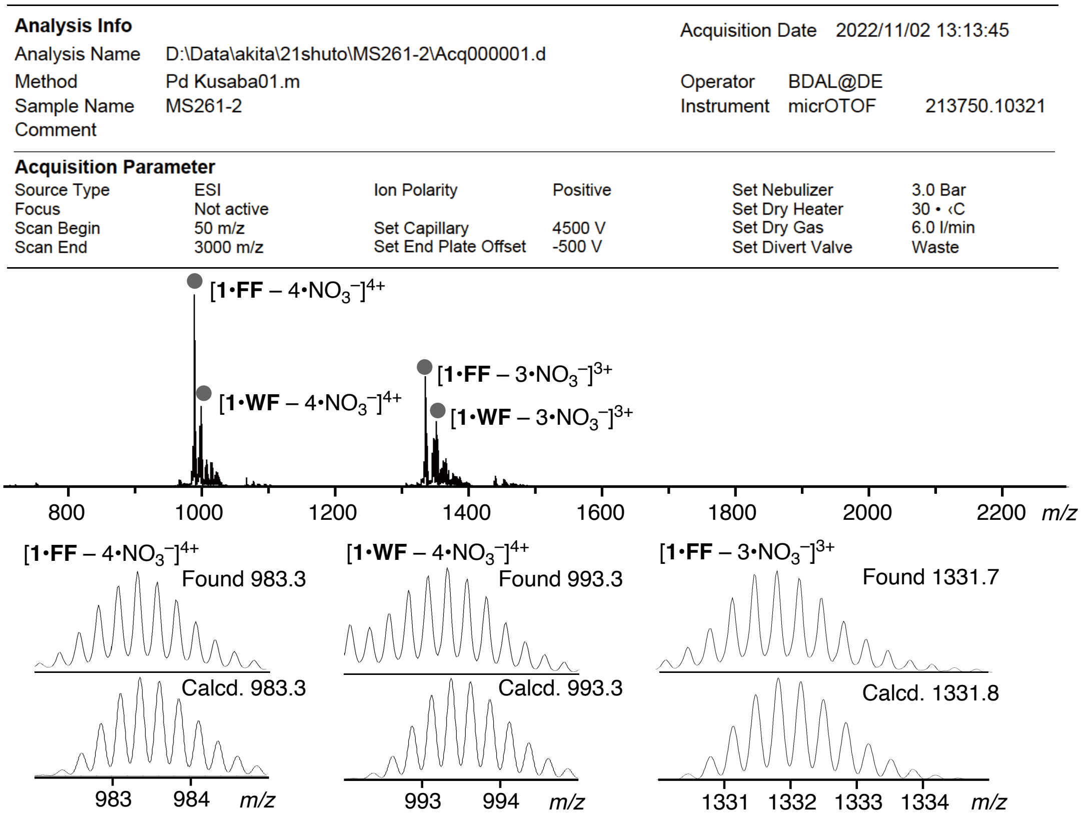

Figure S18b. ESI-TOF MS spectrum (H_2O) of products after mixing **FF**, **WF**, and **WW** with **1** at r.t. for 30 min.

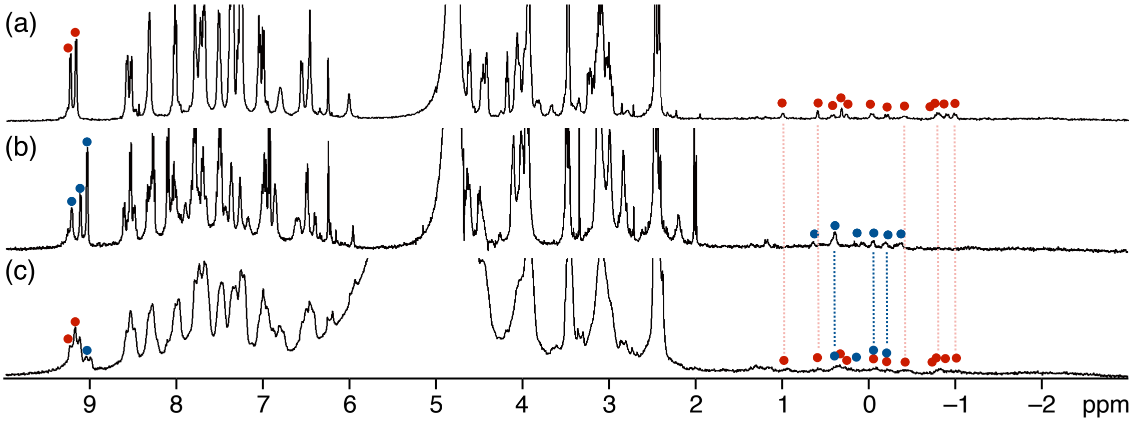

Figure S18c. ^{1}H NMR spectra (500 MHz, D_2O, r.t.) of (a) **1•FF**, (b) **1•WF**, and (c) products after mixing **FF** and **WF** with **1** in the presence of $NaNO_3$ (0.2 eq. based on **1**) at r.t. for 30 min.

Competitive binding of FF and FW by 1 RS1043

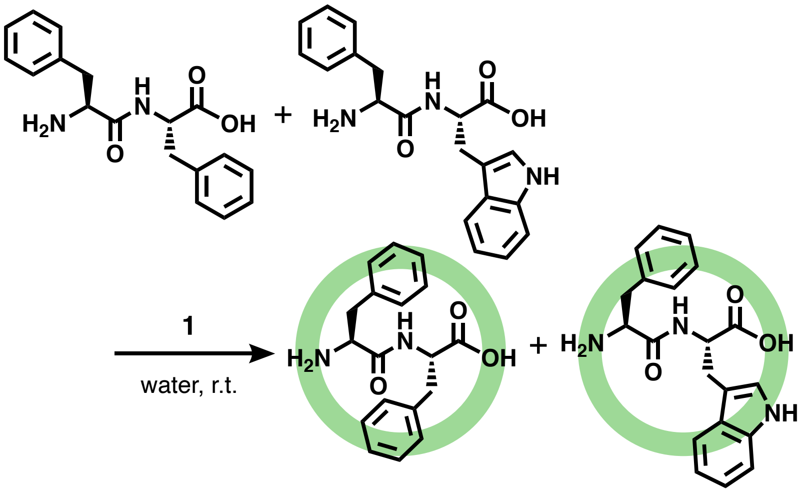

Receptor **1** (1.0 mg, 0.27 μmol), **FF** (0.08 mg, 0.27 μmol), and **FW** (0.09 mg, 0.27 μmol) were added to a 2 mL test tube containing D_2O (0.5 mL). The mixture was stirred at r.t. for 30 min. The formation of 1:1 host-guest complex **1•FF** (79%) and **1•FW** (21%) were confirmed by ^{1}H NMR analysis.

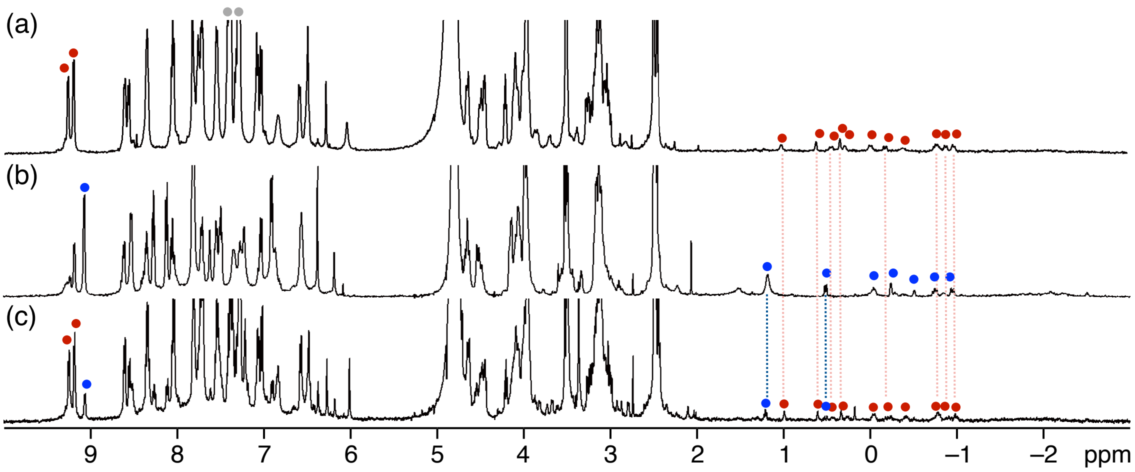

Figure S18d. ^{1}H NMR spectra (500 MHz, D_2O, r.t.) of (a) **1•FF**, (b) **1•FW**, and (c) products after mixing **FF** and **FW** with **1** at r.t. for 30 min.

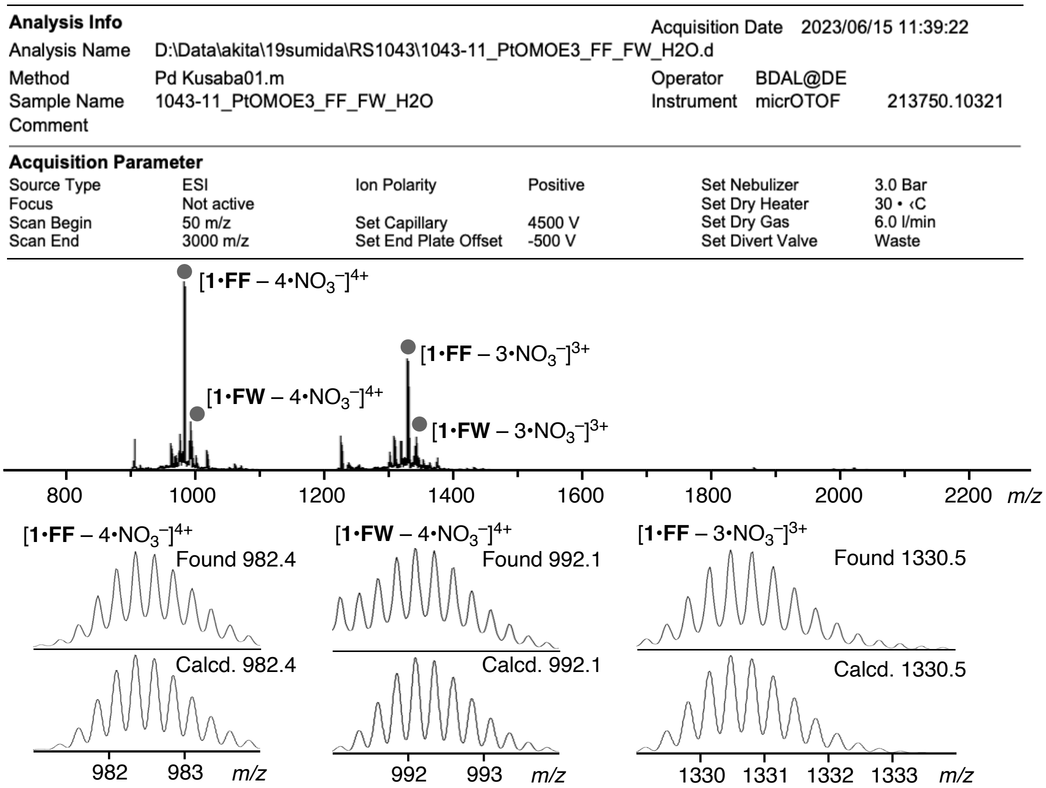

Figure S18e. ESI-TOF MS spectrum (H_2O) of products after mixing **FF** and **FW** with **1** at r.t. for 30 min.

Optimized structures and energies of 1•WF and 1•WW MS350, 351, 367

The geometry optimizations were performed with PM6 calculation. On the basis of the crystal structure of receptor **1**,[S1] randomly oriented guest **WF** or **WW** within receptor **1** (R = OCH_3) in several initial structures converged to a single host-guest structure. Energy changes ($\Delta E_{H\bullet G} = E_{H\bullet G} - (E_H + E_G)$ kJ/mol) of receptor **1** before and after the binding of **G** (= **WF** and **WW**) were estimated by DFT calculation (CAM-B3LYP-GD3BJ /LanL2DZ (Pt), 6-31G(d,p) (others), PCM (H_2O) level of theory). The geometry optimizations of **WF** and **WW** were performed in the same way.

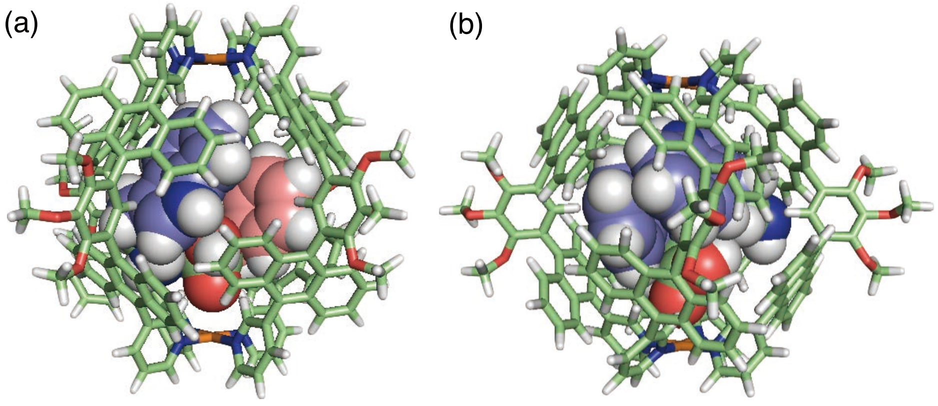

Figure S19a. Optimized structures (PM6 calculations) of (a) **1•WF** and (b) **1•WW**.

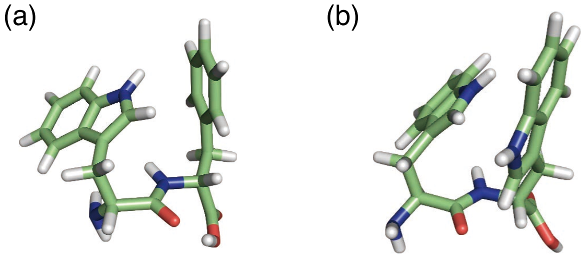

Figure S19b. Optimized structures (PM6 calculations) of (a) **WF** and (b) **WW**.

Table S3. Energy changes (kJ/mol) of receptor **1** before/after the binding of **FF**, **WF**, or **WW** (DFT calculations).

G	$E_{\mathbf{H}}$	$E_{\mathbf{G}}$	$E_{\mathbf{H\cdot G}}$	$\Delta E_{\mathbf{H\cdot G}}$
**FF**	–23157673.7	–2711447.9	–25869310.1	–188.5
**WF**	–23157673.7	–3056736.9	–26214541.9	–131.3
**WW**	–23157673.7	–3402039.4	–26559839.0	–126.0

Formation of 1•YF and 1•FY MS355, RS1026

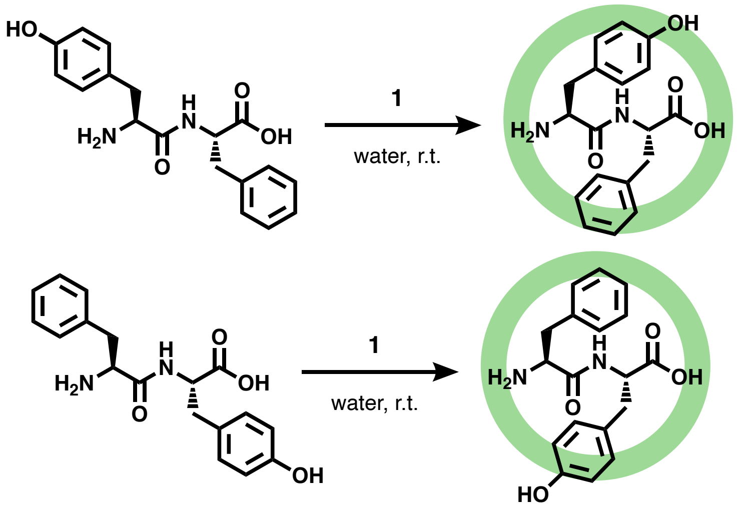

Receptor **1** (0.5 mg, 0.14 μmol) and L-tyrosine-L-phenylalanine (**YF**; 0.2 mg, 0.70 μmol) were added to a 2 mL test tube containing D_2O (0.5 mL). When the mixture was stirred at r.t. for 30 min, the formation of 1:1 host-guest complex **1•YF** (77%) was confirmed by ^{1}H NMR analysis. Host-guest complex **1•YF** was formed in >98% yield at 80 ºC for 1 h, which was confirmed by ^{1}H NMR and ESI-TOF MS analyses. In the same way, the formation of 1:1 host-guest complex **1•FY** (>95%) was confirmed at 80 ºC for 1 h by ^{1}H NMR analysis.

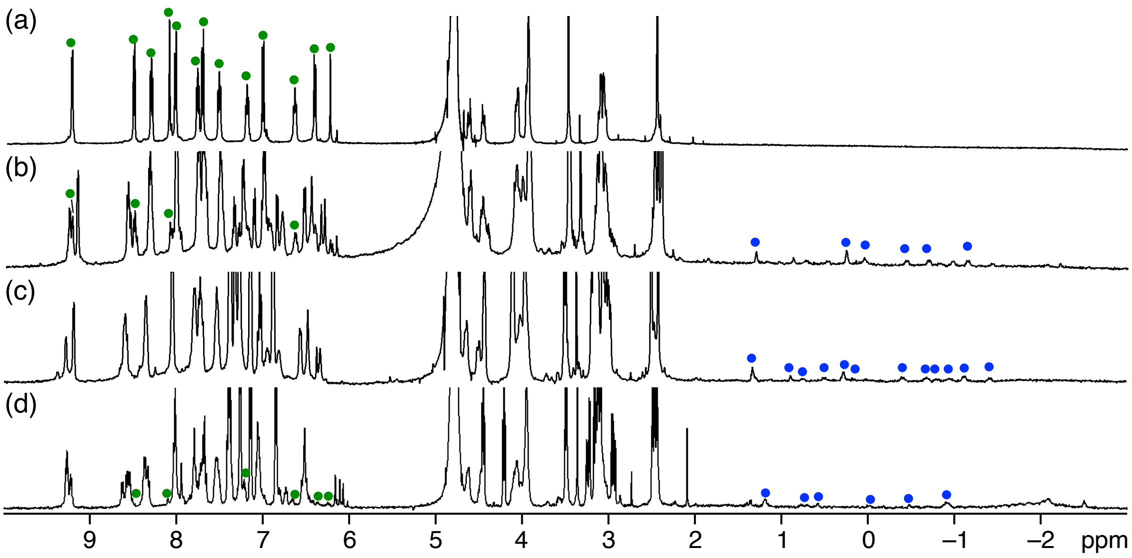

Figure S20a. ^{1}H NMR spectrum (500 MHz, D_2O, r.t.) of (a) **1**, (b) **1•YF** (reaction temp.: r.t.), (c) **1•YF** (80 ºC), and (c) **1•FY** (80 ºC).

**Analysis Info**		Acquisition Date	2023/02/28 14:01:48	
Analysis Name	D:\Data\akita\21shuto\MS355\Acq000001.d			
Method	Pd Kusaba01.m	Operator	BDAL@DE	
Sample Name	MS355	Instrument	micrOTOF	213750.10321
Comment				

**Acquisition Parameter**					
Source Type	ESI	Ion Polarity	Positive	Set Nebulizer	3.0 Bar
Focus	Not active			Set Dry Heater	30 • ‹C
Scan Begin	50 m/z	Set Capillary	4500 V	Set Dry Gas	6.0 l/min
Scan End	3000 m/z	Set End Plate Offset	-500 V	Set Divert Valve	Waste

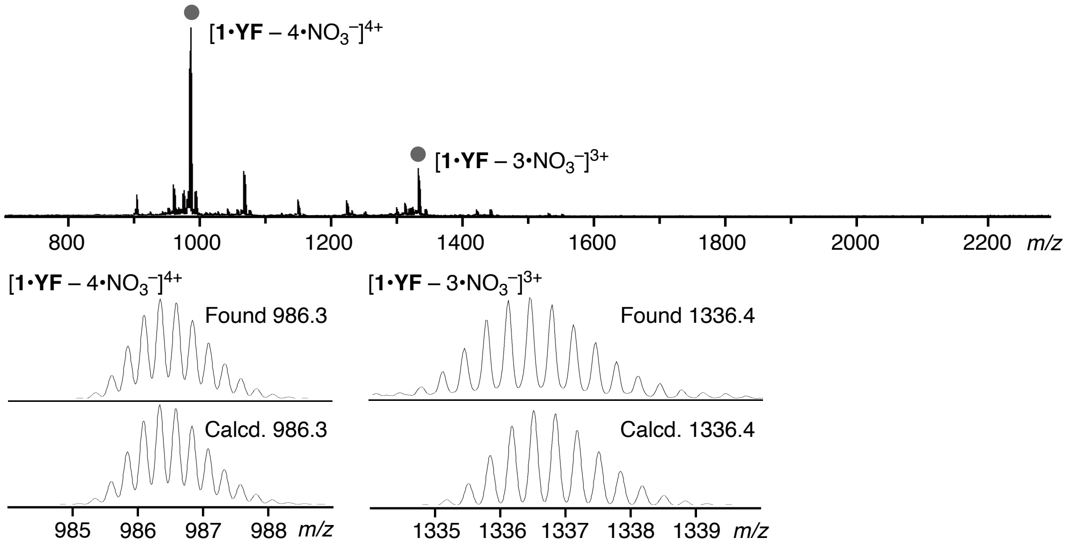

Figure S20b. ESI-TOF MS spectrum (H_2O) of **1•YF** (reaction temp.: 80 °C),

Competitive binding of FF and YF or FY by 1 MS341, RS1042

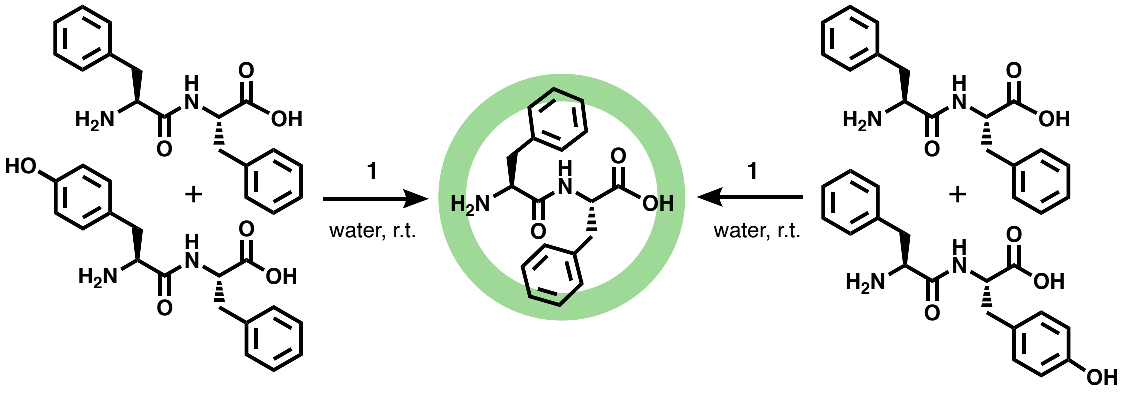

Receptor **1** (1.0 mg, 0.27 μmol), **FF** (0.08 mg, 0.27 μmol), and **YF** (0.09 mg, 0.27 μmol) were added to a 2 mL test tube containing D_2O (0.5 mL). The mixture was stirred at r.t. for 30 min. The selective formation of 1:1 host-guest complex **1•FF** was confirmed by ^{1}H NMR and ESI-TOF MS analyses. In the same way, the selective formation of 1:1 host-guest complex **1•FF** from a mixture of **1**, **FF**, and **FY** was confirmed by ^{1}H NMR and ESI-TOF MS analyses.

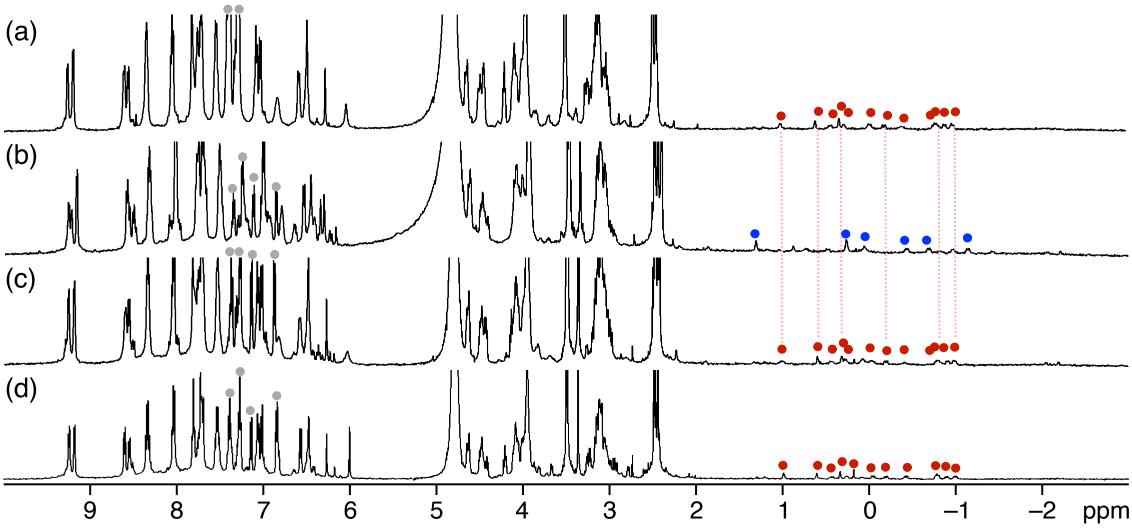

Figure S21a. ^{1}H NMR spectra (500 MHz, D_2O, r.t.) of (a) **1•FF**, (b) **1•YF**, (c) products after mixing **FF** and **YF** with **1** at r.t. for 30 min, and (d) products after mixing **FF** and **FY** with **1** at r.t. for 30 min.

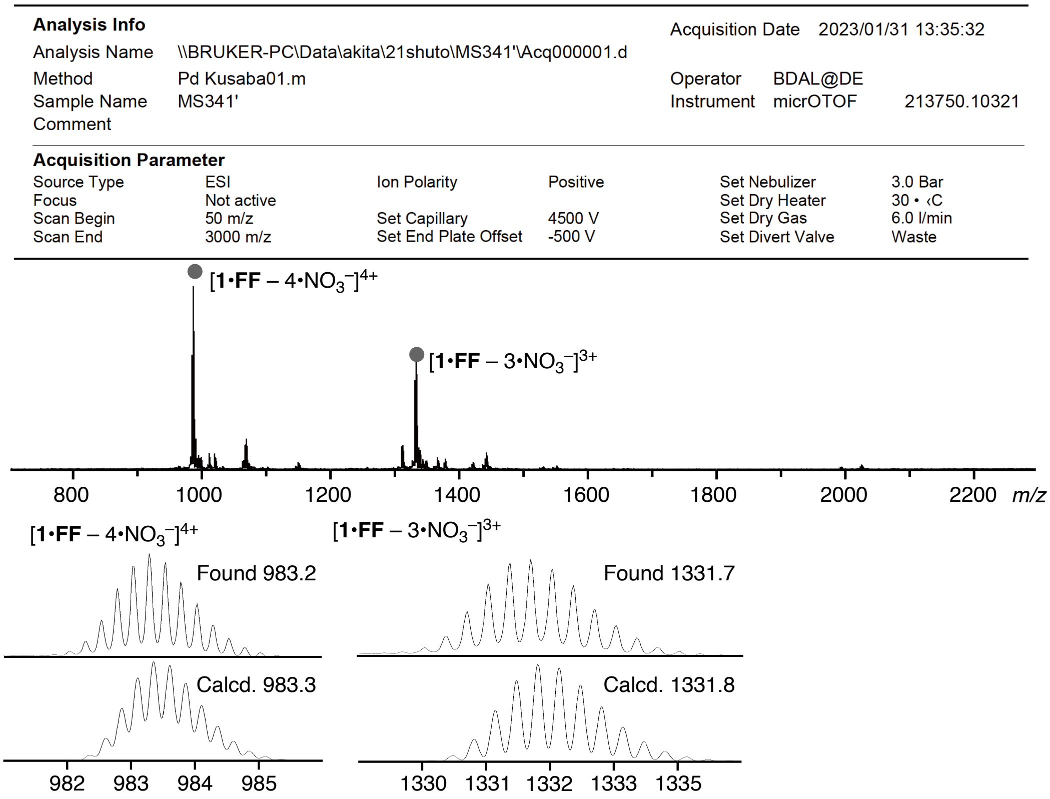

Figure S21b. ESI-TOF MS spectrum (H_2O) of products after mixing **FF** and **YF** with **1** at r.t. for 30 min

Analysis Info

Analysis Name	D:\Data\akita\19sumida\RS1042\1042_PtOMOE3_FF_FY_H2O.d	Acquisition Date	2023/06/12 14:56:57
Method	Pd Kusaba01.m	Operator	BDAL@DE
Sample Name	1042_PtOMOE3_FF_FY_H2O	Instrument	micrOTOF 213750.10321
Comment			

Acquisition Parameter

Source Type	ESI	Ion Polarity	Positive	Set Nebulizer	3.0 Bar
Focus	Not active			Set Dry Heater	30 • ‹C
Scan Begin	50 m/z	Set Capillary	4500 V	Set Dry Gas	6.0 l/min
Scan End	3000 m/z	Set End Plate Offset	-500 V	Set Divert Valve	Waste

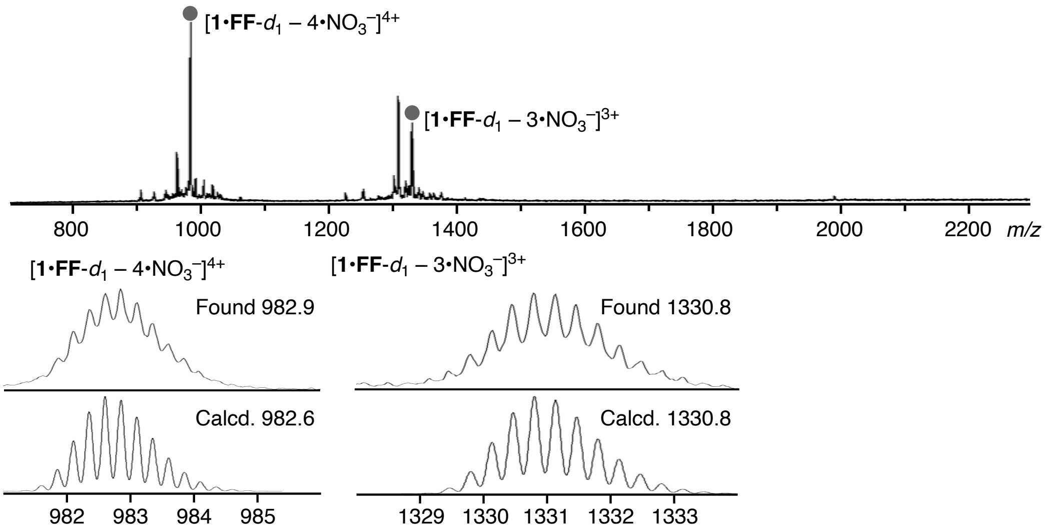

Figure S21c. ESI-TOF MS spectrum (H_2O) of products after mixing **FF** and **FY** with **1** at r.t. for 30 min

Thermodynamic studies of 1•WF MS155, 185

Isothermal titration calorimetry (ITC) measurements were performed by dropping H_2O solutions (6.0 μL each) of **WF** (8.3 mM) to a H_2O solution of **1** (0.19 mM, 1.46 mL) at 25 ºC. In the same way, H_2O solutions (6.0 μL each) of **LF** (6.0 mM) were dropped into a H_2O solution of **1** (0.12 mM, 1.46 mL) at 25 ºC.

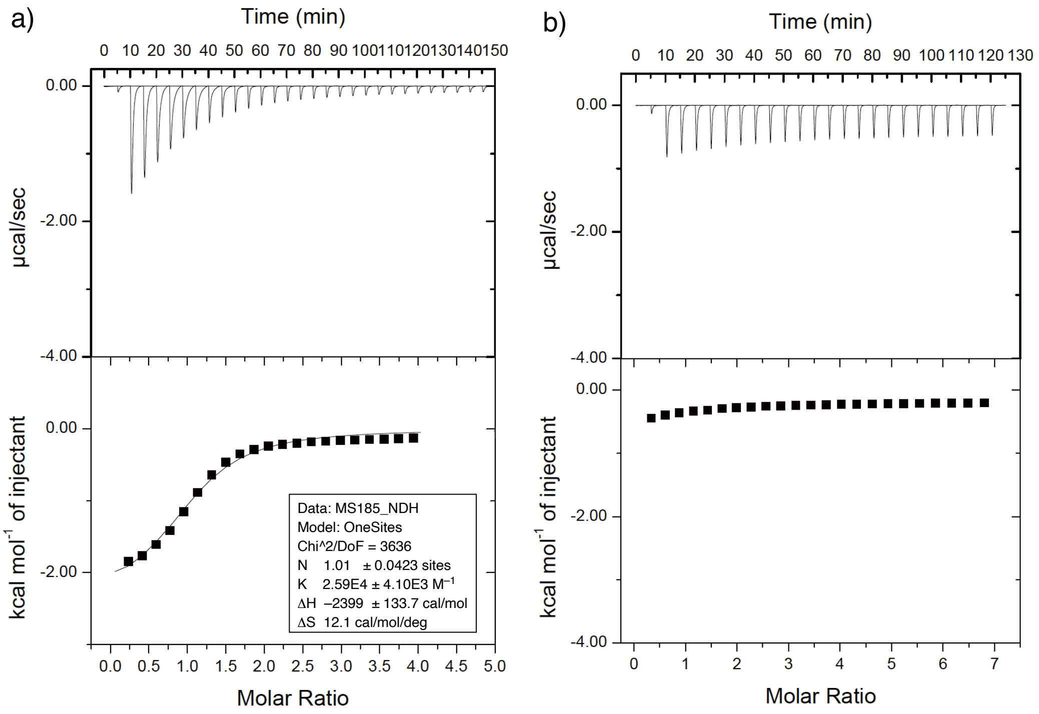

Figure S22. ITC thermographs (H_2O, 25 ºC) and their titration curves of a) **WF** to **1** and b) **LF** to **1**. The solid line represents the best-fitting curve obtained from the "one sites" model.

Table S4. Thermodynamic parameters and binding constants (K_a) for the formation of complexes **1•WF** and **1•FF**, as obtained by ITC experiments (H_2O, 298 K).

complex	ΔH [kJ mol^{-1}]	$T\Delta S$ [kJ mol^{-1}]	ΔG [kJ mol^{-1}]	K_a / 10^5 [M^{-1}]
**1•WF**	−10.0	15.1	−25.1	0.26
**1•FF**	−11.7	17.0	−28.7	1.07

Fluorescent detection of FF using 1 and C153 MS280, 281

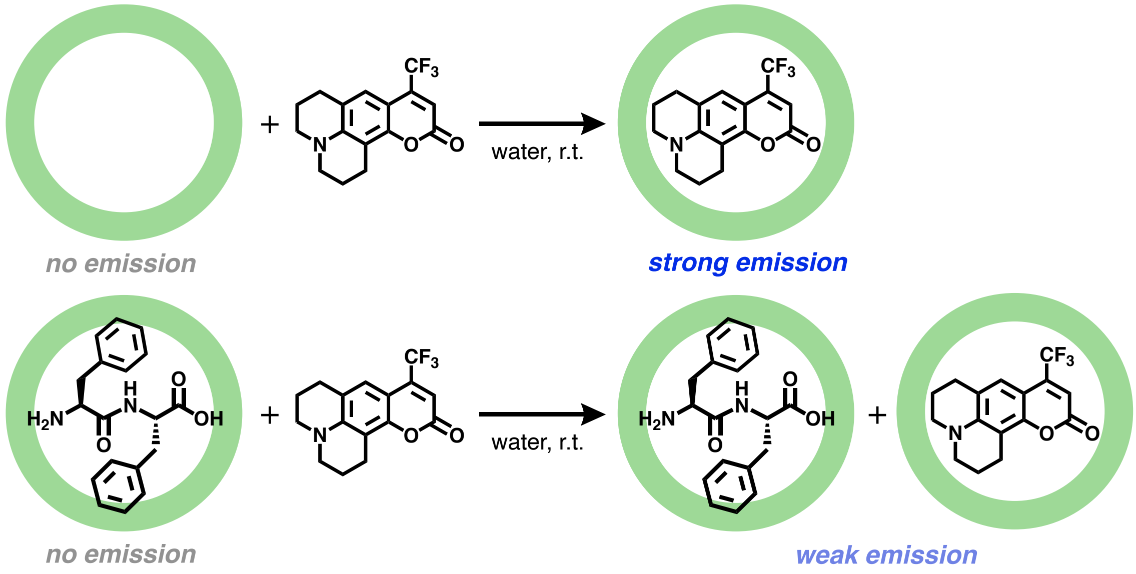

Receptor **1** (0.5 mg, 0.13 µmol) and coumarin 153 (**C153**: 0.08 mg, 0.26 µmol) were added to a 2 mL test tube containing water (0.5 mL). The mixture was stirred at r.t. for 5 min. The resultant solution was analyzed by fluorescence spectroscopy (intensity and quantum yield). The formation of 1:1 host-guest complex **1•C153** was confirmed by NMR analysis. In the same way, receptor **1** (0.5 mg, 0.13 µmol) and **FF** (0.04 mg, 0.13 µmol) were added to a 2 mL test tube containing water (0.5 mL). After addition of **C153** (0.08 mg, 0.26 µmol) to the solution, the mixture was stirred at r.t. for 5 min. The resultant solution was analyzed by fluorescence spectroscopy. The formation of 1:1 host-guest complexes **1•FF** (79%) and **1•C153** (21%) was confirmed by NMR analysis.

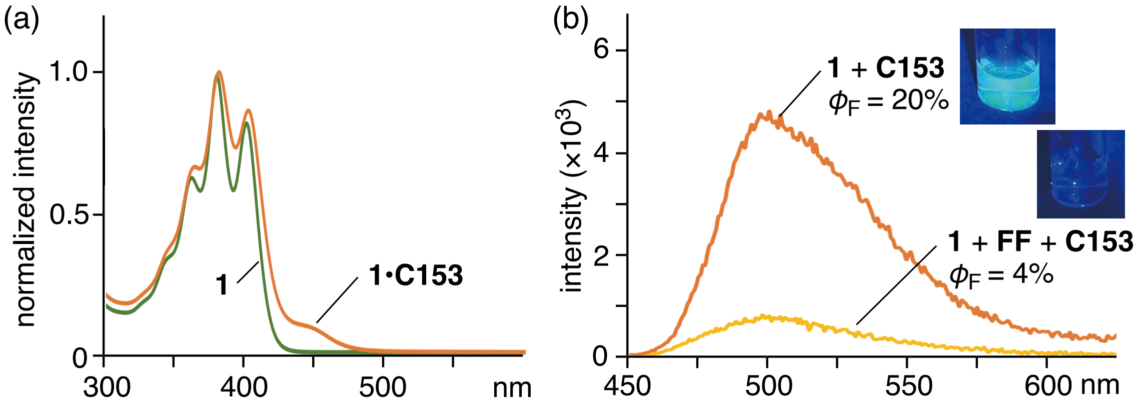

Figure S23a. (a) UV-visible (H_2O, r.t., 0.5 mM based on **1**) of **1** and **1•C153**. (b) Fluorescence spectra and quantum yields (λ_{ex} = 423 nm, H_2O) of **1** + **C153** and **1** + **FF** + **C153**.

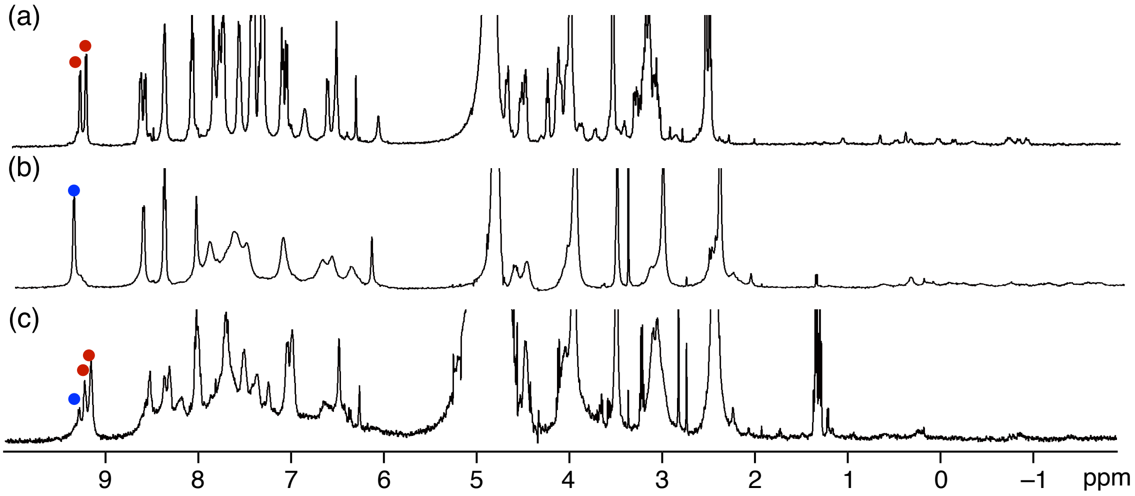

Figure S23b. ^{1}H NMR spectra (500 MHz, D_2O, r.t.) of (a) **1•FF**, (b) **1•C153**, and (c) products after mixing **1**, **FF**, and **C153** at r.t. for 5 min..

Fluorescent detection of FF from mixtures using 1 and C153 MS287, 288

Receptor **1** (0.5 mg, 0.13 μmol), **F** (0.02 mg, 0.13 μmol), **FF** (0.04 mg, 0.13 μmol), and **FFF** (0.06 mg, 0.13 μmol) were added to a 2 mL test tube containing water (0.5 mL). The mixture was stirred at r.t. for 30 min. After addition of **C153** (0.08 mg, 0.26 μmol) to the solution, the mixture was stirred at r.t. for 5 min. The resultant solution was analyzed by fluorescence spectroscopy (intensity and quantum yield). The formation of 1:1 host-guest complex **1•FF** (80%) and **1•C153** (20%) was confirmed by NMR analysis. In the same way, receptor **1** (0.5 mg, 0.13 μmol), **FFF** (0.37 mg, 0.80 μmol), and **F** (0.13 mg, 0.80 μmol) were stirred in water (0.5 mL) at r.t. for 30 min. After addition of **C153** (0.08 mg, 0.26 μmol) to the solution, the mixture was stirred at r.t. for 5 min. The resultant solution was analyzed by fluorescence spectroscopy.

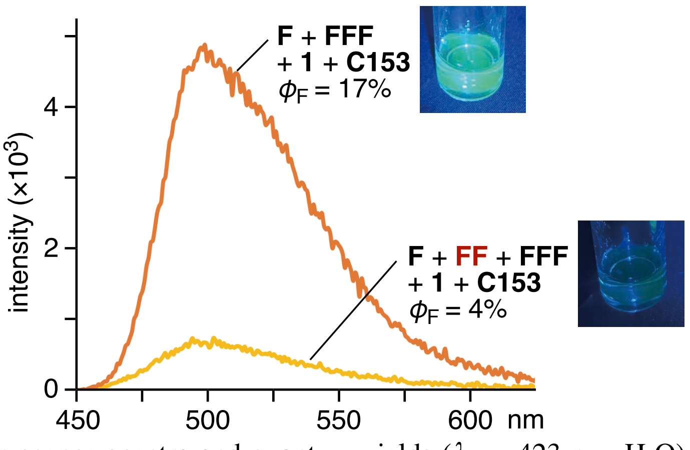

Figure S24a. Fluorescence spectra and quantum yields (λ_{ex} = 423 nm, H_2O) of **1** + **F** + **FFF** + **C153** and **1** + **F** + **FF** + **FFF** + **C153**.

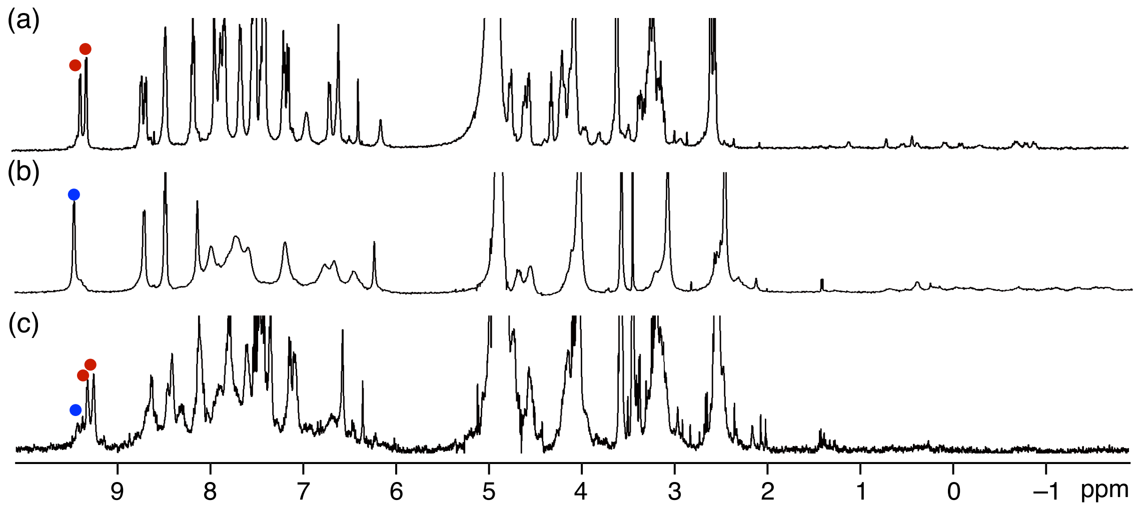

Figure S24b. ^{1}H NMR spectra (500 MHz, D_2O, r.t.) of (a) **1•FF**, (b) **1•C153**, and (c) products after mixing the mixtures of **1**, **F**, **FF**, **FFF**, and **C153** at r.t. for 5 min.

Table S5. Cartesian coordinates of **F** (E_{opt} = –0.114020025738 and E_{sp} = –554.558170521 hartree).

H	-3.52097	2.20193	0.4079	C	1.34086	0.19482	0.45002
C	-3.71298	0.05867	0.22206	C	2.80933	0.28567	0.03454
H	-4.76841	0.05077	0.48733	N	1.22395	-0.93426	1.40063
C	-3.05579	-1.13851	-0.077	O	3.57752	1.20537	0.15927
H	-3.60125	-2.08087	-0.04597	H	0.6858	0.94096	-1.50109
C	-1.70008	-1.13111	-0.41779	H	0.74449	-0.81414	-1.39866
H	-1.19836	-2.06673	-0.65601	H	1.08877	1.14257	1.00783
C	-0.99435	0.08042	-0.46313	H	1.42016	-1.82185	0.94077
C	-1.65576	1.28167	-0.16371	H	0.28027	-0.98359	1.77988
H	-1.11587	2.22627	-0.19883	O	3.21312	-0.87343	-0.5901
C	-3.01046	1.2676	0.17709	H	4.17637	-0.85945	-0.83751
C	0.4653	0.09219	-0.81932				

Table S6. Cartesian coordinates of **FF** (E_{opt} = –0.148137446052 and E_{sp} = –1032.73581825 hartree).

H	5.43302	1.53455	0.55407	C	-2.20272	-0.79022	-0.33618
C	4.22187	1.35472	-1.22374	C	-3.11048	0.37126	0.09876
H	4.65855	2.22681	-1.70621	C	-1.97202	2.25648	1.32403
C	3.22743	0.61715	-1.87596	H	-2.19063	1.75926	2.26705
H	2.89063	0.9162	-2.86778	C	-1.29017	3.47696	1.32795
C	2.66654	-0.50355	-1.26058	H	-0.9789	3.91967	2.27315
H	1.89024	-1.08224	-1.76916	C	-1.00927	4.13052	0.12448
C	3.09894	-0.89479	0.01608	H	-0.4787	5.08054	0.12984
C	4.09691	-0.15557	0.66847	C	-1.41512	3.56108	-1.08716
H	4.44898	-0.45673	1.65212	H	-1.20011	4.06993	-2.02593
C	4.65548	0.96542	0.04685	C	-2.09761	2.34225	-1.09764
C	2.49221	-2.10062	0.67835	H	-2.41482	1.90794	-2.04374
C	1.14081	-1.80258	1.36383	C	-2.37773	1.6836	0.10979
C	0.02351	-1.70112	0.30884	C	-3.04789	-2.08057	-0.48786
N	1.12753	-0.58908	2.2206	O	-4.19085	-2.21236	-0.13036
O	0.09814	-2.27326	-0.78192	O	-2.44335	-3.11809	-1.12208
H	3.1917	-2.53415	1.4204	H	-1.2011	-0.5187	1.54753
H	2.3402	-2.90562	-0.08102	H	-1.73777	-0.55868	-1.33773
H	0.87161	-2.68643	2.01289	H	-3.98755	0.42302	-0.58753
H	1.48433	0.22045	1.70953	H	-3.57054	0.15085	1.08758
H	1.73416	-0.71855	3.027	H	-1.45444	-2.98142	-1.34427
N	-1.13392	-1.02799	0.6604				

Table S7. Cartesian coordinates of **FFF** (E_{opt} = –0.194621225247 and E_{sp} = –1550.19477693 hartree).

H	8.06825	-2.98174	0.14406	H	4.85705	1.11557	-1.70896
C	8.87751	-0.99003	-0.06749	H	4.03333	-0.60362	0.75779
H	9.89033	-1.2881	0.19689	H	4.64802	2.30072	0.51265
C	8.59802	0.34286	-0.38307	H	5.28249	1.2072	1.55039
H	9.39586	1.08425	-0.36672	H	2.02978	1.41991	1.6094
C	7.29783	0.72803	-0.72315	H	0.17477	1.65985	-0.71754
H	7.0927	1.76528	-0.97753	H	-0.48679	2.11441	2.28439
C	6.26805	-0.22417	-0.75111	H	0.42767	3.31382	1.35305
C	6.55121	-1.56266	-0.43717	N	-0.37092	-0.89698	-0.39428
H	5.75936	-2.30968	-0.46461	C	-1.09904	-2.17837	-0.51526
C	7.85207	-1.94129	-0.09702	C	-0.88106	-3.13905	0.67645

C	4.86718	0.18579	-1.10466	O	-1.14992	-2.73672	1.9363
C	4.00369	0.35486	0.16251	O	-0.49741	-4.27286	0.50776
C	2.53569	0.56616	-0.25258	C	-2.59284	-2.01394	-0.84872
N	4.43104	1.46521	1.05214	C	-3.50567	-1.6333	0.32191
O	2.09562	0.20227	-1.3402	C	-5.30324	-0.2227	-0.74159
N	1.68422	1.12045	0.69435	H	-4.57886	0.58443	-0.84232
C	0.26221	1.36848	0.37075	C	-6.61372	-0.04376	-1.19084
C	-0.3121	2.49235	1.24971	H	-6.90757	0.90242	-1.64177
C	-1.57755	4.07925	-0.24911	C	-7.54888	-1.07624	-1.06383
H	-0.63251	4.52515	-0.55164	H	-8.56896	-0.93465	-1.41329
C	-2.7765	4.5684	-0.77576	C	-7.16775	-2.29103	-0.48564
H	-2.75895	5.39255	-1.48727	H	-7.8933	-3.09674	-0.385
C	-3.99682	4.00481	-0.39005	C	-5.85759	-2.4761	-0.03543
H	-4.92888	4.39081	-0.79813	H	-5.56793	-3.42476	0.41313
C	-4.01734	2.9457	0.52364	C	-4.91883	-1.44116	-0.15991
H	-4.96721	2.50598	0.82828	H	0.30746	-0.71303	-1.15979
C	-2.82243	2.45085	1.05093	H	-0.62475	-2.70897	-1.40081
H	-2.83538	1.61724	1.75838	H	-1.43982	-1.75338	2.04091
C	-1.59799	3.01965	0.66946	H	-2.95607	-2.96773	-1.29169
C	-0.5227	0.04674	0.60192	H	-2.69651	-1.25678	-1.6556
O	-1.21403	-0.13236	1.60459	H	-3.46637	-2.41306	1.11256
H	4.39339	-0.56889	-1.77581	H	-3.1435	-0.70253	0.81415

Table S8. Cartesian coordinates of **1** (R = OCH_3; E_{opt} = 1.78672633577 and E_{sp} = –8820.29086971 hartree).

N	-7.32485	1.08089	-1.52268	C	1.31542	4.98255	5.03851
C	-6.17457	1.66258	-1.98753	C	0.09015	4.60432	4.47897
H	-5.25152	1.49887	-1.41166	H	0.07645	3.83304	3.70258
C	-6.17387	2.44336	-3.15893	C	4.78187	-3.14792	-3.79795
C	-7.38761	2.61707	-3.85436	C	-3.1653	-0.19773	5.56159
H	-7.40567	3.22206	-4.77223	H	-3.32967	0.86217	5.77315
C	-8.55227	2.02019	-3.36968	C	6.16457	-2.10371	1.59652
H	-9.50581	2.14654	-3.8989	H	5.24997	-1.51269	1.43946
C	-8.50358	1.25354	-2.19835	C	-6.15575	-3.29355	-2.35287
H	-9.3989	0.76711	-1.78557	C	-4.06816	-0.92198	4.83962
N	-7.31284	-1.68469	-0.96348	H	-4.97972	-0.45343	4.47824
N	-7.26221	-1.12667	1.80262	C	3.88459	4.42443	-2.42399
C	-8.41704	-1.31017	2.51574	C	2.67847	4.91775	-3.01819
H	-9.3288	-0.82896	2.13407	C	1.79159	5.72044	-2.20715
C	-8.42194	-2.08083	3.68539	H	0.90021	6.13943	-2.67978
H	-9.35682	-2.2162	4.24486	C	2.06231	5.97004	-0.89486
C	-7.23756	-2.66992	4.13033	H	1.398	6.58007	-0.28433
H	-7.22116	-3.27742	5.04662	C	3.25382	5.44537	-0.29367
C	-6.0483	-2.48403	3.3969	H	3.44081	5.66154	0.76164
C	-6.093	-1.70065	2.22816	C	4.13418	4.70801	-1.03001
H	-5.19046	-1.52831	1.62303	H	5.04971	4.33826	-0.57587
C	4.91257	-3.70937	3.05205	C	5.67812	-2.69543	5.20101
N	7.26217	-1.15108	-1.78713	C	4.92816	3.11681	3.59952
C	8.41701	-1.34432	-2.49765	C	-2.6448	-2.93407	5.04091
H	9.32878	-0.85799	-2.12256	C	-3.99948	2.29983	-4.37685
C	8.42192	-2.13085	-3.65669	C	-8.4812	-2.38273	-1.11669
H	9.35681	-2.27385	-4.21424	H	-9.37515	-1.98922	-0.61219
C	7.23753	-2.7259	-4.0936	C	-2.0621	5.98129	0.81357

H	7.22113	-3.34584	-5.00152	H	-1.39773	6.58285	0.19475
C	6.04824	-2.52998	-3.36281	C	-3.28909	6.45749	-3.61096
C	6.09294	-1.73075	-2.20485	H	-2.37218	6.92157	-3.98173
H	5.19039	-1.55013	-1.60216	C	2.38496	-4.35951	-4.70069
C	-4.13407	4.7214	0.96595	C	-4.91266	-3.75042	-3.00139
H	-5.04964	4.34557	0.51692	C	-8.45226	3.504	2.2143
C	-3.306	3.95894	5.10123	H	-9.39526	4.04542	2.36573
C	-4.53222	3.49161	4.52681	C	-2.14271	0.80549	-5.9153
C	-5.46607	2.78405	5.37073	H	-1.46308	0.20662	-6.51999
H	-6.40413	2.44167	4.94147	C	-1.90707	2.12733	-5.68051
C	-5.19811	2.56909	6.69163	H	-1.02895	2.61934	-6.10544
H	-5.91272	2.05669	7.33979	C	-1.31319	-5.15219	-4.91908
C	-3.96252	3.01744	7.26518	C	-0.09126	-4.56864	-4.56783
H	-3.78079	2.82628	8.32416	H	-0.07766	-3.78118	-3.8079
C	-3.0526	3.68066	6.49689	C	1.11438	-4.97419	-5.14565
H	-2.11311	4.03221	6.93002	C	1.09523	-5.96701	-6.16637
N	7.31279	-1.67153	0.98629	C	-0.13307	-6.55409	-6.55133
C	-3.8845	4.45686	2.36368	C	-1.32587	-6.18888	-5.88856
C	-2.39235	4.69398	4.31061	C	3.49966	5.10558	3.81315
C	-6.16463	-2.12513	-1.5678	C	4.70143	4.48653	3.3394
H	-5.25003	-1.53203	-1.41882	C	5.65025	5.28963	2.60483
C	4.70979	-3.43011	4.42168	H	6.56877	4.82583	2.25474
C	-2.67835	4.95818	2.95109	C	5.42068	6.615	2.37344
C	-6.10286	2.0913	1.79453	H	6.14663	7.23194	1.83898
H	-5.19207	1.50326	1.60712	C	4.21257	7.23501	2.83439
C	-4.14792	-4.76493	-0.85196	H	4.06332	8.29688	2.63253
H	-5.0447	-4.4005	-0.35785	C	3.28926	6.50591	3.52292
C	-3.31973	-5.06282	4.02672	H	2.37236	6.97502	3.88734
C	-3.96272	-4.48161	-2.25591	C	-5.25046	-6.6051	2.63645
C	-4.54148	-4.46321	3.57981	H	-5.98	-7.23522	2.1227
C	3.51279	-3.88333	5.06479	C	-1.85202	-5.76136	-2.13109
C	-1.31532	4.91365	-5.10569	H	-0.97767	-6.17136	-2.64216
C	-0.09005	4.54308	-4.54106	C	-2.81412	2.92265	-4.88488
H	-0.07634	3.78246	-3.75422	C	-7.35877	-4.01176	-2.50714
C	1.11833	5.10227	-4.96453	H	-7.37026	-4.92751	-3.11553
C	1.09949	6.12583	-5.95463	C	-5.4205	6.58225	-2.46304
C	-0.13105	6.52803	-6.52501	H	-6.14644	7.20643	-1.93702
C	-1.32786	5.88191	-6.14365	C	-1.73397	-2.13535	5.82899
C	-2.56096	-4.69059	-4.2718	H	-0.84596	-2.62181	6.23924
N	-7.27404	1.64118	1.24376	C	-2.06194	-6.01048	-0.80758
C	2.56086	-4.63219	4.33507	H	-1.36552	-6.61296	-0.22596
C	-4.70131	4.44083	-3.39996	C	1.31309	-5.08499	4.98854
C	-4.79735	3.73271	3.16054	C	0.09114	-4.50626	4.6294
C	-4.02007	-7.20401	3.06562	H	0.07753	-3.72919	3.85886
H	-3.85744	-8.26333	2.85981	C	-1.11448	-4.90396	5.21269
C	3.96263	-4.45058	2.31654	C	-1.09535	-5.88285	6.24677
C	4.14788	-4.75296	0.91657	C	0.13297	-6.46466	6.63962
H	5.0447	-4.39531	0.4176	C	1.32575	-6.10836	5.97201
C	3.22988	-5.492	0.22939	C	7.3587	-3.97739	2.56146
H	3.36782	-5.72217	-0.83047	H	7.37017	-4.88477	3.18227
C	2.06183	-5.99887	0.88903	C	8.52126	-3.52623	1.93488
H	1.36537	-6.60912	0.31561	H	9.46648	-4.07205	2.053
C	1.85187	-5.7318	2.20903	C	-4.21241	0.89694	-4.64584

H	0.97747	-6.13475	2.72558	H	-5.11359	0.4218	-4.26719
C	2.78184	-4.92933	2.97027	C	-3.31596	0.17766	-5.38076
N	7.3249	1.10139	1.50791	H	-3.47611	-0.8841	-5.58695
C	6.17464	1.68937	1.96481	C	5.47035	-2.44431	6.52639
H	5.25159	1.51792	1.39119	C	3.99955	2.35902	4.34529
C	6.17394	2.48595	3.12553	C	4.21244	0.95991	4.63331
C	7.38768	2.66901	3.81856	H	5.11359	0.47965	4.26112
H	7.40575	3.28638	4.72816	C	3.31598	0.25071	5.37795
C	8.55232	2.06557	3.34203	H	3.4761	-0.80815	5.59853
H	9.50586	2.19905	3.86952	C	2.14276	0.88579	5.90394
C	8.50363	1.28312	2.18121	H	1.46314	0.29522	6.51674
H	9.39893	0.79111	1.77506	C	1.90716	2.20432	5.65121
C	6.15568	-3.26135	2.39741	H	1.02906	2.70209	6.06943
C	-2.56743	4.29035	-4.62306	C	2.81421	2.98872	4.84482
C	-6.0662	3.26337	2.57346	C	4.26758	-2.88573	7.17057
C	-4.92807	3.0677	-3.64144	H	4.13672	-2.66939	8.23202
C	3.3269	-3.57485	6.46462	C	-3.84681	-2.32155	4.56038
H	2.41423	-3.92574	6.95202	C	-4.78193	-3.09604	3.84037
C	-3.09181	-6.45901	3.72999	C	3.84672	-2.38322	-4.52831
H	-2.15656	-6.91142	4.06841	C	4.06806	-0.98755	-4.8264
C	-5.49585	-5.28325	2.87155	H	4.97966	-0.51418	-4.47146
H	-6.43067	-4.83493	2.54522	C	3.16515	-0.27309	-5.558
N	7.27407	1.62409	-1.26588	H	3.32952	0.78387	-5.78382
C	8.439	2.31221	-1.47854	C	1.97832	-0.89957	-6.06329
H	9.35204	1.91745	-1.01044	H	1.29635	-0.30609	-6.67072
C	8.45229	3.47363	-2.2615	C	1.73383	-2.21414	-5.79921
H	9.39527	4.01298	-2.42021	H	0.84581	-2.7061	-6.20286
C	7.26596	3.93606	-2.83284	C	2.6447	-3.00217	-5.00049
H	7.25605	4.84647	-3.44923	H	6.59326	-2.36154	4.71903
C	6.06625	3.2281	-2.61747	H	6.21052	-1.90906	7.12542
C	6.10291	2.06668	-1.82274	O	2.56472	-6.60234	6.2583
H	5.19212	1.4812	-1.62743	O	-2.32567	-6.15297	6.74452
C	-2.78195	-4.96922	-2.90311	O	2.56914	6.26778	6.54152
C	4.7974	3.6894	-3.21089	O	-2.33229	6.68896	6.15607
C	4.53224	3.42967	-4.57374	O	2.33243	6.60456	-6.24647
C	5.46604	2.71061	-5.40794	O	-2.56906	6.17833	-6.62601
H	6.40411	2.37411	-4.97406	O	2.32554	-6.24384	-6.66043
C	5.19803	2.47762	-6.72577	O	-2.56486	-6.68671	-6.16765
H	5.91261	1.95638	-7.36687	C	2.49477	-7.30094	-7.65122
C	3.96245	2.91814	-7.30536	H	2.11759	-6.95411	-8.61689
H	3.78069	2.71253	-8.36162	H	3.58729	-7.4127	-7.65726
C	3.05258	3.59185	-6.54619	H	1.99045	-8.22088	-7.33378
H	2.1131	3.93752	-6.98406	C	-2.70522	-8.12055	-6.40724
C	3.30603	3.88916	-5.15446	H	-1.85705	-8.54879	-6.95351
C	-3.25365	5.44855	0.2196	H	-2.82863	-8.59513	-5.42861
H	-3.44062	5.65031	-0.83856	H	-3.6295	-8.1651	-6.99696
C	-3.22993	-5.49461	-0.15484	C	2.70491	-8.03316	6.51543
H	-3.36783	-5.71034	0.90806	H	2.82721	-8.52002	5.54273
C	-3.51287	-3.95164	-5.01159	H	3.62977	-8.07059	7.10476
C	-3.32695	-3.66211	-6.41546	H	1.85715	-8.45419	7.06791
H	-2.41425	-4.01953	-6.89804	C	-2.49478	-7.19618	7.74995
C	-4.26762	-2.98261	-7.13069	H	-1.99087	-8.12056	7.44503
H	-4.13674	-2.78063	-8.19495	H	-2.11709	-6.83616	8.71058

C	-5.47043	-2.53255	-6.49256	H	-3.58734	-7.30752	7.75798
H	-6.21059	-2.00547	-7.09879	C	2.72411	6.52554	7.97069
C	-5.67821	-2.76573	-5.16391	H	2.86613	5.55417	8.45484
H	-6.59339	-2.42537	-4.68652	H	3.64116	7.12756	7.99688
C	-4.70988	-3.48976	-4.37469	H	1.87429	7.06585	8.40319
C	2.56753	4.35274	4.56442	C	-2.50065	7.69403	7.19969
C	-3.49953	5.05335	-3.88209	H	-3.59173	7.68863	7.3249
C	2.39243	4.63499	-4.37396	H	-1.98127	7.39705	8.11808
C	-7.26593	3.97415	2.7793	H	-2.13961	8.65866	6.83335
H	-7.25602	4.8928	3.38332	C	2.50085	7.59564	-7.30337
C	-8.52133	-3.55211	-1.88675	H	3.59196	7.58874	-7.42827
H	-9.46655	-4.09949	-1.99742	H	1.98173	7.28632	-8.21782
C	8.48114	-2.36743	1.14899	H	2.13958	8.56503	-6.95005
H	9.3751	-1.98083	0.63918	C	-2.72412	6.41642	-8.05859
C	-2.38506	-4.29533	4.75945	H	-1.87389	6.94985	-8.49876
C	4.54142	-4.51146	-3.51895	H	-2.86717	5.43853	-8.52912
C	3.31964	-5.11702	-3.95766	H	-3.6406	7.01889	-8.09298
C	3.09169	-6.50906	-3.64202	O	-0.13414	-7.53565	-7.53336
H	2.15639	-6.96598	-3.97422	O	0.13401	-7.43297	7.63469
C	4.01996	-7.24503	-2.96767	O	-0.13162	7.51	-7.50661
H	3.85729	-8.30145	-2.74751	O	0.1317	7.61144	7.40376
C	5.25039	-6.64041	-2.54673	C	0.65317	-6.94737	8.91103
H	5.97995	-7.26355	-2.02455	H	0.5799	-7.84568	9.5384
C	5.4958	-5.32187	-2.79971	H	1.69199	-6.62372	8.8009
H	6.43065	-4.86921	-2.47953	H	0.02152	-6.14251	9.29344
C	-1.9785	-0.81735	6.07535	C	-0.6288	8.79922	-7.03219
H	-1.29658	-0.21575	6.6748	H	-0.55866	9.41347	-7.93978
C	-8.43897	2.33209	1.44713	H	-1.6649	8.70662	-6.69462
H	-9.35201	1.93103	0.98443	H	0.01737	9.18437	-6.24029
C	-5.6501	5.25386	-2.67639	C	0.6285	8.89414	6.91162
H	-6.56863	4.79488	-2.32	H	0.55828	9.5208	7.81067
C	-1.79141	5.74965	2.12916	H	1.66459	8.79717	6.57527
H	-0.89999	6.175	2.59601	H	-0.01783	9.26821	6.11454
C	-4.21238	7.1959	-2.9324	C	-0.65233	-7.06682	-8.81635
H	-4.06311	8.26043	-2.74501	H	-0.57884	-7.97335	-9.43176
C	-1.11823	5.16924	4.89477	H	-1.69115	-6.74155	-8.71117
C	-1.09939	6.2062	5.87082	H	-0.02028	-6.26721	-9.20894
C	0.13114	6.61614	6.43567	Pt	7.29111	-0.02413	-0.1402
C	1.32795	5.96487	6.06319	Pt	-7.29112	-0.02216	0.14054

Table S9. Cartesian coordinates of **1•F** (R = OCH_3; E_{opt} = 1.64536525368 and E_{sp} = –9374.87919357 hartree).

H	2.06066	-1.65449	-2.98965	H	-1.20397	0.30784	6.25969
C	2.33659	-2.79014	-1.16902	C	-8.76201	1.52174	1.10911
H	3.12968	-3.41329	-1.58457	H	-9.60855	1.01499	0.62424
C	1.91279	-2.98017	0.14935	C	-6.21563	4.53341	-3.06028
H	2.38188	-3.74775	0.76885	H	-7.09089	3.98688	-2.71714
C	0.8851	-2.19467	0.67966	C	-2.47375	5.5078	1.67415
H	0.56357	-2.34916	1.70729	H	-1.63311	6.05096	2.11314
C	0.27602	-1.21303	-0.11384	C	-4.99471	6.61368	-3.35219
C	0.70485	-1.01893	-1.4362	H	-4.97159	7.69504	-3.20511
H	0.23882	-0.25645	-2.05834	C	-1.85764	5.29916	4.51558

C	1.73225	-1.80677	-1.95923	C	-2.02522	6.41809	5.3903
C	-0.84045	-0.37193	0.43719	C	-0.90277	6.96347	6.04225
C	-2.20564	-0.96619	0.04362	C	0.39642	6.43888	5.78244
C	-3.32925	0.07234	0.14802	C	0.57297	5.4176	4.8255
N	-2.66261	-2.10295	0.87487	C	-0.57364	4.84921	4.23393
O	-4.52658	-0.10405	-0.02544	H	-0.44347	4.02173	3.53011
H	-0.75253	0.67452	0.06144	C	5.35236	-2.7934	-3.61616
H	-0.76203	-0.26294	1.53928	C	-3.0543	-0.01292	5.16351
H	-2.17541	-1.28155	-1.04253	H	-3.34655	1.0358	5.26492
H	-2.31444	-2.04013	1.8324	C	6.39835	-1.27748	1.80072
H	-2.34247	-2.9936	0.48776	H	5.40279	-0.84447	1.62094
O	-2.8672	1.31164	0.43317	C	-5.53	-3.96079	-2.24252
H	-3.56499	2.0373	0.48465	C	-3.85837	-0.91253	4.52914
Pt	-7.24049	-0.75245	-0.02355	H	-4.81597	-0.59711	4.12051
Pt	7.2117	0.85893	-0.05755	C	3.32588	4.8043	-2.57666
N	-7.41989	0.25278	-1.73819	C	2.09718	5.17454	-3.21254
C	-6.33949	0.94598	-2.21287	C	1.12395	5.92314	-2.45056
H	-5.41774	0.91307	-1.60435	H	0.20881	6.24172	-2.95795
C	-6.39844	1.66213	-3.42206	C	1.34016	6.23798	-1.14234
C	-7.60355	1.65305	-4.14963	H	0.61082	6.80807	-0.56749
H	-7.67221	2.20251	-5.09837	C	2.55695	5.83683	-0.4983
C	-8.70111	0.94419	-3.65472	H	2.6971	6.10038	0.55357
H	-9.64801	0.92851	-4.2089	C	3.51601	5.1549	-1.189
C	-8.59359	0.24824	-2.44417	H	4.44682	4.87784	-0.70138
H	-9.43374	-0.32129	-2.02241	C	5.90021	-1.52207	5.35609
N	-6.97118	-2.44328	-1.03599	C	4.37259	3.89768	3.45891
N	-7.0619	-1.76415	1.68294	C	-2.22281	-2.71806	4.96002
C	-8.17643	-2.05788	2.42537	C	-4.17237	1.71819	-4.55538
H	-9.14651	-1.72481	2.02928	C	-8.0397	-3.27461	-1.24747
C	-8.06507	-2.75161	3.63475	H	-9.01091	-2.95787	-0.84003
H	-8.9655	-2.98015	4.21806	C	-2.72557	5.58188	0.33605
C	-6.80498	-3.14669	4.09154	H	-2.09538	6.17135	-0.32867
H	-6.69981	-3.68723	5.04172	C	-3.97203	5.95972	-3.97166
C	-5.66155	-2.8489	3.32617	H	-3.10119	6.51131	-4.33467
C	-5.82233	-2.15419	2.11305	C	3.15611	-4.38567	-4.43515
H	-4.95647	-1.89987	1.46344	C	-4.20699	-4.30558	-2.80082
C	5.41198	-2.95601	3.37127	C	-8.91899	2.72532	1.81013
N	7.40884	-0.3364	-1.64353	H	-9.91449	3.18021	1.89106
C	8.60732	-0.37813	-2.3071	C	-2.08838	0.3807	-5.94222
H	9.41637	0.26307	-1.92963	H	-1.32228	-0.16234	-6.49353
C	8.78101	-1.20696	-3.42207	C	-2.02788	1.73299	-5.78631
H	9.74794	-1.22653	-3.94209	H	-1.20784	2.30734	-6.22328
C	7.72231	-2.00273	-3.86383	C	-0.44392	-5.54014	-4.51324
H	7.83903	-2.66001	-4.7378	C	0.72673	-4.84697	-4.20408
C	6.49047	-1.96323	-3.18158	H	0.68055	-4.01262	-3.49543
C	6.36247	-1.1123	-2.06629	C	1.9563	-5.17384	-4.78622
H	5.41835	-1.04931	-1.50345	C	2.01637	-6.24678	-5.71817
C	-4.69573	4.1805	0.57973	C	0.86337	-7.0268	-5.97224
H	-5.5853	3.71652	0.15892	C	-0.3748	-6.65456	-5.40075
C	-3.8909	3.86436	4.77557	C	2.73394	5.72582	3.59377
C	-5.059	3.23884	4.2298	C	4.00133	5.22551	3.15126
C	-5.925	2.49601	5.11456	C	4.86763	6.10595	2.40361
H	-6.82091	2.03579	4.70374	H	5.83722	5.73571	2.07982

C	-5.64553	2.38862	6.44564	C	4.49593	7.38901	2.12294
H	-6.30699	1.84275	7.12257	H	5.15854	8.06599	1.57807
C	-4.46943	3.0013	6.9935	C	3.21594	7.88349	2.54074
H	-4.27959	2.89728	8.06354	H	2.94863	8.91264	2.29193
C	-3.62779	3.70949	6.18921	C	2.36815	7.07944	3.24235
H	-2.73852	4.19012	6.60458	H	1.39061	7.44952	3.56418
N	7.46446	-0.71007	1.15383	C	-4.3833	-6.90737	3.00389
C	-4.4619	4.07679	2.00159	H	-5.03116	-7.66881	2.56356
C	-3.04257	4.62532	3.93886	C	-0.99997	-5.93228	-1.68745
C	-5.73076	-2.77407	-1.51337	H	-0.07247	-6.29191	-2.13877
H	-4.90674	-2.07091	-1.28923	C	-3.05182	2.45229	-5.06295
C	5.09244	-2.50308	4.67056	C	-6.62675	-4.8202	-2.45142
C	-3.31449	4.73082	2.55515	H	-6.48554	-5.75581	-3.00954
C	-6.44324	1.51706	1.59346	C	-6.14335	5.88593	-2.89525
H	-5.48221	0.98275	1.4967	H	-6.95295	6.44414	-2.41964
C	-3.42515	-5.07309	-0.55766	C	-1.41349	-1.73351	5.6422
H	-4.37231	-4.76138	-0.121	H	-0.48098	-2.06628	6.10311
C	-2.65212	-5.01151	4.21034	C	-1.23271	-6.10011	-0.35519
C	-3.21715	-4.87658	-1.97401	H	-0.50397	-6.58699	0.29125
C	-3.92496	-4.60515	3.69253	C	1.91412	-4.50533	5.35389
C	3.94482	-3.03596	5.34149	C	0.67417	-4.08703	4.86183
C	-1.80574	4.60161	-5.37106	H	0.6469	-3.426	3.9917
C	-0.55702	4.43677	-4.78456	C	-0.52823	-4.49866	5.43883
H	-0.44417	3.71543	-3.97005	C	-0.48887	-5.33419	6.59441
C	0.57632	5.16444	-5.20135	C	0.75395	-5.74875	7.12606
C	0.41425	6.1387	-6.20775	C	1.95387	-5.38824	6.46903
C	-0.84292	6.29402	-6.86137	C	7.86755	-2.89269	2.85175
C	-1.94327	5.50924	-6.46746	H	8.01999	-3.75101	3.52217
C	-1.73422	-5.11385	-3.92805	C	8.94515	-2.30764	2.18407
N	-7.53112	0.93279	1.00367	H	9.96272	-2.69799	2.31843
C	3.15563	-4.0249	4.70964	C	-4.19824	0.28539	-4.74051
C	-5.15568	3.80469	-3.71702	H	-5.04893	-0.27488	-4.35954
C	-5.32897	3.3545	2.84934	C	-3.19219	-0.35727	-5.39948
C	-3.10112	-7.31385	3.50473	H	-3.20917	-1.44086	-5.54099
H	-2.82143	-8.36567	3.42266	C	5.59942	-1.12813	6.62784
C	4.66751	-3.9899	2.76422	C	3.5158	3.06571	4.21251
C	5.02423	-4.53414	1.47495	C	3.85977	1.69924	4.52679
H	5.8805	-4.11745	0.95041	H	4.80187	1.29725	4.16278
C	4.32451	-5.57299	0.93368	C	3.03153	0.92177	5.28223
H	4.603	-6.00002	-0.033	H	3.28779	-0.1153	5.51526
C	3.20381	-6.1389	1.62784	C	1.80707	1.4539	5.80504
H	2.68573	-6.98482	1.17625	H	1.1903	0.81488	6.43619
C	2.82154	-5.63446	2.83512	C	1.44803	2.73906	5.52824
H	1.98074	-6.06852	3.38263	H	0.5284	3.16226	5.94207
C	3.53242	-4.53502	3.44642	C	2.27299	3.58634	4.69749
N	7.04001	2.06315	1.5272	C	4.44708	-1.65494	7.30002
C	5.81705	2.53601	1.92523	H	4.24066	-1.32018	8.31788
H	4.93562	2.22173	1.34638	C	-3.47968	-2.30255	4.41219
C	5.69272	3.3933	3.03545	C	-4.32181	-3.25456	3.79962
C	6.85867	3.75937	3.73809	C	4.3494	-2.22877	-4.43314
H	6.78268	4.42597	4.60834	C	4.39158	-0.84769	-4.85254
C	8.09834	3.27176	3.32238	H	5.22267	-0.2275	-4.52758
H	9.01573	3.54776	3.85908	C	3.42429	-0.32977	-5.66385

C	8.17127	2.42285	2.21106	H	3.4549	0.71437	-5.98545
H	9.12861	2.01737	1.85255	C	2.34511	-1.15145	-6.12829
C	6.57063	-2.37499	2.66659	H	1.6105	-0.71317	-6.80319
C	-2.97931	3.85054	-4.87102	C	2.26758	-2.4577	-5.74647
C	-6.54406	2.73642	2.28426	H	1.46479	-3.10072	-6.11737
C	-5.21508	2.40542	-3.89851	C	3.25121	-3.04071	-4.86301
C	3.64761	-2.56541	6.6751	H	6.77903	-1.12586	4.85415
H	2.77203	-2.97729	7.18237	H	6.22638	-0.41182	7.16334
C	-2.26898	-6.39978	4.07783	O	3.21029	-5.78787	6.77855
H	-1.29442	-6.70343	4.46662	O	0.79334	-6.57876	8.23845
C	-4.77293	-5.60254	3.08143	O	-1.7226	-5.6448	7.05687
H	-5.74322	-5.29702	2.69556	O	1.34807	6.99217	-6.71031
N	6.98768	2.43211	-1.26717	O	-1.06326	7.30293	-7.77762
C	8.07017	3.23711	-1.50715	O	-3.19297	5.52779	-6.99012
H	9.01682	2.97013	-1.01538	O	1.3497	7.06843	6.5228
C	7.96195	4.35398	-2.34457	O	-1.12379	7.89595	7.03586
H	8.84034	4.9875	-2.52572	O	-3.31693	6.80499	5.5186
C	6.73619	4.65237	-2.94137	O	3.24308	-6.3958	-6.27308
H	6.63234	5.52572	-3.60019	O	0.9307	-8.10058	-6.85012
C	5.6206	3.82603	-2.69729	O	-1.57865	-7.24166	-5.59695
C	5.77956	2.71279	-1.84949	C	2.57058	7.26688	-5.97923
H	4.9389	2.03659	-1.63274	C	-0.22942	7.26416	-8.9714
C	-1.97308	-5.29688	-2.54662	C	-3.49067	6.40542	-8.11736
C	4.31732	4.12693	-3.31978	C	2.65	6.45724	6.72718
C	4.09676	3.78301	-4.6722	C	-0.42694	9.16524	6.877
C	5.10018	3.10857	-5.46124	C	-3.64988	7.932	6.3842
H	6.05524	2.86943	-5.00024	C	3.42266	-6.87435	7.72928
C	4.87227	2.79289	-6.76955	C	0.90761	-5.8514	9.4987
H	5.63783	2.30405	-7.37723	C	-1.89064	-6.65169	8.09855
C	3.61309	3.09994	-7.38374	C	3.42644	-7.27269	-7.425
H	3.46351	2.82649	-8.4302	C	1.26654	-9.36118	-6.19565
C	2.63999	3.73263	-6.66932	C	-1.74427	-8.26421	-6.62362
H	1.67741	3.9718	-7.13032	H	2.88151	8.21848	-6.44043
C	2.84895	4.11619	-5.29153	H	3.29957	6.47828	-6.18386
C	-3.85844	4.90297	-0.22056	H	2.37857	7.40181	-4.91245
H	-4.04193	4.9942	-1.29563	H	-0.34912	6.31443	-9.49404
C	-2.46953	-5.65775	0.22	H	0.81652	7.45672	-8.70529
H	-2.62956	-5.81706	1.28952	H	-0.64245	8.10131	-9.5505
C	-2.71818	-4.51327	-4.74675	H	-2.92044	6.08873	-8.99437
C	-2.50473	-4.30269	-6.16123	H	-3.27112	7.44826	-7.85623
H	-1.53956	-4.59039	-6.58424	H	-4.56725	6.23066	-8.2419
C	-3.48007	-3.77887	-6.95576	H	3.3015	6.70975	5.88619
H	-3.32719	-3.63852	-8.02717	H	2.56853	5.38076	6.89251
C	-4.7474	-3.41141	-6.39253	H	2.98033	6.96714	7.64687
H	-5.513	-3.01043	-7.06065	H	-0.81841	9.73455	7.73119
C	-4.98024	-3.56305	-5.05677	H	-0.68949	9.63224	5.92704
H	-5.94088	-3.27768	-4.63425	H	0.65459	9.0151	6.97554
C	-3.97616	-4.12429	-4.18287	H	-3.30299	7.74113	7.40751
C	1.88553	4.90903	4.37833	H	-3.21224	8.84963	5.98281
C	-4.01466	4.53092	-4.18936	H	-4.74545	7.93434	6.31522
C	1.86518	4.82646	-4.56362	H	4.42479	-7.22009	7.4413
C	-7.81033	3.34173	2.39517	H	2.67099	-7.66357	7.61449
H	-7.91639	4.2915	2.93661	H	3.41403	-6.45913	8.7409

C	-7.88327	-4.47427	-1.95036	H	1.84297	-5.28752	9.52995
H	-8.74636	-5.13352	-2.10491	H	0.91175	-6.67105	10.2301
C	8.72594	-1.21291	1.33921	H	0.04431	-5.19646	9.64172
H	9.54891	-0.72399	0.79851	H	-1.21941	-7.50459	7.94658
C	-1.82171	-4.07056	4.86111	H	-2.94409	-6.93034	7.95981
C	5.29964	-4.15464	-3.24169	H	-1.72404	-6.17989	9.07114
C	4.17761	-4.95219	-3.63754	H	2.57827	-7.21277	-8.1164
C	4.13652	-6.33488	-3.22037	H	3.57601	-8.29447	-7.06535
H	3.27519	-6.93678	-3.51991	H	4.34329	-6.85958	-7.86688
C	5.14858	-6.88645	-2.49205	H	2.25789	-9.30191	-5.74022
H	5.13074	-7.93743	-2.19924	H	1.25767	-10.06167	-7.04173
C	6.27989	-6.09045	-2.11521	H	0.5053	-9.62261	-5.45614
H	7.0844	-6.56954	-1.55235	H	-1.41928	-9.22554	-6.21559
C	6.34572	-4.77172	-2.46172	H	-2.83128	-8.23813	-6.77944
H	7.20783	-4.17437	-2.17646	H	-1.19463	-8.01029	-7.53715
C	-1.80711	-0.43224	5.73579				

Table S10. Cartesian coordinates of **1•(F)**$_2$ (R = OCH_3; E_{opt} = 1.50940930253 and E_{sp} = –9929.46408226 hartree).

H	1.44649	-3.30976	-2.95774	C	-2.4171	-4.26116	-7.96434
C	1.21925	-3.73747	-0.84895	C	-3.48776	-4.15265	-7.02785
H	2.20509	-4.20096	-0.76004	C	-3.27432	-3.54113	-5.77552
C	0.37748	-3.65879	0.26344	C	-1.96551	-3.13935	-5.44166
H	0.70531	-4.06324	1.23059	H	-1.78167	-2.70945	-4.44815
C	-0.8944	-3.09551	0.14113	C	-3.3273	2.94106	4.83745
H	-1.55562	-3.06336	1.01391	C	3.25985	-6.58354	-1.62297
C	-1.33462	-2.61578	-1.10124	H	3.2538	-6.80381	-2.69234
C	-0.4822	-2.68215	-2.21225	C	-5.46652	-1.92053	3.09041
H	-0.81793	-2.32026	-3.19053	H	-4.67267	-1.5588	2.41175
C	0.79016	-3.24413	-2.08549	C	7.16453	1.36628	2.11287
C	-2.72887	-2.07663	-1.25696	C	4.22813	-5.80022	-1.06673
C	-2.84536	-0.58342	-0.89696	H	5.02134	-5.38336	-1.68336
C	-3.41264	-0.43526	0.52338	C	-4.63477	3.74984	-2.67383
N	-3.78551	0.15353	-1.77144	C	-3.53621	4.46562	-3.2502
O	-4.44039	0.11332	0.87708	C	-2.96746	3.98142	-4.48766
H	-3.44835	-2.6985	-0.67734	H	-2.15301	4.55709	-4.93762
H	-3.06712	-2.2378	-2.30988	C	-3.44053	2.85438	-5.08965
H	-1.82252	-0.10296	-0.90081	H	-3.0267	2.49822	-6.03293
H	-3.34261	0.96439	-2.20809	C	-4.5296	2.12887	-4.50217
H	-4.17532	-0.43035	-2.51069	H	-4.89385	1.23775	-5.01828
O	-2.70653	-0.98544	1.55799	C	-5.1049	2.55841	-3.34306
H	-1.90841	-1.55298	1.3128	H	-5.94317	2.01512	-2.90855
Pt	6.98371	-0.36976	-1.75797	C	-5.04582	-5.62567	2.74103
Pt	-6.95612	0.24567	1.78326	C	-6.19062	-2.62012	-2.7386
N	6.89851	1.50478	-2.4346	C	3.19856	-6.08251	1.16482
C	5.70061	2.1613	-2.51738	C	3.74696	4.75209	-1.94319
H	4.80047	1.60891	-2.18928	C	8.9747	0.15324	0.37374
C	5.62481	3.48271	-2.9952	H	9.65482	-0.33665	-0.33712
C	6.81308	4.12902	-3.38575	C	0.21945	0.83842	-5.98238
H	6.77452	5.16183	-3.75847	H	-0.48423	1.63192	-6.23043
C	8.02939	3.44785	-3.29723	C	1.82164	5.0787	-5.75802
H	8.96424	3.93632	-3.59953	H	0.87509	5.6197	-5.83547

C	8.05443	2.13219	-2.82058	C	-0.67149	3.56812	5.60508
H	8.98926	1.5593	-2.73668	C	6.14765	1.95279	3.00786
N	7.66904	0.30868	-0.00836	C	6.78653	-1.55932	-5.81492
N	7.15127	-2.25036	-1.10975	H	7.51004	-1.63261	-6.63638
C	8.30612	-2.93402	-1.39342	C	2.5819	5.97545	0.33733
H	9.06396	-2.40929	-1.99331	H	2.17637	6.4521	1.22846
C	8.49662	-4.24272	-0.93763	C	1.94568	6.05958	-0.86549
H	9.42404	-4.77661	-1.18053	H	1.00657	6.60928	-0.96509
C	7.50433	-4.85877	-0.17065	C	3.11658	3.61206	5.59085
H	7.6411	-5.88206	0.20554	C	1.78042	3.37286	5.25206
C	6.32002	-4.15868	0.12345	H	1.55914	2.7691	4.36786
C	6.16359	-2.85061	-0.37685	C	0.71861	3.88627	5.99636
H	5.23954	-2.27149	-0.20388	C	1.0044	4.70842	7.12582
C	-3.95307	-3.72816	3.94475	C	2.34308	4.88426	7.54748
N	-6.42311	1.53502	3.20376	C	3.4022	4.36346	6.767
C	-7.4126	2.19987	3.88079	C	-5.24073	-2.21199	-4.96805
H	-8.45169	1.96715	3.60546	C	-6.19342	-1.89139	-3.94658
C	-7.09779	3.13181	4.87528	C	-7.14373	-0.83269	-4.19528
H	-7.901	3.65833	5.40546	H	-7.87592	-0.59959	-3.4243
C	-5.75946	3.38215	5.18455	C	-7.15721	-0.1649	-5.38445
H	-5.49226	4.11127	5.96169	H	-7.89687	0.61245	-5.59166
C	-4.74217	2.69119	4.49704	C	-6.19616	-0.47598	-6.40492
C	-5.10671	1.76728	3.49958	H	-6.24048	0.07651	-7.3461
H	-4.35442	1.20035	2.91874	C	-5.26829	-1.45185	-6.19761
C	2.47383	0.24883	-5.29299	H	-4.53409	-1.69649	-6.97036
H	3.48309	0.53329	-5.00304	C	6.48274	-3.87489	4.37645
C	1.43164	-3.89423	-5.62933	H	7.33531	-3.41285	4.87883
C	2.77194	-3.52788	-5.28076	C	3.4339	0.7487	5.31999
C	3.73823	-4.57761	-5.05885	H	2.76596	1.1686	6.07706
H	4.75811	-4.30263	-4.79958	C	2.49913	5.44739	-2.05152
C	3.39387	-5.89128	-5.19133	C	8.50647	1.20716	2.50514
H	4.13093	-6.68645	-5.05844	H	8.8319	1.55243	3.49572
C	2.04868	-6.26123	-5.52352	C	3.65888	3.85348	-6.7591
H	1.80849	-7.32141	-5.62151	H	4.10642	3.45007	-7.67025
C	1.10548	-5.29908	-5.72892	C	2.19083	-6.90173	0.53029
H	0.08048	-5.57313	-5.99152	H	1.41514	-7.34106	1.16187
N	-6.6664	-1.26242	3.06139	C	3.47161	-0.59684	5.10525
C	2.15458	-1.15658	-5.40395	H	2.83083	-1.27947	5.66223
C	0.47479	-2.88947	-5.90262	C	-0.26796	-5.97598	4.0952
C	6.7833	0.92316	0.83402	C	0.82559	-5.63188	3.29894
H	5.75149	1.05434	0.45942	H	0.73124	-4.78874	2.59935
C	-3.89404	-5.01525	3.3634	C	2.04301	-6.31262	3.37095
C	0.82277	-1.52588	-5.78456	C	2.15427	-7.43914	4.23548
C	4.99817	-1.35381	-3.69603	C	1.04417	-7.84259	5.01375
H	4.33156	-1.25158	-2.8184	C	-0.14356	-7.07429	4.99484
C	5.28208	-0.33634	3.50826	C	-6.2728	-3.38128	4.84122
H	6.03352	-0.76777	2.8499	H	-6.11617	-4.2139	5.54043
C	4.26055	-5.15555	3.16807	C	-7.48862	-2.69345	4.81476
C	5.259	1.09709	3.69367	H	-8.30107	-2.97492	5.49639
C	5.31733	-4.62261	2.3607	C	4.39209	4.69042	-0.65153
C	-2.66367	-5.74873	3.3883	H	5.35574	4.192	-0.57376
C	0.57262	6.276	-3.43094	C	3.83231	5.28093	0.44325
C	-0.61601	5.7004	-3.00227	H	4.32948	5.25331	1.41631

H	-0.58454	4.72262	-2.51029	C	-4.97455	-6.87521	2.1977
C	-1.86608	6.3275	-3.18334	C	-5.31571	-3.71319	-2.55844
C	-1.90627	7.56192	-3.86305	C	-5.32304	-4.50827	-1.35274
C	-0.69589	8.20144	-4.26302	H	-6.03938	-4.26394	-0.57103
C	0.54186	7.57463	-4.02831	C	-4.47479	-5.56623	-1.20301
C	4.18701	3.05908	4.73541	H	-4.48224	-6.17478	-0.29486
N	6.31712	-1.02818	-3.51378	C	-3.55179	-5.91395	-2.24509
C	-1.52685	-5.20045	4.02506	H	-2.90295	-6.77863	-2.09853
C	3.68811	4.27474	-4.35012	C	-3.50955	-5.18271	-3.39391
C	3.11836	-2.16114	-5.17869	H	-2.82216	-5.44975	-4.20209
C	5.41832	-4.39406	5.18676	C	-4.37915	-4.04486	-3.59027
H	5.49983	-4.30432	6.27127	C	-3.73899	-7.60349	2.20566
C	-2.80989	-3.16028	4.54686	H	-3.71928	-8.59983	1.7616
C	-2.8307	-1.84118	5.13689	C	4.24501	-5.53505	0.35354
H	-3.75527	-1.26911	5.10308	C	5.26471	-4.77037	0.95717
C	-1.72486	-1.32125	5.74144	C	-2.53115	3.74881	3.99852
H	-1.74138	-0.32537	6.19179	C	-3.02533	4.27126	2.745
C	-0.51086	-2.08218	5.81802	H	-4.04181	4.02933	2.44236
H	0.33976	-1.64917	6.34181	C	-2.24872	5.07159	1.96103
C	-0.45053	-3.32906	5.27232	H	-2.62313	5.47321	1.01644
H	0.45883	-3.92959	5.35158	C	-0.92314	5.43239	2.37353
C	-1.58902	-3.90956	4.59668	H	-0.34608	6.10261	1.73726
N	-7.57219	-1.02832	0.37761	C	-0.42467	4.96526	3.55235
C	-6.74549	-1.33931	-0.66663	H	0.56692	5.2694	3.89602
H	-5.76794	-0.81931	-0.69547	C	-1.19597	4.08155	4.39696
C	-7.13129	-2.26279	-1.65607	H	-5.98549	-5.0783	2.72982
C	-8.40907	-2.8443	-1.57473	H	-5.85206	-7.35165	1.75487
H	-8.7353	-3.56069	-2.34003	O	-1.24441	-7.2502	5.76361
C	-9.25399	-2.50271	-0.51464	O	1.1507	-8.9368	5.86173
H	-10.25684	-2.94215	-0.43957	O	3.38543	-8.00053	4.20358
C	-8.81709	-1.59693	0.45841	O	-2.99553	8.30755	-4.19347
H	-9.44678	-1.30973	1.31271	O	-0.70281	9.35721	-5.01821
C	-5.23975	-3.00181	3.96294	O	1.77357	8.04305	-4.34506
C	1.8582	5.55198	-3.30611	O	-4.64531	-4.66157	-7.52831
C	4.53108	-1.7936	-4.94825	O	-2.55961	-4.94563	-9.15417
C	4.32141	4.16877	-3.09196	O	-0.01695	-3.83097	-8.394
C	-2.62838	-7.05811	2.7771	O	-0.10668	5.24231	7.68308
H	-1.68427	-7.60742	2.79565	O	2.62147	5.63971	8.67857
C	4.34589	-4.99917	4.60251	O	4.72992	4.49611	6.99345
H	3.53684	-5.40714	5.21308	C	-4.32397	7.72469	-4.19649
C	6.42392	-3.96536	3.01628	C	-1.33314	10.5136	-4.39587
H	7.2352	-3.57696	2.4052	C	1.90531	9.35186	-4.97698
N	-7.25823	1.77168	0.5299	C	-5.76426	-4.96338	-6.65459
C	-8.53515	2.22871	0.32289	C	-3.57514	-4.43272	-10.06392
H	-9.34768	1.69728	0.8392	C	-0.09655	-4.35318	-9.75458
C	-8.77584	3.32333	-0.51259	C	-1.21382	-8.17616	6.89072
H	-9.80383	3.66879	-0.67859	C	0.72439	-10.18925	5.2448
C	-7.70132	3.97666	-1.1237	C	3.74552	-9.05055	5.14953
H	-7.87415	4.84705	-1.76988	C	0.00686	6.28609	8.69691
C	-6.39056	3.51722	-0.90194	C	2.64383	4.8535	9.90837
C	-6.20007	2.3877	-0.08208	C	5.2207	5.3786	8.04586
H	-5.19504	1.94818	0.08562	H	-4.87025	8.4533	-4.81766
C	4.2862	1.65363	4.58338	H	-4.71788	7.71011	-3.17683

C	-5.23577	4.22817	-1.49122	H	-4.32752	6.73941	-4.66789
C	-4.77478	5.41653	-0.88096	H	-0.85433	10.74731	-3.44432
C	-5.39737	5.95792	0.30463	H	-2.40936	10.33859	-4.28132
H	-6.24342	5.42912	0.73825	H	-1.14519	11.29236	-5.14751
C	-4.95816	7.1227	0.86302	H	1.57918	10.13203	-4.28437
H	-5.44111	7.54658	1.74678	H	1.32862	9.38381	-5.90994
C	-3.84313	7.82696	0.2966	H	2.98664	9.39015	-5.16079
H	-3.52051	8.75682	0.76973	H	-6.32014	-4.04636	-6.44256
C	-3.21754	7.3387	-0.81082	H	-5.43651	-5.48096	-5.75016
H	-2.36699	7.86794	-1.24934	H	-6.353	-5.63983	-7.2959
C	-3.66403	6.12072	-1.44998	H	-3.43341	-5.0868	-10.93529
C	1.54408	1.20648	-5.56911	H	-3.38111	-3.38773	-10.30795
H	1.7878	2.27026	-5.49637	H	-4.57316	-4.57528	-9.63276
C	4.40871	-1.14735	4.17122	H	-0.46929	-5.38524	-9.74457
H	4.42083	-2.2313	4.02665	H	-0.73616	-3.70818	-10.3626
C	5.06528	3.91115	4.02795	H	0.95516	-4.29924	-10.06349
C	4.98674	5.3509	4.13933	H	-2.04098	-7.79547	7.50469
H	4.21178	5.77674	4.78118	H	-0.25685	-8.13127	7.42298
C	5.86159	6.16331	3.48238	H	-1.41707	-9.18484	6.5202
H	5.81202	7.24978	3.57989	H	-0.33181	-10.1387	4.96953
C	6.88675	5.5983	2.65278	H	0.89075	-10.90753	6.05906
H	7.5838	6.27813	2.15628	H	1.34945	-10.42047	4.37835
C	6.98264	4.24615	2.49775	H	3.36519	-8.83466	6.15428
H	7.76391	3.82104	1.87104	H	4.84266	-9.00887	5.10832
C	6.07582	3.35219	3.17767	H	3.36606	-10.0054	4.77423
C	-4.32815	-3.27437	-4.77479	H	0.82378	6.98102	8.47125
C	2.43952	4.96825	-4.45544	H	0.14435	5.81111	9.67234
C	-3.04854	5.63793	-2.62756	H	-0.97543	6.77122	8.61711
C	5.44585	-1.89009	-6.01689	H	1.66468	4.40518	10.09294
H	5.09596	-2.22734	-7.00247	H	2.87932	5.62015	10.65919
C	9.4111	0.60627	1.62582	H	3.4265	4.09186	9.86059
H	10.46335	0.48129	1.91181	H	5.16802	4.84812	9.0011
C	-7.67215	-1.63867	3.91302	H	6.2609	5.533	7.72749
H	-8.61449	-1.07537	3.85866	H	4.65823	6.31869	8.07714
C	3.19041	-5.85001	2.55852	H	-1.951	3.27711	-0.18799
C	-2.8074	2.42178	6.04427	C	-1.11324	2.20552	-1.86937
C	-1.45815	2.72204	6.42016	H	-2.00691	2.3181	-2.4874
C	-0.94543	2.15692	7.64828	C	0.01184	1.54765	-2.37603
H	0.08707	2.37915	7.92537	H	-0.00758	1.1239	-3.38304
C	-1.72466	1.37962	8.45162	C	1.17407	1.44891	-1.60719
H	-1.344	0.9657	9.38693	H	2.05095	0.95598	-2.0247
C	-3.08259	1.09829	8.0836	C	1.21545	2.00823	-0.32083
H	-3.68451	0.48483	8.7581	C	0.07908	2.65199	0.19267
C	-3.59895	1.58922	6.92019	H	0.09816	3.1053	1.18544
H	-4.62729	1.36748	6.64352	C	-1.07888	2.75299	-0.58349
C	2.22091	-7.14797	-0.8095	C	2.48014	1.96115	0.49226
H	1.47525	-7.78374	-1.28726	C	2.86791	0.56427	1.00349
C	7.20635	-1.12967	-4.55142	C	3.47183	-0.28473	-0.12354
H	8.25181	-0.8573	-4.3446	N	1.75753	-0.23123	1.58473
C	4.26815	3.71083	-5.54651	O	4.45484	0.03736	-0.78338
H	5.21755	3.18622	-5.47026	H	2.40595	2.65305	1.36234
C	-0.12339	-0.47582	-6.08808	H	3.31671	2.38974	-0.11197
H	-1.11884	-0.76762	-6.43064	H	3.69652	0.6902	1.76766

C	2.40824	4.54798	-6.86776	H	0.85335	0.21195	1.42444
H	1.95454	4.65074	-7.85511	H	1.87419	-0.33842	2.59386
C	-0.89474	-3.27338	-6.31519	O	2.97015	-1.50682	-0.38936
C	-1.13066	-3.80264	-7.62244	H	2.14471	-1.74559	0.18183

Table S11. Cartesian coordinates of **1•FF** (R = OCH_3; E_{opt} = 1.59981062249 and E_{sp} = –9853.09845752 hartree).

Pt	7.22676	-0.78495	-0.56344	H	-4.53875	4.11954	-1.83956
Pt	-7.20138	0.84482	0.57688	C	-6.5775	-4.65145	-2.108
N	7.56915	1.05945	0.12423	C	-5.18455	0.82012	-4.44193
C	6.53861	1.95966	0.21135	C	1.71298	-5.33673	-2.39084
H	5.54148	1.62238	-0.10913	C	4.82647	4.28001	1.9209
C	6.7477	3.26805	0.68651	C	8.48344	-2.16279	1.69796
C	8.04898	3.64184	1.07928	H	9.30775	-2.24205	0.97444
H	8.23203	4.65986	1.45306	C	2.10719	4.09804	-4.4884
C	9.08975	2.717	0.98572	H	1.54419	5.02242	-4.36606
H	10.10965	2.99307	1.28487	C	4.08887	6.92163	-1.37221
C	8.83239	1.42667	0.50362	H	3.25134	7.62297	-1.32085
H	9.62699	0.67195	0.41513	C	-1.87452	-0.38884	6.40043
N	7.34863	-1.51205	1.29172	C	5.17586	-1.7657	4.35612
N	6.86833	-2.62448	-1.25632	C	8.21257	0.81801	-4.37677
C	7.88788	-3.33508	-1.8337	H	9.13992	1.12464	-4.87837
H	8.87389	-2.85137	-1.88699	C	3.2264	4.46273	4.25822
C	7.66764	-4.62302	-2.33627	H	2.65456	4.52729	5.18326
H	8.49494	-5.17674	-2.79917	C	2.96759	5.28801	3.20439
C	6.39624	-5.19234	-2.24522	H	2.17477	6.03848	3.26242
H	6.20454	-6.2023	-2.63502	C	1.76424	-1.30035	6.9513
C	5.34704	-4.46528	-1.64765	C	0.54127	-0.99551	6.34357
C	5.6154	-3.17114	-1.16245	H	0.48765	-0.9462	5.25154
H	4.82193	-2.56067	-0.69594	C	-0.60815	-0.72666	7.08648
C	-5.62186	-4.29283	0.16972	C	-0.53409	-0.76712	8.51054
N	-6.99939	0.93156	2.55672	C	0.65965	-1.1856	9.14337
C	-8.04213	1.34138	3.34476	C	1.82022	-1.41808	8.36901
H	-8.98364	1.60029	2.83936	C	-3.67751	2.07395	-5.92521
C	-7.89644	1.42494	4.73465	C	-4.83266	2.03007	-5.0791
H	-8.74023	1.75781	5.35187	C	-5.60726	3.23736	-4.90742
C	-6.6773	1.08486	5.32523	H	-6.49319	3.20368	-4.2769
H	-6.54623	1.14759	6.41392	C	-5.25565	4.39588	-5.53584
C	-5.60705	0.65663	4.51603	H	-5.85014	5.30569	-5.42457
C	-5.80076	0.59193	3.12424	C	-4.08826	4.44629	-6.36933
H	-4.99432	0.27326	2.43797	H	-3.83694	5.39118	-6.8551
C	4.09635	2.82339	-3.91752	C	-3.32925	3.3296	-6.55161
H	5.03715	2.72384	-3.382	H	-2.44064	3.35833	-7.18765
C	2.82688	-0.25926	-6.60963	C	4.51497	-7.37421	1.35641
C	4.08361	-0.38335	-5.93311	H	5.29221	-7.7061	2.04867
C	4.90033	-1.54398	-6.19665	C	2.0397	-3.63677	5.25804
H	5.85966	-1.63356	-5.69383	H	1.25124	-3.57514	6.01374
C	4.49793	-2.50029	-7.08383	C	3.74309	5.21485	1.98782
H	5.12307	-3.36841	-7.305	C	7.49928	-2.58445	3.86725
C	3.23682	-2.38419	-7.75657	H	7.55286	-3.00652	4.88104
H	2.94911	-3.16558	-8.46264	C	6.03241	6.01168	-2.4995
C	2.43315	-1.30806	-7.52359	H	6.69296	6.05004	-3.36878

H	1.47602	-1.20841	-8.04271	C	0.72373	-4.99384	-3.38754
N	-7.55479	-1.11429	0.69897	H	-0.23107	-5.525	-3.36951
C	3.69306	1.76874	-4.81825	C	2.09466	-4.68914	4.39286
C	2.02303	0.88551	-6.40049	H	1.36053	-5.49369	4.4286
C	6.29004	-1.38035	2.1504	C	-2.26607	-6.8039	-0.84453
H	5.40064	-0.83566	1.77759	C	-1.01123	-6.20939	-0.9812
C	-5.55221	-4.87384	-1.11474	H	-0.9126	-5.12934	-0.80702
C	2.45184	1.90487	-5.51941	C	0.12618	-6.95057	-1.32012
C	5.90755	0.01533	-3.0611	C	-0.01612	-8.33917	-1.60543
H	5.01568	-0.32671	-2.51019	C	-1.28295	-8.95889	-1.50068
C	4.15004	-3.83918	3.41377	C	-2.39405	-8.20882	-1.0512
H	4.97914	-3.9346	2.71577	C	-7.98522	-3.86648	0.94276
C	2.45435	-6.62877	-0.44424	H	-8.15202	-4.9472	1.04239
C	4.13705	-2.7196	4.32729	C	-9.00284	-2.95667	1.24983
C	3.74827	-6.02125	-0.53193	H	-9.98264	-3.31541	1.58885
C	-4.43825	-5.70904	-1.45369	C	5.06017	3.41482	3.05281
C	2.27349	6.93292	0.91286	H	5.88019	2.70292	3.00657
C	0.99548	6.39448	0.79812	C	4.28928	3.50438	4.17539
H	0.87995	5.30591	0.73445	H	4.4695	2.85739	5.03717
C	-0.16036	7.19761	0.7558	C	-6.49882	-5.22688	-3.34195
C	-0.00456	8.60004	0.7891	C	-4.41008	-0.34485	-4.62974
C	1.28883	9.1755	0.96218	C	-4.72427	-1.58846	-3.9652
C	2.42271	8.34673	1.04748	H	-5.57181	-1.61817	-3.28283
C	2.96571	-1.48294	6.1106	C	-3.98301	-2.71122	-4.18539
N	7.11838	-0.07584	-2.42641	H	-4.21924	-3.65237	-3.68111
C	-3.44244	-5.98027	-0.48817	C	-2.87744	-2.68673	-5.10021
C	5.3991	5.11156	-0.3166	H	-2.33052	-3.61234	-5.27673
C	4.49389	0.62734	-5.03583	C	-2.54427	-1.53088	-5.7396
C	3.21291	-7.96525	1.46392	H	-1.71606	-1.50457	-6.4524
H	3.04508	-8.7183	2.23586	C	-3.28149	-0.30839	-5.51149
C	-4.63759	-4.57133	1.14128	C	-5.37475	-6.04832	-3.69107
C	-4.68989	-4.00421	2.47005	H	-5.34621	-6.49156	-4.68763
H	-5.50603	-3.32778	2.71499	C	2.99336	-4.69605	-2.4544
C	-3.75495	-4.32317	3.4095	C	3.99903	-5.05488	-1.53147
H	-3.79632	-3.90002	4.41627	C	-3.21262	1.162	5.02952
C	-2.69835	-5.24441	3.10127	C	-3.28434	2.40634	4.29772
H	-2.00415	-5.51877	3.89379	H	-4.21855	2.67013	3.80692
C	-2.60563	-5.78626	1.85399	C	-2.2185	3.25535	4.24283
H	-1.82803	-6.51627	1.61675	H	-2.27282	4.19993	3.69467
C	-3.55212	-5.44686	0.81625	C	-1.00015	2.93825	4.92969
N	-7.4125	0.76979	-1.40393	H	-0.17999	3.65421	4.88896
C	-6.29861	0.8039	-2.19917	C	-0.89796	1.77945	5.63957
H	-5.32156	0.85175	-1.68698	H	0.01161	1.55143	6.20035
C	-6.39878	0.76662	-3.602	C	-1.98738	0.8312	5.69419
C	-7.67852	0.6812	-4.18216	H	-7.43264	-4.03284	-1.84434
H	-7.7821	0.64466	-5.27424	H	-7.28419	-5.08295	-4.08707
C	-8.80798	0.64334	-3.36059	O	-3.63639	-8.66881	-0.77587
H	-9.81203	0.57584	-3.79792	O	-1.41606	-10.31736	-1.75511
C	-8.65783	0.69195	-1.96973	O	1.15559	-8.92452	-1.95217
H	-9.51998	0.66687	-1.28757	O	-0.96235	9.56171	0.703
C	-6.74026	-3.38628	0.50208	O	1.48708	10.5404	0.89785
C	3.46261	6.05247	0.88406	O	3.71248	8.73234	1.20859
C	5.80624	0.52036	-4.37044	O	-2.85935	1.06437	-9.24188

C	5.62684	4.22437	0.75976	O	-0.55782	1.26528	-10.5481
C	-4.38286	-6.2727	-2.78405	O	1.90314	1.4044	-9.05755
H	-3.528	-6.90157	-3.04047	O	-1.68321	-0.37752	9.10867
C	2.22312	-7.60039	0.60051	O	0.71812	-1.27858	10.52738
H	1.2316	-8.05464	0.67119	O	3.05508	-1.74643	8.81723
C	4.76714	-6.42546	0.40839	C	-2.29858	9.24931	0.23405
H	5.75514	-5.97807	0.33353	C	0.79886	11.32513	1.91422
N	-6.88657	2.81357	0.47285	C	4.02675	10.14842	1.36825
C	-7.95529	3.65571	0.30974	C	-4.14722	0.59532	-8.76541
H	-8.95206	3.19522	0.24428	C	-1.33126	2.33355	-11.16682
C	-7.76934	5.04048	0.23042	C	2.01954	1.65431	-10.49051
H	-8.63595	5.70054	0.09986	C	-3.89405	-10.1037	-0.71389
C	-6.48027	5.57129	0.31801	C	-1.80299	-10.61206	-3.13144
H	-6.31881	6.65501	0.25573	C	1.24642	-10.37301	-2.09317
C	-5.38108	4.70777	0.48845	C	-1.7207	-0.14006	10.54818
C	-5.61954	3.32336	0.56554	C	0.33942	-2.59379	11.03501
H	-4.79414	2.59692	0.70056	C	3.35233	-1.73779	10.2451
C	3.03656	-2.59202	5.23357	H	-2.67359	10.25651	-0.01056
C	-4.01089	5.25297	0.58102	H	-2.87698	8.80573	1.04893
C	-3.57641	5.84015	1.78955	H	-2.27132	8.62189	-0.6597
C	-4.40334	5.84944	2.97358	H	1.08653	10.99754	2.91392
H	-5.39131	5.39683	2.92383	H	-0.28524	11.26922	1.76117
C	-3.96914	6.42566	4.13161	H	1.16999	12.33547	1.69506
H	-4.59736	6.44765	5.02537	H	3.59583	10.52051	2.30134
C	-2.66644	7.02353	4.20352	H	3.6591	10.71941	0.50645
H	-2.35156	7.47116	5.14824	H	5.12339	10.11878	1.40509
C	-1.85755	7.03514	3.1072	H	-4.63878	1.39257	-8.20129
H	-0.86502	7.49149	3.15086	H	-4.04919	-0.33085	-8.19444
C	-2.28385	6.45511	1.85343	H	-4.66748	0.41026	-9.71922
C	3.33316	3.94299	-3.76067	H	-1.10383	2.17659	-12.22999
H	3.64403	4.74918	-3.09073	H	-0.9872	3.30861	-10.82006
C	3.1658	-4.78304	3.44408	H	-2.40015	2.18374	-10.9739
H	3.18243	-5.63988	2.76495	H	1.55356	0.83879	-11.0578
C	4.01795	-0.53899	6.14554	H	1.56446	2.61707	-10.73721
C	4.00703	0.58049	7.06016	H	3.1099	1.67103	-10.61439
H	3.13651	0.70792	7.70865	H	-4.79867	-10.13272	-0.09102
C	5.05885	1.44407	7.13746	H	-3.06161	-10.64367	-0.24896
H	5.0632	2.272	7.84907	H	-4.0904	-10.46687	-1.72657
C	6.20111	1.26546	6.28887	H	-2.78128	-10.17885	-3.3537
H	7.03633	1.96233	6.39294	H	-1.84624	-11.7096	-3.12143
C	6.2368	0.24618	5.38185	H	-1.0419	-10.25082	-3.82778
H	7.10597	0.1135	4.74233	H	0.69121	-10.8897	-1.30201
C	5.14876	-0.69687	5.27963	H	2.32963	-10.53024	-2.00357
C	-2.92286	0.89949	-6.1516	H	0.88028	-10.6522	-3.08543
C	4.29645	6.02417	-0.25889	H	-0.80209	0.34191	10.90156
C	-1.46904	6.51151	0.69887	H	-1.89186	-1.09389	11.05499
C	6.98418	0.92401	-5.03101	H	-2.59011	0.52591	10.63346
H	6.92487	1.31772	-6.05602	H	-0.69661	-2.82069	10.77233
C	8.57601	-2.70397	2.9867	H	0.45495	-2.4566	12.11887
H	9.49476	-3.21982	3.29619	H	1.02151	-3.35988	10.65743
C	-8.77121	-1.58259	1.12718	H	3.00236	-2.67774	10.68181
H	-9.54184	-0.83653	1.3661	H	4.44938	-1.68145	10.23552
C	1.4524	-6.29981	-1.38879	H	2.9021	-0.87203	10.744

C	-4.19849	-0.94803	5.80359	H	4.24685	-0.13263	0.83463
C	-2.96037	-1.29335	6.43592	C	3.04277	-0.02465	-0.96144
C	-2.86681	-2.56709	7.11371	H	3.50784	0.8887	-1.33073
H	-1.91831	-2.82919	7.58656	C	2.00854	-0.61803	-1.69622
C	-3.92996	-3.41703	7.17703	H	1.71264	-0.19333	-2.65711
H	-3.86604	-4.37166	7.70175	C	1.35032	-1.74235	-1.19741
C	-5.1751	-3.05978	6.55997	H	0.54125	-2.20392	-1.77171
H	-6.01255	-3.75626	6.64489	C	1.71746	-2.28174	0.0478
C	-5.30059	-1.87799	5.89037	C	2.78202	-1.71323	0.7609
H	-6.24593	-1.61415	5.42122	H	3.09262	-2.1415	1.71477
C	0.97333	-4.05153	-4.33965	C	3.43675	-0.5847	0.25561
H	0.2304	-3.79554	-5.09405	C	0.9427	-3.43548	0.62054
C	8.26228	0.31645	-3.06997	C	-0.23669	-2.97222	1.50533
H	9.20988	0.22139	-2.52052	C	-1.12514	-2.03673	0.6661
C	6.25107	5.12874	-1.48116	N	0.15707	-2.30165	2.76612
H	7.09692	4.44754	-1.52075	O	-1.59103	-2.43786	-0.41498
C	1.68647	3.11645	-5.33561	N	-1.42455	-0.76748	1.1234
H	0.76701	3.2351	-5.91462	C	-2.02263	0.25054	0.23428
C	4.92664	6.92252	-2.44807	C	-1.86627	1.66207	0.82374
H	4.78694	7.62033	-3.27592	C	0.49671	2.13653	1.56804
C	0.72246	1.01034	-7.09503	H	0.24542	1.7801	2.56622
C	0.67595	1.24311	-8.50383	C	1.7964	2.57064	1.2923
C	-0.56505	1.24276	-9.16786	H	2.55363	2.54987	2.0783
C	-1.7707	1.06546	-8.42716	C	2.12565	3.04383	0.01836
C	-1.72874	0.94824	-7.0216	H	3.13913	3.38495	-0.19256
C	-0.47119	0.89484	-6.39054	C	1.14753	3.10329	-0.97928
H	-0.43624	0.75517	-5.30673	H	1.39856	3.48637	-1.9698
C	-4.3107	0.27955	5.11354	C	-0.15402	2.67572	-0.71007
C	2.24573	-3.39132	-4.38219	H	-0.91597	2.74603	-1.48594
H	2.41681	-2.64429	-5.16175	C	-0.47708	2.17711	0.56134
C	-6.55991	-1.9952	0.38065	C	-3.5046	-0.09018	-0.00203
H	-5.6017	-1.57232	0.0157	O	-4.45924	0.58429	0.36955
C	6.33214	-1.91344	3.45133	O	-3.79013	-1.17778	-0.72456
C	3.22004	-3.70626	-3.48155	H	1.60441	-4.11116	1.20415
H	4.19157	-3.2215	-3.53737	H	0.54207	-4.07664	-0.20409
C	-3.17237	5.24252	-0.55463	H	-0.88504	-3.86862	1.74771
C	-1.89722	5.8934	-0.49885	H	1.07622	-1.86672	2.69195
C	-1.08512	5.92222	-1.69396	H	0.19863	-2.97009	3.53493
H	-0.1307	6.45419	-1.652	H	-1.09298	-0.44793	2.04378
C	-1.49523	5.31488	-2.8431	H	-1.5119	0.20893	-0.77815
H	-0.88884	5.34247	-3.74766	H	-2.618	2.34917	0.37018
C	-2.76057	4.64094	-2.88927	H	-2.10747	1.67685	1.91074
H	-3.0627	4.16747	-3.82767	H	-2.95188	-1.82191	-0.94881
C	-3.569	4.60998	-1.79121				

Table S12. Cartesian coordinates of **1•FFF** (R = OCH_3; E_{opt} = 1.57610808135 and E_{sp} = –10370.5072651 hartree).

H	-1.59368	-2.95598	3.58896	C	-0.26052	7.23596	-1.38105
C	-1.49911	-0.88893	2.96871	C	2.44594	-6.39176	2.42534
H	-2.5331	-0.67217	3.25817	C	6.91812	1.84905	-2.88137
C	-0.69597	0.11842	2.42312	C	3.64442	-2.08471	-5.68263
H	-1.1104	1.11265	2.25982	C	6.92654	2.06796	4.5625

C	0.63503	-0.14673	2.09518	H	7.70446	1.39436	4.9507
H	1.25863	0.63036	1.64324	C	1.53593	8.78014	2.62374
C	1.18106	-1.41832	2.32871	H	1.64434	9.75268	3.12107
C	0.36284	-2.43475	2.84409	C	-6.18208	-5.6866	-2.05324
H	0.76779	-3.42981	3.02634	H	-6.14592	-6.25912	-2.99047
C	-0.97158	-2.16802	3.16341	C	-2.94216	1.509	6.0577
C	2.64314	-1.66698	2.09292	C	-6.00466	0.69213	-4.17356
C	3.01282	-2.16071	0.67836	C	-5.89425	2.11201	-4.00196
C	2.83537	-1.00106	-0.32369	C	-7.03416	2.83271	-3.48012
N	2.261	-3.35365	0.21765	H	-6.94902	3.91689	-3.37304
O	2.96485	0.16196	0.02737	C	-8.18793	2.18881	-3.14716
N	2.62177	-1.36027	-1.66854	H	-9.05469	2.73319	-2.76816
C	1.77256	-0.56609	-2.58455	C	-8.29125	0.76691	-3.30877
C	2.13098	0.93231	-2.59686	H	-9.23472	0.28387	-3.04291
C	0.73391	1.91185	-0.72821	C	-7.23918	0.0464	-3.79408
H	1.20244	1.20922	-0.02667	H	-7.32525	-1.03088	-3.91903
C	-0.22014	2.81989	-0.26652	C	-0.33947	-1.1432	6.48506
H	-0.51524	2.8156	0.78178	H	-0.24602	-2.21045	6.68022
C	-0.80926	3.72746	-1.15077	C	6.09913	4.2859	4.09156
H	-1.55706	4.43296	-0.78576	H	6.19982	5.3811	4.09441
C	-0.42964	3.733	-2.49624	C	7.78525	2.83003	-0.78326
H	-0.8695	4.45721	-3.18478	H	7.71002	3.58178	-0.00226
C	0.52232	2.82414	-2.96278	C	6.34294	-2.8929	1.84894
H	0.82609	2.85165	-4.00969	H	6.3155	-3.9832	1.92037
C	1.10291	1.8975	-2.08104	C	8.92313	0.94246	-1.81006
C	0.31039	-0.93273	-2.25378	H	9.7636	0.2469	-1.77719
O	0.03068	-1.97631	-1.62766	C	5.03638	-4.22229	4.07264
H	3.01956	-2.40914	2.83772	C	5.87945	-5.15818	4.74252
H	3.2257	-0.73988	2.30551	C	5.55113	-6.52841	4.71298
H	4.11292	-2.42564	0.67301	C	4.40651	-6.9817	3.98986
H	1.2562	-3.17067	0.20558	C	3.60989	-6.06267	3.27715
H	2.4217	-4.14705	0.83575	C	3.93837	-4.69733	3.36634
H	2.57413	-2.38103	-1.86929	H	3.2909	-3.96758	2.86139
H	1.96128	-0.97631	-3.62758	C	-4.91517	-0.03684	-4.70002
H	3.06858	1.08544	-1.99442	C	0.8577	-0.37163	6.31673
H	2.43384	1.21429	-3.63073	H	1.81901	-0.88879	6.368
N	-0.76981	-0.20609	-2.71146	C	-5.05981	-4.19718	-0.58555
C	-2.10416	-0.52902	-2.13626	H	-4.14719	-3.60757	-0.38636
C	-1.99513	-1.83106	-1.31003	C	0.43579	7.33426	1.01777
O	-1.76332	-1.59222	0.00107	C	0.79086	0.97503	6.10855
O	-2.36035	-2.94416	-1.69747	H	1.70205	1.5576	6.00281
C	-2.62386	0.64208	-1.28862	C	2.35811	-3.75943	-4.41054
C	-4.09978	0.47142	-0.91188	C	3.56137	-3.00964	-4.61853
C	-5.37507	2.55221	-0.2569	C	4.67967	-3.23046	-3.72896
H	-5.75154	2.62824	-1.27593	H	5.60929	-2.6896	-3.92866
C	-5.77241	3.48885	0.70199	C	4.58262	-4.08784	-2.67526
H	-6.46113	4.28634	0.42467	H	5.4222	-4.25862	-2.00194
C	-5.29221	3.40442	2.01229	C	3.35832	-4.79761	-2.43658
H	-5.59881	4.13722	2.75698	H	3.30093	-5.44836	-1.55984
C	-4.41605	2.37308	2.36644	C	2.29434	-4.65168	-3.27818
H	-4.04174	2.30696	3.38862	H	1.37694	-5.20796	-3.10043
C	-4.02865	1.42593	1.4168	C	-5.37159	-5.39263	2.82697
H	-3.366	0.60944	1.69687	C	0.21011	-6.76635	0.71203

C	-4.50732	1.51157	0.09985	C	-1.68009	0.87925	6.15904
H	-0.69138	0.68573	-3.21087	C	4.8433	3.79249	-2.96609
H	-2.80912	-0.73493	-2.99819	C	2.38284	7.72222	2.96759
H	-1.60571	-2.40886	0.55456	H	3.16174	7.83077	3.73605
H	-2.49032	1.59965	-1.83663	C	6.85085	-2.27633	0.74477
H	-2.00196	0.74247	-0.36883	H	7.24048	-2.84861	-0.09695
H	-4.28616	-0.53578	-0.48183	C	8.00906	0.90297	-2.82037
H	-4.73938	0.53477	-1.81763	H	8.09703	0.16663	-3.62403
Pt	3.57757	5.09332	2.94293	C	-4.70168	2.77926	-4.35984
Pt	-3.507	-4.98341	-2.95671	C	-0.59854	7.12134	-0.01476
N	4.68625	5.43615	1.31716	C	7.10764	3.45206	4.5843
C	4.79295	4.52277	0.30371	H	8.03103	3.88796	4.98708
H	4.21082	3.58963	0.38605	C	3.10596	3.86095	-5.20864
C	5.62417	4.76792	-0.80805	H	2.45165	3.94658	-6.07511
C	6.33925	5.97851	-0.86913	C	4.0853	2.91511	-5.14933
H	6.99063	6.18344	-1.73089	H	4.23677	2.21579	-5.97553
C	6.21601	6.90484	0.16922	C	-3.5309	6.12835	-3.01022
H	6.76689	7.85369	0.13804	C	-3.76489	4.7574	-3.15048
C	5.38779	6.61454	1.25773	H	-3.28866	4.06156	-2.45228
H	5.26891	7.31351	2.09749	C	-4.56754	4.24249	-4.17309
N	2.27119	6.49743	2.35734	C	-5.21605	5.14949	-5.05946
N	2.52405	4.80243	4.6148	C	-5.088	6.54507	-4.86455
C	2.97512	5.40336	5.76198	C	-4.20818	7.03706	-3.87252
H	3.89563	5.99993	5.68911	C	2.634	-6.89736	1.11953
C	2.28717	5.25329	6.97122	C	1.50449	-7.10777	0.26149
H	2.66449	5.74102	7.87925	C	1.72928	-7.69601	-1.03884
C	1.12406	4.4827	7.01014	H	0.87359	-7.86571	-1.68895
H	0.56723	4.35247	7.94925	C	2.98081	-8.06044	-1.44081
C	0.65719	3.86445	5.83311	H	3.15076	-8.5336	-2.41083
C	1.38552	4.04215	4.64032	C	4.11556	-7.82735	-0.5922
H	1.06046	3.57635	3.69825	H	5.10274	-8.12564	-0.95113
C	-6.11341	-3.39099	1.53007	C	3.94763	-7.25582	0.6328
N	-4.45304	-3.74717	-4.21087	H	4.80492	-7.07131	1.28596
C	-5.1693	-4.25946	-5.26083	C	-3.17215	5.72562	5.59732
H	-5.1934	-5.35297	-5.36812	H	-3.26543	6.81092	5.52429
C	-5.83584	-3.41177	-6.15464	C	-4.30478	6.48246	-0.21967
H	-6.40598	-3.84106	-6.98837	H	-5.05733	6.33712	-0.99811
C	-5.76921	-2.02654	-5.98398	C	4.97627	2.82914	-4.01518
H	-6.28224	-1.35082	-6.68177	C	0.55843	8.58887	1.64482
C	-5.03357	-1.49535	-4.90796	H	-0.11586	9.40873	1.35755
C	-4.3928	-2.39336	-4.03302	C	8.80958	1.92811	-0.77416
H	-3.80419	-2.0325	-3.16887	H	9.56827	1.93945	0.01139
C	6.48085	-0.08602	1.73249	C	-1.55873	-0.53865	6.41128
H	6.53807	0.99913	1.68339	H	-2.47527	-1.11444	6.55814
C	4.91157	-1.9983	5.23074	C	-4.65839	6.54679	1.09505
C	5.04713	-0.57147	5.2045	H	-5.69985	6.47117	1.40792
C	4.67274	0.17757	6.38078	C	-5.63584	-0.93854	5.02863
H	4.78418	1.2585	6.36858	C	-4.47348	-0.16792	5.04909
C	4.21802	-0.45424	7.50184	H	-3.77689	-0.22989	4.19893
H	3.95768	0.10601	8.4026	C	-4.17032	0.68698	6.11264
C	4.07808	-1.88172	7.52878	C	-5.05048	0.73463	7.2304
H	3.72215	-2.35271	8.44687	C	-6.2119	-0.07327	7.25277
C	4.40843	-2.62235	6.43351	C	-6.53793	-0.86559	6.12708

H	4.32233	-3.71233	6.44958	C	-7.29151	-4.96432	-0.03135
N	-5.07876	-4.93611	-1.73578	H	-8.1581	-4.97194	0.64411
C	5.95435	-0.70087	2.9285	C	-7.29971	-5.71082	-1.21233
C	5.29285	-2.76311	4.10636	H	-8.17443	-6.31522	-1.48253
C	1.31597	6.29781	1.39646	C	3.7794	4.76565	-3.0547
H	1.25902	5.30283	0.92587	H	3.66132	5.48635	-2.24911
C	-5.68236	-3.98494	2.73519	C	2.94379	4.7952	-4.13218
C	5.85073	-2.12911	2.97273	H	2.14084	5.53147	-4.20437
C	4.75166	2.40772	3.54441	C	-5.00367	-5.95404	4.01457
H	3.81117	2.02957	3.11419	C	0.01337	-6.28836	2.02541
C	-2.34183	6.87447	1.7581	C	-1.30219	-5.98742	2.54039
H	-1.58546	7.01103	2.52669	H	-2.1594	-6.09815	1.87921
C	-3.02668	2.90822	5.86989	C	-1.4775	-5.59925	3.83584
C	-1.93523	6.87979	0.37323	H	-2.47361	-5.38746	4.23316
C	-1.83015	3.69123	5.79234	C	-0.35385	-5.46914	4.71899
C	-5.56248	-3.17912	3.91275	H	-0.53622	-5.1837	5.75554
C	5.98277	0.75016	-4.94054	C	0.90568	-5.71643	4.26256
C	4.95708	-0.18596	-4.89465	H	1.77012	-5.63039	4.9276
H	4.20378	-0.10364	-4.10567	C	1.13972	-6.12264	2.89497
C	4.8479	-1.23943	-5.82163	C	-4.87399	-5.14658	5.1939
C	5.86152	-1.38774	-6.78891	H	-4.58792	-5.63278	6.12738
C	6.92361	-0.43708	-6.86408	C	-0.48162	1.65362	6.03645
C	6.97584	0.64123	-5.95913	C	-0.57307	3.05152	5.8627
C	-2.56227	6.60314	-1.99507	C	-3.72645	0.6278	-5.0755
N	4.93772	3.76511	3.57832	C	-2.60433	-0.08243	-5.6456
C	-5.89996	-1.8081	3.86129	H	-2.69214	-1.15486	-5.80593
C	6.80571	2.83128	-1.84397	C	-1.46682	0.5738	-6.01448
C	5.5447	0.06167	4.04366	H	-0.62452	0.0384	-6.45942
C	-4.37222	4.94285	5.65104	C	-1.36062	1.99355	-5.84877
H	-5.33275	5.45722	5.60688	H	-0.45222	2.49051	-6.18649
C	-6.54148	-2.04635	1.49788	C	-2.40117	2.69933	-5.32128
C	-7.08925	-1.44611	0.30478	H	-2.34421	3.78695	-5.22542
H	-7.1512	-2.04522	-0.59968	C	-3.61774	2.04472	-4.89914
C	-7.55573	-0.1637	0.31751	H	-5.47576	-6.00797	1.93658
H	-7.9857	0.29062	-0.5771	H	-4.80977	-7.02529	4.09924
C	-7.49278	0.62426	1.51447	O	-7.65213	-1.61084	5.93851
H	-7.88412	1.64169	1.48936	O	-7.07302	-0.02271	8.34092
C	-6.94547	0.10462	2.64941	O	-4.63811	1.60564	8.18313
H	-6.88732	0.69683	3.56566	O	5.98275	-2.35201	-7.73983
C	-6.44613	-1.25067	2.68446	O	7.99756	-0.61853	-7.71197
N	-2.61995	-6.31922	-1.76329	O	7.91093	1.62008	-5.89814
C	-1.55099	-6.02985	-0.95637	O	4.25463	-8.32939	4.08582
H	-1.18686	-4.98534	-0.96776	O	6.27811	-7.37878	5.52124
C	-0.95771	-7.02882	-0.15854	O	6.91904	-4.57282	5.38439
C	-1.49145	-8.33195	-0.19501	O	-5.90975	4.52051	-6.03792
H	-1.04493	-9.12101	0.4246	O	-5.74927	7.428	-5.70752
C	-2.58129	-8.61317	-1.02085	O	-3.88795	8.32777	-3.6212
H	-3.0055	-9.62399	-1.06164	C	5.14469	-3.53553	-7.70518
C	-3.12937	-7.59269	-1.80285	C	7.68038	-0.66483	-9.13303
H	-3.98305	-7.76785	-2.47452	C	8.98029	1.65867	-6.89046
C	-6.15997	-4.19453	0.29414	C	3.02997	-8.97103	3.64587
C	5.98343	1.83818	-3.93901	C	6.90643	-8.50846	4.84952
C	5.73428	1.52724	4.04136	C	7.87408	-5.40136	6.11357

C	5.74967	3.76802	-1.88493	C	-8.78429	-1.48565	6.8499
C	-5.13276	-3.80941	5.13974	C	-6.78574	-1.03504	9.35245
H	-5.04757	-3.1868	6.03322	C	-5.50254	1.93028	9.31147
C	-4.29917	3.58765	5.7802	C	-6.45057	5.27872	-7.16113
H	-5.20898	2.98445	5.83662	C	-7.07269	7.80825	-5.22295
C	-1.94875	5.12354	5.65165	C	-4.3423	9.39035	-4.51112
H	-1.04196	5.72118	5.61185	H	5.66838	-4.18267	-8.4274
N	-1.99407	-5.16012	-4.24167	H	4.14097	-3.28715	-8.05989
C	-2.08722	-6.20066	-5.13212	H	5.14387	-3.98996	-6.71145
H	-2.97754	-6.84239	-5.056	H	7.16484	0.24496	-9.44269
C	-1.08804	-6.42689	-6.08179	H	7.09521	-1.56422	-9.35787
H	-1.17423	-7.26702	-6.78141	H	8.68336	-0.7342	-9.57576
C	0.01505	-5.57236	-6.12737	H	8.56033	1.8506	-7.88111
H	0.80978	-5.72996	-6.86825	H	9.54763	0.71958	-6.87501
C	0.10994	-4.50009	-5.21857	H	9.57603	2.50564	-6.52571
C	-0.91382	-4.32033	-4.26748	H	3.02151	-9.04128	2.55509
H	-0.88367	-3.51423	-3.50617	H	2.15026	-8.46117	4.04573
C	-2.92946	6.64066	-0.63109	H	3.14157	-9.96776	4.10317
C	1.28097	-3.60432	-5.30886	H	7.46834	-8.96494	5.67588
C	1.35448	-2.67478	-6.37088	H	7.56775	-8.16411	4.05364
C	0.26288	-2.48245	-7.29684	H	6.13974	-9.19762	4.47634
H	-0.63943	-3.07708	-7.17094	H	7.35679	-5.99553	6.87741
C	0.35812	-1.58829	-8.3232	H	8.41759	-6.04272	5.41506
H	-0.45775	-1.45556	-9.03795	H	8.52223	-4.63498	6.55811
C	1.54356	-0.7981	-8.49349	H	-9.60712	-1.85665	6.22353
H	1.58435	-0.09404	-9.32698	H	-8.94237	-0.44559	7.15651
C	2.59232	-0.94585	-7.6362	H	-8.61076	-2.13489	7.71274
H	3.50272	-0.35275	-7.75948	H	-6.9075	-2.03726	8.93403
C	2.54278	-1.89589	-6.54826	H	-7.55501	-0.8222	10.10694
C	6.90711	-0.84374	0.68165	H	-5.78093	-0.89454	9.75884
H	7.30746	-0.37954	-0.22364	H	-6.5465	2.04554	8.99841
C	-3.65367	6.72515	2.10293	H	-5.0684	2.88097	9.64884
H	-3.96864	6.73962	3.14809	H	-5.39738	1.14883	10.06961
C	-1.25121	6.9674	-2.38125	H	-5.75432	6.05696	-7.49368
C	-0.88156	7.11062	-3.77097	H	-7.41296	5.70516	-6.86444
H	-1.6443	6.91473	-4.5284	H	-6.57227	4.48941	-7.91506
C	0.36752	7.5183	-4.13318	H	-7.72131	6.93146	-5.15705
H	0.63567	7.66451	-5.18058	H	-7.41	8.4958	-6.01053
C	1.35456	7.79481	-3.12926	H	-6.99488	8.31754	-4.25896
H	2.33836	8.14003	-3.45417	H	-5.37016	9.65423	-4.24575
C	1.05878	7.64158	-1.80636	H	-3.6409	10.19709	-4.25722
H	1.8056	7.86123	-1.04765	H	-4.26306	9.09504	-5.56339

Table S13. Cartesian coordinates of **WF** (E_{opt} = –0.120230694354 and E_{sp} = –1164.24944657 hartree).

C	-0.09189	-2.81456	-1.12483	H	-0.39397	-0.64609	0.5213
C	-1.34085	-1.94488	-0.85306	H	-2.99127	0.14256	-0.5605
N	0.47681	-3.44464	0.08223	H	-1.52033	0.88099	2.08817
O	-2.40874	-2.17377	-1.42223	H	-3.0413	1.59737	1.59979
N	-1.28075	-0.95193	0.11761	C	0.9815	-2.0335	-1.91081
C	-2.47339	-0.12142	0.40739	C	3.77579	0.8985	1.09746
C	-2.10421	1.15209	1.17853	H	3.91424	1.93624	1.38035
C	-0.50187	3.06923	1.03906	C	4.54429	-0.11501	1.63789

H	-0.30364	2.94025	2.10259	H	5.31286	0.11863	2.37442
C	0.12698	4.10183	0.33983	C	4.36443	-1.47229	1.25431
H	0.80446	4.77624	0.86305	H	5.00437	-2.22795	1.70675
C	-0.12204	4.28185	-1.02496	C	3.40798	-1.839	0.32584
H	0.36333	5.09113	-1.56761	H	3.27532	-2.87861	0.02456
C	-1.01302	3.43001	-1.68422	C	2.59451	-0.83767	-0.24168
H	-1.21965	3.57404	-2.74476	C	2.79918	0.52926	0.14784
C	-1.65227	2.40116	-0.98654	C	1.526	-0.84421	-1.21276
H	-2.35675	1.75384	-1.50932	C	1.12527	0.47389	-1.39854
C	-1.39894	2.21061	0.3799	N	1.89088	1.31836	-0.57449
C	-3.46063	-0.95955	1.26314	H	0.36215	0.86766	-2.04959
O	-4.13431	-1.9396	0.61086	H	1.77532	2.31347	-0.49921
O	-3.69255	-0.77961	2.43193	H	0.56262	-1.74556	-2.89926
H	-0.46078	-3.65613	-1.78859	H	1.81605	-2.73997	-2.13828
H	-0.23208	-3.93358	0.62198	H	-3.85267	-2.09886	-0.35986
H	0.93699	-2.7723	0.68947				

Table S14. Cartesian coordinates of **WW** (E_{opt} = –0.102879452958 and E_{sp} = –1295.76819332 hartree).

C	2.57147	-0.7699	-1.86284	C	0.48839	-2.15902	1.09864
C	1.59732	-1.68615	-1.08588	C	1.27668	-2.48561	2.37362
N	3.25859	-1.51435	-2.94359	O	1.88038	-1.76299	3.12612
O	1.10187	-2.69131	-1.57561	H	1.60707	-0.4086	0.59821
H	3.35704	-0.37273	-1.1637	H	0.238	-3.12468	0.56071
H	2.5831	-1.94396	-3.57529	C	-3.26853	0.70459	1.32409
H	3.82239	-2.26973	-2.56008	H	-2.77358	0.7907	2.28889
C	1.79646	0.41675	-2.47938	C	-4.34808	1.50582	0.99548
C	0.96905	3.54696	1.38995	H	-4.71624	2.24495	1.70454
H	0.16501	3.969	1.98293	C	-5.0106	1.37876	-0.25465
C	2.29494	3.85279	1.63468	H	-5.85955	2.02996	-0.46186
H	2.55533	4.53174	2.44614	C	-4.60634	0.45199	-1.19804
C	3.34364	3.30314	0.84867	H	-5.11415	0.34698	-2.15118
H	4.36884	3.58332	1.0834	C	-3.50308	-0.36325	-0.87042
C	3.08321	2.43343	-0.19502	C	-2.81957	-0.24592	0.38827
H	3.88572	2.02262	-0.80403	N	-2.85728	-1.36392	-1.61209
C	1.74639	2.08814	-0.47216	C	-1.79273	-1.87558	-0.84125
C	0.69808	2.65989	0.32719	C	-1.74444	-1.21324	0.37641
C	1.11308	1.23281	-1.44771	C	-0.80339	-1.42417	1.50497
C	-0.25795	1.30466	-1.23636	H	-3.12601	-1.69205	-2.51918
N	-0.52435	2.17159	-0.16115	H	-1.13727	-2.6482	-1.22768
H	-1.05481	0.8025	-1.7606	H	-0.55185	-0.4445	1.97192
H	-1.43627	2.36344	0.21353	H	-1.32383	-2.00856	2.29979
H	1.07258	0.04821	-3.23604	O	1.20209	-3.83546	2.6098
H	2.5186	1.04523	-3.05081	H	1.69866	-4.11785	3.42494
N	1.38054	-1.34633	0.25159				

Table S15. Cartesian coordinates of **1•WF** (R = OCH_3; E_{opt} = 1.64042617676 and E_{sp} = –9984.59031571 hartree).

Pt	6.97242	-0.3709	1.04531	C	-4.37394	-2.79677	-4.41248
Pt	-7.09539	0.51537	-1.06189	C	1.99513	-5.86072	2.40785
N	7.29809	1.47103	0.36062	C	4.59526	5.01231	-0.84995
C	6.31942	2.15841	-0.30361	C	7.72107	0.30444	3.79634

H	5.34209	1.65141	-0.43318	H	8.66321	-0.1667	3.48131
C	6.54903	3.45533	-0.80145	C	2.81468	0.03188	-5.84884
C	7.80987	4.04631	-0.59324	H	2.2496	0.72901	-6.46586
H	8.00567	5.05882	-0.97326	C	4.33605	4.55937	-5.10322
C	8.79906	3.33599	0.09018	H	3.54436	5.07697	-5.65146
H	9.78799	3.78061	0.25929	C	-3.21712	4.12968	4.886
C	8.52681	2.04519	0.55855	C	3.99632	2.0986	4.82598
H	9.27813	1.45048	1.09788	C	8.73316	-1.69152	-2.59618
N	6.71123	0.36687	2.87352	H	9.74798	-1.72836	-3.01214
N	6.73036	-2.22101	1.7527	C	2.81258	6.76458	0.49162
C	7.84026	-2.99758	1.95659	H	2.1629	7.44944	1.03523
H	8.81543	-2.55685	1.70069	C	2.6814	6.5792	-0.85243
C	7.72349	-4.29532	2.46844	H	1.91582	7.11372	-1.42
H	8.62312	-4.90467	2.62288	C	0.31852	3.89141	6.18513
C	6.46111	-4.80009	2.78429	C	-0.82549	3.7742	5.39161
H	6.34957	-5.81324	3.19511	H	-0.76025	3.23064	4.4432
C	5.31864	-4.00062	2.58027	C	-2.0468	4.33779	5.76383
C	5.48489	-2.7078	2.05091	C	-2.12611	5.08396	6.97348
H	4.6331	-2.03282	1.85356	C	-1.00405	5.1488	7.83324
C	-5.57625	-3.45101	2.32428	C	0.22688	4.58122	7.42834
N	-7.29185	1.93589	0.32243	C	-2.62894	-2.78545	-6.14075
C	-8.47989	2.61672	0.39388	C	-3.87127	-2.29006	-5.63164
H	-9.26501	2.33643	-0.32278	C	-4.57247	-1.28009	-6.38861
C	-8.67239	3.62702	1.34308	H	-5.52426	-0.91218	-6.01438
H	-9.62997	4.16228	1.38553	C	-4.06615	-0.80319	-7.5631
C	-7.64179	3.94396	2.22986	H	-4.6008	-0.05068	-8.14713
H	-7.77136	4.7338	2.98379	C	-2.81272	-1.28589	-8.06589
C	-6.42258	3.24198	2.15896	H	-2.43897	-0.88103	-9.00808
C	-6.27369	2.23553	1.18596	C	-2.12268	-2.23993	-7.37926
H	-5.33176	1.6547	1.09107	H	-1.16793	-2.61761	-7.75556
C	4.68056	-0.30823	-4.32887	C	3.76637	-3.39135	6.50073
H	5.53966	0.07268	-3.78127	H	4.3489	-2.8648	7.2601
C	3.77362	-4.4958	-4.2371	C	0.5651	1.01289	5.91832
C	4.90174	-3.99912	-3.50869	H	-0.32272	1.46205	6.37163
C	5.73062	-4.93851	-2.79038	C	3.56195	5.69006	-1.57536
H	6.59481	-4.56361	-2.24741	C	6.32562	1.4112	5.43031
C	5.46987	-6.27744	-2.82737	H	6.16826	1.8227	6.43661
H	6.11758	-6.99574	-2.31968	C	6.32148	3.16911	-5.04628
C	4.33634	-6.77801	-3.54928	H	7.06741	2.60832	-5.61366
H	4.16399	-7.8556	-3.56774	C	1.30588	-6.72613	1.47857
C	3.51728	-5.91828	-4.21885	H	0.35317	-7.16096	1.79329
H	2.65818	-6.29522	-4.78095	C	0.62655	-0.33383	5.71142
N	-7.46343	-0.89245	0.30672	H	-0.19953	-0.98654	5.9887
C	4.39147	-1.72221	-4.27293	C	-2.30935	-5.78152	3.90416
C	2.97108	-3.60421	-4.98686	C	-1.01398	-5.43103	3.52085
C	5.51314	0.94376	3.19793	H	-0.8812	-4.61425	2.80343
H	4.73924	0.96109	2.407	C	0.11175	-6.07334	4.04326
C	-5.37111	-4.7661	1.85235	C	-0.06927	-7.17494	4.92684
C	3.27553	-2.22372	-5.01666	C	-1.37488	-7.59379	5.27513
C	6.19247	-1.56911	-1.4921	C	-2.49381	-6.85809	4.81799
H	5.19922	-1.4936	-1.00735	C	-8.01127	-2.85119	2.22196
C	2.90331	-0.14759	4.88726	H	-8.22175	-3.62195	2.97695
H	3.82242	-0.60826	4.53184	C	-9.04226	-2.07134	1.69255

C	2.20532	-4.81948	4.6151	H	-10.07848	-2.21945	2.02318
C	2.87441	1.28361	5.08663	C	4.69069	5.22262	0.5758
C	3.4853	-4.2929	4.24496	H	5.47465	4.70991	1.12744
C	-4.29117	-5.54611	2.38038	C	3.83497	6.06873	1.21871
C	2.30387	6.10191	-3.70517	H	3.91221	6.24016	2.29477
C	1.03436	5.55084	-3.58498	C	-6.01644	-6.6367	0.41508
H	0.88228	4.72884	-2.87777	C	-3.66124	-3.77253	-3.68275
C	-0.06595	6.00225	-4.3408	C	-4.13285	-4.28007	-2.41589
C	0.14629	7.04429	-5.26703	H	-5.06483	-3.8944	-2.01072
C	1.4218	7.67589	-5.36023	C	-3.43211	-5.23266	-1.73547
C	2.49501	7.22227	-4.57008	H	-3.78414	-5.61299	-0.77247
C	1.58303	3.287	5.71823	C	-2.21546	-5.76788	-2.27495
N	7.26594	-1.1096	-0.77864	H	-1.69975	-6.54834	-1.71718
C	-3.45547	-5.00467	3.38176	C	-1.74131	-5.31705	-3.47062
C	5.41852	4.03436	-2.94551	H	-0.8251	-5.72904	-3.90223
C	5.18341	-2.61588	-3.52173	C	-2.4352	-4.28883	-4.21171
C	2.48099	-3.90974	6.87144	C	-4.93025	-7.41632	0.93574
H	2.13486	-3.75814	7.89541	H	-4.80042	-8.43583	0.5692
C	-4.75053	-2.90873	3.33315	C	3.26673	-5.32074	2.02894
C	-4.95335	-1.57635	3.8546	C	3.9837	-4.51946	2.9426
H	-5.76475	-0.9755	3.4513	C	-4.31641	4.46013	2.70808
C	-4.16086	-1.08067	4.84775	C	-4.31373	5.10043	1.4136
H	-4.31889	-0.07643	5.24893	H	-5.10854	4.86285	0.71146
C	-3.10854	-1.87891	5.40667	C	-3.34809	6.00479	1.08028
H	-2.52393	-1.4647	6.22641	H	-3.34471	6.49533	0.10378
C	-2.88623	-3.14024	4.93991	C	-2.31402	6.3461	2.01385
H	-2.10804	-3.76901	5.37909	H	-1.58208	7.10075	1.7262
C	-3.68653	-3.69881	3.87451	C	-2.27583	5.75103	3.23953
N	-6.97129	-0.8938	-2.46538	H	-1.50661	6.02024	3.96794
C	-5.7612	-1.35261	-2.90809	C	-3.26252	4.7687	3.62604
H	-4.84658	-0.93644	-2.43252	H	-7.0476	-4.77372	0.44964
C	-5.68139	-2.32579	-3.92216	H	-6.67163	-7.09666	-0.3282
C	-6.87653	-2.83483	-4.46739	O	-3.79162	-7.04519	5.15331
H	-6.83305	-3.59828	-5.25615	O	-1.55329	-8.66654	6.13822
C	-8.1051	-2.36393	-4.00024	O	1.10441	-7.70031	5.35111
H	-9.04541	-2.75234	-4.41246	O	-0.74579	7.60169	-6.13081
C	-8.13428	-1.38729	-2.99854	O	1.68456	8.62909	-6.32428
H	-9.07924	-0.98696	-2.60445	O	3.76231	7.70048	-4.54947
C	-6.68908	-2.64696	1.78317	O	-1.43612	-5.80354	-7.75997
C	3.43884	5.50023	-2.96908	O	1.03249	-6.41893	-8.50868
C	6.35357	-2.10998	-2.77996	O	3.2621	-5.11813	-7.22388
C	5.48908	4.16719	-1.54078	O	-3.3358	5.66159	7.15684
C	-4.09981	-6.888	1.87827	O	-1.08766	5.84254	9.03305
H	-3.27569	-7.47588	2.28846	O	1.40258	4.60895	8.09967
C	1.72892	-4.58928	5.96029	C	-1.98598	6.92533	-6.45832
H	0.75454	-4.99944	6.23755	C	0.88769	9.84554	-6.23815
C	4.24205	-3.56048	5.2327	C	4.11271	8.8487	-5.37929
H	5.22114	-3.1745	4.95894	C	-2.81479	-5.82505	-7.31046
N	-6.72086	1.92131	-2.43239	C	0.42104	-6.11953	-9.79659
C	-7.73551	2.32283	-3.26027	C	3.59335	-5.91734	-8.39874
H	-8.71529	1.8417	-3.12815	C	-4.14851	-7.9544	6.23725
C	-7.52054	3.30554	-4.23428	C	-1.71224	-9.94307	5.44822
H	-8.3453	3.61368	-4.89009	C	1.12503	-8.71405	6.39959

C	-6.25726	3.88357	-4.3625	C	-3.52477	6.65663	8.20771
H	-6.07048	4.65524	-5.12195	C	-1.5264	5.01367	10.15144
C	-5.2096	3.47488	-3.51134	C	1.55622	5.43697	9.29046
C	-5.4755	2.48258	-2.54787	H	-2.26284	7.43767	-7.39414
H	-4.6909	2.12854	-1.86102	H	-2.7234	7.12004	-5.67505
C	1.66668	1.88047	5.57088	H	-1.82611	5.85962	-6.63675
C	-3.87133	4.08239	-3.64457	H	1.01453	10.32052	-5.26453
C	-3.65564	5.39926	-3.17856	H	-0.16396	9.62088	-6.45062
C	-4.68704	6.147	-2.49958	H	1.32632	10.44892	-7.04442
H	-5.65668	5.68024	-2.3448	H	3.56889	9.7328	-5.03669
C	-4.4652	7.42379	-2.07028	H	3.89913	8.63555	-6.4341
H	-5.25117	8.00086	-1.57666	H	5.1903	8.9284	-5.18531
C	-3.18623	8.04405	-2.25984	H	-3.28668	-4.8623	-7.52408
H	-3.04283	9.06412	-1.89792	H	-2.88397	-6.10851	-6.25757
C	-2.18573	7.36775	-2.89177	H	-3.23094	-6.61759	-7.95342
H	-1.20631	7.8315	-3.04096	H	0.78394	-6.95645	-10.40827
C	-2.38492	6.02578	-3.39	H	0.77278	-5.15961	-10.17642
C	3.92123	0.5333	-5.08838	H	-0.67132	-6.14934	-9.70861
H	4.14825	1.60097	-5.14785	H	3.17353	-6.92655	-8.30316
C	1.81736	-0.92147	5.17362	H	3.2256	-5.42071	-9.30038
H	1.84378	-2.00436	5.03116	H	4.68979	-5.92088	-8.35006
C	2.68453	4.10399	5.37675	H	-5.14631	-7.58416	6.509
C	2.61637	5.54612	5.45637	H	-3.4441	-7.88016	7.07346
H	1.67362	6.00039	5.77075	H	-4.19069	-8.9725	5.83998
C	3.6953	6.32465	5.16133	H	-2.60489	-9.92756	4.81816
H	3.64854	7.41268	5.23558	H	-1.83037	-10.63899	6.28988
C	4.93204	5.71886	4.76072	H	-0.81841	-10.17544	4.86371
H	5.78356	6.37107	4.55204	H	0.40811	-8.48362	7.19581
C	5.03483	4.36259	4.65118	H	2.1638	-8.64083	6.7491
H	5.97429	3.90921	4.3434	H	0.9208	-9.68919	5.94794
C	3.91381	3.50359	4.95156	H	-2.64735	7.3048	8.31282
C	-1.93159	-3.79474	-5.43657	H	-3.75216	6.13671	9.14249
C	4.37566	4.69997	-3.66567	H	-4.39608	7.20793	7.82814
C	-1.36648	5.3435	-4.09644	H	-2.53305	4.62946	9.96989
C	7.64666	-2.16519	-3.33472	H	-1.51891	5.73478	10.98021
H	7.7904	-2.58176	-4.34129	H	-0.81683	4.20096	10.32617
C	7.54353	0.82293	5.08477	H	1.15557	4.89187	10.14994
H	8.3614	0.76423	5.81384	H	2.64901	5.53272	9.34621
C	-8.75069	-1.09407	0.73448	H	1.06648	6.41014	9.16986
H	-9.53335	-0.45912	0.29638	C	1.64135	-2.99742	-1.35054
C	1.46204	-5.58577	3.68788	C	1.03573	-1.75177	-2.01041
C	-5.32041	2.95881	4.37446	O	0.05615	-1.88931	-2.74057
C	-4.24449	3.24039	5.27617	N	1.46715	-0.43894	-1.82414
C	-4.25515	2.61028	6.57663	C	2.77419	0.04499	-1.3846
H	-3.43171	2.82745	7.26039	C	2.92336	1.55209	-1.70739
C	-5.27141	1.78491	6.95611	C	0.63594	2.61725	-1.65084
H	-5.29267	1.32607	7.94589	H	0.42315	2.18925	-2.63188
C	-6.35645	1.51828	6.05658	C	-0.34373	3.38117	-1.009
H	-7.16406	0.86801	6.40037	H	-1.309	3.53758	-1.49178
C	-6.37373	2.07192	4.8094	C	-0.08502	3.94925	0.2415
H	-7.20167	1.87236	4.13387	H	-0.84994	4.54445	0.7414
C	1.83636	-7.01593	0.25668	C	1.16103	3.75664	0.8468
H	1.33197	-7.68936	-0.43686	H	1.37422	4.21439	1.81325

C	8.52535	-1.16803	-1.3142	C	2.1414	2.98788	0.21333
H	9.35311	-0.78841	-0.69846	H	3.1112	2.8609	0.6895
C	6.38034	3.25191	-3.68515	C	1.88023	2.40773	-1.0369
H	7.18184	2.75901	-3.1408	N	2.85178	-2.71094	-0.53651
C	2.50863	-1.29626	-5.81653	H	0.78601	0.28456	-2.10174
H	1.68147	-1.69216	-6.41174	C	3.06977	-0.12847	0.11581
C	5.27553	3.83058	-5.77052	H	2.88448	1.68881	-2.8138
H	5.26534	3.75204	-6.85906	H	3.94823	1.89387	-1.43069
C	1.79666	-4.11184	-5.72673	C	0.49808	-3.66685	-0.54142
C	1.96236	-4.93033	-6.88591	C	-2.49251	-0.32038	1.77477
C	0.83076	-5.47675	-7.51946	H	-2.64307	0.15813	2.73857
C	-0.4754	-5.16916	-7.03617	C	-3.29211	-0.0414	0.68391
C	-0.63657	-4.28455	-5.95076	H	-4.09309	0.69905	0.77583
C	0.51199	-3.79253	-5.30282	C	-3.09439	-0.68529	-0.56788
H	0.37651	-3.12887	-4.43388	H	-3.74853	-0.42886	-1.40328
C	-5.32299	3.54849	3.09142	C	-2.07897	-1.60808	-0.75276
C	3.10039	-6.45862	-0.12783	H	-1.91088	-2.08923	-1.72348
H	3.50174	-6.72074	-1.11036	C	-1.23186	-1.91455	0.33032
C	-6.44475	-1.65366	0.81423	C	-1.45739	-1.26516	1.59235
H	-5.42151	-1.46583	0.44074	C	-0.09387	-2.79075	0.5024
C	5.2886	1.47907	4.47844	C	0.32775	-2.66572	1.82083
C	3.78583	-5.63715	0.71923	N	-0.49433	-1.73571	2.49366
H	4.76008	-5.24443	0.43467	H	1.12992	-3.17348	2.33034
C	-2.84006	3.37371	-4.2999	H	-0.42309	-1.48808	3.46455
C	-1.58139	4.01607	-4.53718	H	0.8741	-4.61151	-0.09132
C	-0.56081	3.28506	-5.25313	H	-0.30163	-3.98219	-1.25672
H	0.38268	3.79591	-5.46533	O	1.99222	-0.17677	0.91627
C	-0.7654	2.00262	-5.66656	O	4.20093	-0.1627	0.60536
H	0.00311	1.44959	-6.20624	H	3.58208	-0.54503	-1.92109
C	-2.02006	1.35549	-5.41316	H	2.20199	-0.24719	1.89281
H	-2.1545	0.32673	-5.76107	H	1.92603	-3.68253	-2.20328
C	-3.02033	2.01807	-4.7633	H	3.54572	-3.44582	-0.68857
H	-3.97689	1.52971	-4.59687	H	2.61861	-2.73464	0.45819
C	-6.22152	-5.3585	0.84644				

Table S16. Cartesian coordinates of **1•WW** (R = OCH_3; E_{opt} = 1.66542790694 and E_{sp} = –10116.1070376 hartree).

Pt	6.88537	-0.28119	1.49119	C	3.55288	-5.24608	0.42838
Pt	-6.91312	0.27389	-1.54575	C	8.79399	-0.14751	-0.74676
N	6.82562	-2.27877	1.55889	H	9.50063	0.04855	0.07258
C	5.62046	-2.92914	1.57827	C	0.40418	-2.7068	5.52923
H	4.71122	-2.30037	1.55258	H	-0.32747	-3.5128	5.51394
C	5.54476	-4.33353	1.63004	C	1.93041	-6.64669	4.13837
C	6.74399	-5.07004	1.66907	H	0.97849	-7.18304	4.13836
H	6.70863	-6.16695	1.71245	C	-1.00701	-1.61289	-6.64479
C	7.96774	-4.39655	1.65481	C	5.87824	-0.88547	-3.76977
H	8.91067	-4.95608	1.68838	C	7.09121	-0.46927	5.70627
C	7.99106	-2.99792	1.59954	H	7.89353	-0.662	6.42857
H	8.93247	-2.43035	1.5874	C	2.17464	-5.75662	-1.99976
N	7.4817	-0.34672	-0.40956	H	1.68153	-5.94766	-2.95079
N	6.97553	1.7149	1.46262	C	1.64043	-6.19011	-0.82307
C	8.10933	2.30345	1.96651	H	0.70165	-6.74804	-0.80956

H	8.90212	1.63169	2.32564	C	2.77922	-1.5836	-6.68619
C	8.23456	3.69393	2.01914	C	1.45229	-1.49038	-6.26834
H	9.14921	4.14672	2.42035	H	1.23996	-1.25021	-5.21982
C	7.1862	4.49862	1.56327	C	0.38279	-1.71681	-7.13963
H	7.26357	5.59333	1.6058	C	0.65548	-2.05314	-8.49542
C	6.02293	3.89942	1.04826	C	1.99132	-2.08296	-8.96131
C	5.94832	2.49193	0.99866	C	3.05689	-1.89042	-8.05154
H	5.05987	1.98256	0.57839	C	-4.93983	0.63054	5.69463
C	-4.24442	4.98423	-2.34559	C	-5.88721	0.64835	4.62056
N	-6.53482	-0.55407	-3.32626	C	-6.93456	-0.34561	4.60787
C	-7.59122	-1.01617	-4.0667	H	-7.66902	-0.32106	3.80707
H	-8.59716	-0.90063	-3.63715	C	-7.02748	-1.28319	5.59546
C	-7.3878	-1.611	-5.31792	H	-7.83478	-2.01921	5.60527
H	-8.24803	-1.97248	-5.89728	C	-6.06301	-1.3212	6.65603
C	-6.09145	-1.73632	-5.81906	H	-6.1662	-2.0868	7.42746
H	-5.91038	-2.19948	-6.80009	C	-5.05628	-0.40309	6.69778
C	-5.00249	-1.2596	-5.0617	H	-4.31848	-0.42378	7.50516
C	-5.25742	-0.66739	-3.8101	C	6.0653	5.64179	-2.85267
H	-4.44294	-0.2702	-3.18725	H	6.91175	5.49472	-3.52625
C	2.65783	-1.98077	4.98261	C	3.49577	1.11018	-5.8941
H	3.64231	-2.17603	4.56323	H	2.7716	0.92463	-6.6905
C	1.81333	1.81344	6.79035	C	2.30231	-5.945	0.43771
C	3.11924	1.55712	6.26078	C	8.26508	-0.43576	-3.09335
C	4.11851	2.59592	6.35778	H	8.57061	-0.46829	-4.14762
H	5.11171	2.40555	5.95764	C	3.87406	-5.76284	5.28805
C	3.83839	3.79067	6.95392	H	4.40189	-5.63547	6.23579
H	4.59857	4.56902	7.05048	C	1.70992	6.19019	1.96419
C	2.52816	4.05324	7.47573	H	0.92483	6.87625	1.63894
H	2.34039	5.01857	7.94902	C	3.78688	2.38158	-5.49959
C	1.55513	3.10304	7.39112	H	3.32129	3.24382	-5.97738
H	0.55829	3.29366	7.79605	C	-0.70519	7.30057	-1.50692
N	-6.67428	2.10005	-2.32533	C	0.4064	6.5858	-1.04722
C	2.40572	-0.69422	5.59139	H	0.32957	5.49708	-0.94064
C	0.8234	0.80595	6.73727	C	1.6171	7.20941	-0.74495
C	6.55944	-0.595	-1.38837	C	1.71815	8.62449	-0.88136
H	5.51841	-0.75154	-1.05421	C	0.59678	9.3741	-1.30635
C	-4.23465	5.85688	-1.23487	C	-0.59603	8.70784	-1.67511
C	1.10895	-0.44544	6.14774	C	-6.48331	4.66525	-3.41536
C	5.09545	0.00622	3.82886	H	-6.40268	5.67734	-3.83868
H	4.335	0.17674	3.03994	C	-7.61018	3.87757	-3.65802
C	5.4099	1.56515	-3.88626	H	-8.43336	4.25745	-4.27725
H	6.14799	1.74596	-3.10625	C	4.08945	-4.81004	-0.84012
C	3.84978	6.13256	-1.1521	H	5.05341	-4.30654	-0.85238
C	5.15935	0.20516	-4.30471	C	3.42429	-5.05371	-2.00573
C	4.93196	5.30199	-0.7113	H	3.83142	-4.73187	-2.96648
C	-3.07071	6.6481	-0.96657	C	-5.36387	6.86925	0.68277
C	0.46697	-7.12993	1.66633	C	-4.76766	2.61515	3.66693
C	-0.72973	-6.46202	1.44584	C	-4.62204	3.62113	2.64236
H	-0.7047	-5.38698	1.23436	H	-5.30062	3.60966	1.79337
C	-1.98071	-7.11624	1.47895	C	-3.65542	4.58006	2.73243
C	-2.01111	-8.48816	1.79635	H	-3.53302	5.3341	1.95002
C	-0.79557	-9.20772	1.99187	C	-2.77934	4.62931	3.86677
C	0.44053	-8.54062	1.90403	H	-2.04539	5.43289	3.9229

C	3.8752	-1.34997	-5.71897	C	-2.88398	3.69315	4.85128
N	6.37851	-0.23345	3.41745	H	-2.23621	3.73498	5.73152
C	-1.95166	6.57193	-1.8276	C	-3.85953	2.62943	4.77382
C	3.70051	-5.48017	2.86485	C	-4.19613	7.65329	0.96138
C	3.39822	0.30492	5.66782	H	-4.22349	8.34674	1.80311
C	4.97945	6.46826	-3.29851	C	3.82612	5.01464	1.45806
H	5.04141	6.91595	-4.29133	C	4.8991	4.74115	0.5832
C	-3.15212	4.94577	-3.23989	C	-2.87062	-2.53703	-5.32176
C	-3.15878	4.11277	-4.41822	C	-3.39408	-3.62971	-4.53506
H	-4.02135	3.48018	-4.60704	H	-4.39471	-3.54116	-4.11836
C	-2.11545	4.1363	-5.29879	C	-2.67354	-4.77525	-4.3564
H	-2.12405	3.52056	-6.19989	H	-3.07726	-5.61578	-3.7859
C	-0.98035	4.97769	-5.0663	C	-1.36349	-4.91041	-4.9233
H	-0.17498	4.98802	-5.79952	H	-0.82802	-5.84982	-4.77994
C	-0.92474	5.75701	-3.94819	C	-0.82482	-3.88934	-5.64837
H	-0.05787	6.39774	-3.75807	H	0.1632	-3.98964	-6.10742
C	-2.00286	5.76159	-2.98582	C	-1.55154	-2.65992	-5.86507
N	-7.34088	1.09469	0.22347	H	-6.26672	5.40432	-0.5734
C	-6.43384	1.09307	1.24978	H	-6.23719	6.99906	1.32541
H	-5.44616	0.64652	1.05959	O	-1.71771	9.26444	-2.19469
C	-6.75314	1.64837	2.50477	O	0.69302	10.75258	-1.44577
C	-8.02781	2.22135	2.68472	O	2.95035	9.09205	-0.5722
H	-8.29322	2.66356	3.65539	O	-3.09725	-9.29962	1.92068
C	-8.94073	2.22226	1.62866	O	-0.7948	-10.52141	2.41716
H	-9.93712	2.66689	1.75251	O	1.67487	-9.07694	2.05751
C	-8.58123	1.65041	0.40272	O	-4.26256	2.17114	8.80443
H	-9.27202	1.62998	-0.45243	O	-2.19373	1.88358	10.46198
C	-5.43794	4.16083	-2.6166	O	0.32742	0.95564	9.42221
C	1.75863	-6.4032	1.65936	O	-0.46725	-2.3193	-9.20418
C	4.77004	0.02313	5.19919	O	2.25653	-2.38989	-10.2887
C	4.23464	-5.01714	1.64266	O	4.38214	-1.9684	-8.30728
C	-3.0909	7.53811	0.17114	C	-4.42485	-8.7421	2.0925
H	-2.19871	8.13706	0.36991	C	-1.42795	-11.47563	1.51711
C	3.91575	6.70185	-2.4801	C	1.81346	-10.50553	2.32106
H	3.09148	7.34183	-2.80473	C	-5.31016	2.83114	8.04657
C	6.03813	5.07358	-1.61384	C	-3.23828	1.15617	11.1698
H	6.86102	4.44665	-1.27744	C	0.24009	1.04498	10.87611
N	-7.21886	-1.54601	-0.7678	C	-1.69768	10.64179	-2.67503
C	-8.513	-1.95674	-0.58353	C	0.28852	11.48295	-0.24876
H	-9.31049	-1.25545	-0.87023	C	3.3031	10.48071	-0.84362
C	-8.79847	-3.21847	-0.04723	C	-0.37446	-2.90005	-10.53993
H	-9.84272	-3.52794	0.09447	C	2.32399	-1.21544	-11.15307
C	-7.75152	-4.07232	0.30178	C	4.862	-2.45545	-9.59625
H	-7.95602	-5.06687	0.72316	H	-4.97297	-9.62061	2.47121
C	-6.41813	-3.65626	0.11162	H	-4.81894	-8.42674	1.12317
C	-6.18264	-2.37579	-0.42641	H	-4.42749	-7.94079	2.8349
H	-5.16176	-2.00381	-0.5882	H	-0.95647	-11.44928	0.5341
C	4.15975	-0.02936	-5.30262	H	-2.50522	-11.28057	1.46065
C	-5.2996	-4.5516	0.4666	H	-1.23276	-12.42408	2.03594
C	-4.87222	-5.53422	-0.45504	H	1.47318	-11.07663	1.45357
C	-5.51765	-5.71112	-1.73375	H	1.2537	-10.78277	3.22318
H	-6.35986	-5.07232	-1.9873	H	2.89797	-10.58855	2.469
C	-5.11423	-6.6902	-2.59568	H	-5.94624	2.07461	7.57926

H	-5.62685	-6.8546	-3.54672	H	-4.89996	3.54239	7.3262
C	-4.00461	-7.53735	-2.26855	H	-5.85124	3.35942	8.84825
H	-3.70822	-8.30838	-2.98252	H	-3.09616	1.51936	12.19684
C	-3.35737	-7.38689	-1.07803	H	-3.07524	0.08034	11.09655
H	-2.50961	-8.02815	-0.81785	H	-4.22401	1.44897	10.78938
C	-3.78093	-6.39715	-0.11468	H	-0.10148	2.04366	11.17596
C	1.69395	-2.94378	4.9463	H	-0.4281	0.26803	11.25616
H	1.88571	-3.91872	4.49101	H	1.28464	0.86515	11.16167
C	4.75424	2.61131	-4.46446	H	-2.54269	10.63869	-3.37649
H	4.95887	3.64113	-4.16075	H	-0.75277	10.87977	-3.17663
C	4.61804	-2.43442	-5.20316	H	-1.87786	11.3095	-1.82803
C	4.40228	-3.789	-5.66078	H	-0.76289	11.29071	-0.02225
H	3.62263	-3.96067	-6.40621	H	0.44588	12.52492	-0.55839
C	5.16691	-4.82013	-5.20442	H	0.93075	11.21878	0.59535
H	5.02522	-5.83927	-5.56907	H	2.90099	10.81777	-1.80573
C	6.19997	-4.57957	-4.23658	H	4.40017	10.42534	-0.85538
H	6.80307	-5.42781	-3.90404	H	2.94079	11.1013	-0.01917
C	6.41918	-3.32179	-3.75694	H	0.42065	-3.65238	-10.59749
H	7.20796	-3.14065	-3.02958	H	-0.21387	-2.09367	-11.2611
C	5.6444	-2.19846	-4.23048	H	-1.37068	-3.3495	-10.64918
C	-3.93235	1.62176	5.76417	H	1.36239	-0.69691	-11.16786
C	2.44554	-6.17288	2.87284	H	2.54744	-1.66924	-12.12849
C	-3.16703	-6.29674	1.15682	H	3.12951	-0.54986	-10.8322
C	5.78657	-0.22633	6.14153	H	4.84544	-1.62751	-10.31091
H	5.54644	-0.22534	7.21355	H	5.89028	-2.74987	-9.34396
C	9.20241	-0.19161	-2.08619	H	4.26921	-3.3058	-9.95185
H	10.25862	-0.03171	-2.33761	C	1.32537	3.23333	-2.04018
C	-7.69024	2.59612	-3.10222	C	2.36376	2.16804	-1.63169
H	-8.5616	1.94674	-3.2671	N	1.82901	4.06036	-3.15651
C	2.76622	6.39637	-0.28587	O	3.48177	2.47822	-1.23991
C	-3.11269	-0.35402	-6.41512	N	2.02649	0.80914	-1.65865
C	-1.78226	-0.46809	-6.93509	C	3.04638	-0.16489	-1.25166
C	-1.28312	0.58652	-7.78844	C	3.25933	-0.09511	0.27286
H	-0.26451	0.49583	-8.17372	O	4.34225	-0.35736	0.78546
C	-2.06378	1.65113	-8.12693	O	2.28372	0.26875	1.12688
H	-1.7001	2.43301	-8.79628	H	1.22963	3.91466	-1.13072
C	-3.40234	1.75234	-7.62071	H	1.98688	3.51023	-3.99668
H	-4.00866	2.60701	-7.93116	H	2.70917	4.51737	-2.90699
C	-3.90055	0.79883	-6.78086	H	1.13552	0.44701	-2.00041
H	-4.91591	0.88055	-6.40106	H	4.04518	0.15098	-1.70588
C	1.71305	5.68641	3.23002	H	1.39371	0.50753	0.70462
H	0.94058	5.95389	3.94979	C	-0.07389	2.70521	-2.39605
C	7.37038	-0.46496	4.33671	C	-1.43265	0.74261	2.06238
H	8.3816	-0.64809	3.94515	H	-1.9951	-0.08668	2.48101
C	4.38962	-5.28444	4.11911	C	-0.76751	1.65252	2.86147
H	5.34431	-4.76366	4.11413	H	-0.80502	1.54782	3.95424
C	0.1282	-1.50699	6.11269	C	-0.0309	2.73239	2.30713
H	-0.83908	-1.32693	6.58692	H	0.47199	3.42426	2.98597
C	2.61715	-6.45525	5.29958	C	0.05832	2.91655	0.94009
H	2.23901	-6.82969	6.25246	H	0.62	3.75526	0.52101
C	-0.53231	1.0777	7.26637	C	-0.58989	2.00153	0.08603
C	-0.7732	1.19604	8.67013	C	-1.34398	0.93029	0.66802
C	-2.05179	1.57704	9.12336	C	-0.69228	1.8533	-1.35084

C	-3.10846	1.78848	8.19015	C	-1.48282	0.74154	-1.59991
C	-2.89718	1.56315	6.81542	N	-1.94831	0.20135	-0.37556
C	-1.59396	1.23883	6.3876	H	-1.76903	0.31168	-2.54285
H	-1.40948	1.1179	5.30876	H	-2.2741	-0.74738	-0.2731
C	-3.62628	-1.3746	-5.58382	H	-0.05543	2.1832	-3.37636
C	2.76289	4.80264	3.65096	H	-0.73482	3.59272	-2.5812
H	2.72963	4.40715	4.66904	C	0.78584	-2.27036	0.91374
C	-5.56148	2.86172	-2.08269	H	1.77178	-2.00745	1.29442
H	-4.76768	2.4278	-1.45429	C	-0.24648	-2.5682	1.78496
C	6.91654	-0.63949	-2.74728	H	-0.08196	-2.51362	2.86374
C	3.77748	4.47937	2.80011	C	-1.52353	-2.96137	1.3093
H	4.57853	3.81996	3.12684	H	-2.30285	-3.18237	2.04252
C	-4.71581	-4.46913	1.74952	C	-1.78798	-3.08668	-0.04344
C	-3.65746	-5.36755	2.10339	H	-2.74132	-3.46057	-0.40542
C	-3.11705	-5.30761	3.44265	C	-0.74698	-2.75189	-0.93448
H	-2.33724	-6.02653	3.71341	C	0.54427	-2.32041	-0.47246
C	-3.56996	-4.39304	4.346	N	-0.74082	-2.69983	-2.33812
H	-3.17475	-4.35468	5.3618	C	0.53171	-2.27211	-2.75746
C	-4.60277	-3.46892	3.97713	C	1.33008	-2.02369	-1.64709
H	-4.93893	-2.74325	4.72343	C	2.7518	-1.60769	-1.69701
C	-5.15498	-3.50454	2.7294	H	-1.46012	-3.05594	-2.94778
H	-5.95229	-2.81445	2.46467	H	0.77411	-2.17244	-3.80808
C	-5.37717	5.99122	-0.36258	H	3.35759	-2.32076	-1.08577
C	-5.77578	1.62777	3.60889	H	3.14375	-1.73382	-2.73694
C	2.75792	5.86397	1.02348				
